# A large-scale neutral comparison study of survival models on low-dimensional data

**DOI:** 10.1093/bioinformatics/btag186

**Published:** 2026-04-16

**Authors:** Lukas Burk, John Zobolas, Bernd Bischl, Andreas Bender, Marvin N Wright, Raphael Sonabend

**Affiliations:** Department of Statistics, LMU Munich, Bavaria, 80539, Germany; Munich Center for Machine Learning, Bavaria, 80538, Germany; Leibniz Institute for Prevention Research and Epidemiology—BIPS, Bremen, 28359, Germany; Faculty of Mathematics & Computer Science, University of Bremen, Bremen, 28359, Germany; Department of Cancer Genetics, Institute for Cancer Research, Oslo, 0379, Norway; Department of Biostatistics, Oslo Centre for Biostatistics and Epidemiology (OCBE), University of Oslo (UiO), Oslo, 0371, Norway; Department of Statistics, LMU Munich, Bavaria, 80539, Germany; Munich Center for Machine Learning, Bavaria, 80538, Germany; Department of Statistics, LMU Munich, Bavaria, 80539, Germany; Munich Center for Machine Learning, Bavaria, 80538, Germany; Leibniz Institute for Prevention Research and Epidemiology—BIPS, Bremen, 28359, Germany; Faculty of Mathematics & Computer Science, University of Bremen, Bremen, 28359, Germany; OSPO Now, Harrow, HA1 1UD, United Kingdom

## Abstract

**Motivation:**

This work presents the first large-scale neutral benchmark experiment focused on single-event, right-censored, low-dimensional survival data. Benchmark experiments are essential in methodological research to scientifically compare new and existing model classes through proper empirical evaluation. Existing benchmarks in the survival literature are smaller in scale regarding the number of used datasets and extent of empirical evaluation. They often lack appropriate tuning or evaluation procedures, while other comparison studies focus on qualitative reviews rather than quantitative comparisons. This comprehensive study aims to fill the gap by neutrally evaluating a broad range of methods and providing generalizable guidelines for practitioners.

**Results:**

We benchmark 21 models, ranging from classical statistical approaches to many common machine learning methods, on 34 publicly available datasets. The benchmark tunes models using both a discrimination measure (Harrell’s C-index) and a scoring rule (Integrated Survival Brier Score), and evaluates them across six metrics covering discrimination, calibration, and overall predictive performance. Despite superior average ranks in overall predictive performance from individual learners like oblique random survival forests and likelihood-based boosting, and better discrimination rankings from multiple boosting- and tree-based methods as well as parametric survival models, no method statistically significantly outperforms the commonly used Cox proportional hazards model for either tuning measure. We conclude that while the Cox Proportional Hazards model remains a robust default for low-dimensional, right-censored survival data, more flexible methods may be preferable for specific dataset characteristics.

**Availability and implementation:**

All code, data, and results are publicly available on GitHub https://github.com/slds-lmu/paper_2023_survival_benchmark and archived on Zenodo https://doi.org/10.5281/zenodo.19075310.

## 1 Introduction

Survival analysis is an important branch of statistics for data where the outcome is the time until an event of interest occurs. Such data often exhibit incomplete information about the outcome, for example due to censoring. Traditionally applied in medical research to estimate how patient survival relates to clinical or biological characteristics (features), it has been applied in a broad range of applications across various domains. By effectively incorporating information from both completed and censored cases, survival analysis can yield accurate and informative predictions. This capability is invaluable in fields such as medicine, finance, and in different industrial sectors, where risk prediction under censoring is an important decision making component. Many non-, semi-, and fully parametric methods have been introduced in this field, including Machine Learning adaptations [see Section 3.2 and Wang et al. (2019)]. Throughout this paper, we consider only the right-censored, single-event survival setting, which is very common in practical applications. In a nutshell, right-censoring occurs when some subjects did not experience the event of interest, either due to drop-out or the end of the study (both assumed to be unrelated to the event of interest here). Formally, let $Y_{i}∼F_{Y};Y_{i}>0;i=1,\ldots ,n$ be random variables representing the event times and $C_{i}∼F_{C};\;C_{i}>0$ the censoring time. In right-censored data, we do not observe realizations of $Y_{i}$ but rather of the tuple $\left(T_{i}=\min(Y_{i},C_{i}),D_{i}=I(Y_{i}\leq C_{i})\right)$. The goal of survival analysis is to estimate the conditional distribution $F_{Y|x}$, or quantities derived from it, e.g. the expected survival time E(Y|x), based on realizations of $\left(T_{i},D_{i}\right)$ and features $x_{i}$; see Section 3.3 for definition of prediction types. The observed data is given by tuples $(t_{i},d_{i},x_{i}),\;i=1,\ldots ,n$, where $t_{i}$ is the *observed* outcome time (either event or censoring time, whichever occurred first), $d_{i}$ is the status indicator (0 if observation is censored, 1 if the event of interest was observed) and $x_{i}$ is the feature vector.

**Contributions**: This paper introduces the first neutral, large-scale comparison study for single-event, right-censored survival data with a large number of datasets (34), models (21), tuning measures (2), and evaluation measures (6) included. We benchmark survival techniques in the low-dimensional setting, which represents a type of data that practitioners often encounter. The scale of our study, encompassing diverse datasets (see Table 1) and an extensive evaluation procedure using multiple metrics, provides a robust benchmark for comparing model performance in right-censored, low-dimensional settings. Furthermore, we run a “neutral” benchmark in accordance with the guidelines laid out by Boulesteix et al. (2013) which we further outline in Section 3.1. Based on our review of the literature (see Section 2), there is no other study: (i) with a comparable number of datasets or methods; (ii) that compared methods after sufficient tuning for both discrimination and for overall predictive ability as measured by the integrated survival Brier score (ISBS); or (iii) that neutrally compares methods. Finally, our experiment code is available on GitHub, our collection of datasets is available as an OpenML benchmark suite (Vanschoren et al. 2014) and our hyperparameter search spaces will be available in a forthcoming release of *mlr3tuningspaces* (Becker 2024).

**Table 1:** Performance measures used in the benchmark, including their type and the quantity they evaluate, i.e. continuous ranks (crank) or a survival distribution (distr). Harrell’s C and ISBS are used for primary analysis with remaining results in Supplement E. Models evaluated with each measure were tuned on the corresponding tuning measure.

Measure	Tuned on	Type	Evaluates
Harrell’s C	Harrell’s C	Discrimination	crank
Uno’s C	Harrell’s C	Discrimination	crank
ISBS^a^	ISBS	Scoring Rule	distr
ISLL^b^	ISBS	Scoring Rule	distr
D-Calibration	ISBS	Calibration	distr
α-Calibration^c^	ISBS	Calibration	distr

a Integrated Survival Brier Score.

b Integrated Survival Log-Likelihood.

c van Houwelingen’s α.

## 2 Literature review

The experiments described in this paper provide a comparison of both classical and machine learning (ML) survival models in a low-dimensional setting. We use these broad terms for model classes in analogy to the taxonomy provided by Wang et al. (2019), where the term “classical” refers to semi- and fully parametric methods such as the Cox Proportional Hazards (CPH) and Accelerated Failure Time (AFT) models or derivatives thereof, including penalized variants. “ML” here refers to inherently non-linear and non-parametric methods ranging from tree-based methods including Random Survival Forests (RSFs), boosting approaches such as Gradient Boosting Machines (GBMs) or likelihood-based boosting (CoxBoost), to Survival Support Vector Machines (SSVMs), Artificial Neural Networks (ANNs) and Deep Learning (DL) methods. Although there is no objective and generally accepted definition to determine whether a dataset is “high-dimensional,” we colloquially define it to refer to scenarios where the number of features exceeds the number of observations (p≫n).

Historically, surveys, reviews, and analytical comparisons of survival models can be grouped into: (i) empirical comparisons of models with limited scope; (ii) qualitative surveys without benchmark experiments. We provide a short overview of the literature for comparisons of survival models, with an extended review available in Supplement A. Papers that empirically compare survival models (1) are further separated into studies that: (i) compare ‘classical’ models only; (ii) compare multiple ML and classical model classes; (iii) compare one novel model (or class) to one or more baseline models; and (iv) exclusively focus on high-dimensional data.

**Comparisons of Classical Models** often compare CPH and AFT models, including Moghimi-Dehkordi et al. (2008), Georgousopoulou et al. (2015), Zare et al. (2015), and Habibi et al. (2018), showing both methods yielding similar hazard ratios but their evaluation relies on model coefficients and AIC, without performance evaluation on external data; Dirick et al. (2017) additionally compare flexible Cox models including splines using time-dependent area under the Receiver Operating Characteristic curve (AUC), mean squared error, and mean absolute error, finding that the CPH with penalized splines outperforms the other models.

**Comparisons of ML and Classical Models** The experiments carried out in this paper belong in this category. Only two prior experiments could be found that neutrally benchmarked more than one ML model class on low-dimensional data. Kattan (2003) benchmarked tree-based models, ANNs and CPH with Harrell’s C-index across three datasets. The models are compared for statistically significant differences using 50 times repeated nested cross-validation, but the authors do not clarify their tuning procedure. Boxplots across all replications indicate that no machine learning model outperformed the CPH. The authors note the small number of datasets used for comparison as their primary limitation. Zhang et al. (2021) compare classical and ML methods, taking into account feasibility and computational efficiency for various tasks in the biomedical field. Methods are evaluated on six clinical and 16 omics datasets using 11 metrics, including time-dependent AUC, ISBS and multiple variations of the C-index. Critically, methods were applied with implementation-specific default hyperparameter settings without tuning, severely limiting the generalizability of their results.

**Comparisons of a Novel Model Class** include Luxhoj and Shyur (1997), Ohno-Machado (1997) benchmarking newly developed ANNs against CPH and Goli et al. (2016) comparing modern Support Vector Regression variants against CPH, where neither found statistically significant differences. Jaeger et al. (2024) compare a novel implementation of oblique RSFs (ORSF) to the previous implementation, as well as other RSFs, GBMs, penalized CPH, and ANNs. Using Harrell’s C and ISBS, they find that ORSFs outperform GBMs and penalized CPH, but with only minimal tuning applied for some models.

**Comparisons on High-Dimensional Data** have gained popularity in the survival literature, with many recent studies focusing on the area of multi-omics data (Zhao et al. 2024). Herrmann et al. (2021) perform a large-scale benchmark including penalized regression, GBMs, and RSFs, evaluating on Harrell’s C and ISBS without finding statistically significant differences between the different model classes. Notably, CPH based on purely clinical (low-dimensional) data was not statistically significantly outperformed by other methods including multi-omics data. Spooner et al. (2020) compare a similar group of models evaluating on Harrell’s C only, finding few statistically significant differences. Wissel et al. (2023) compare DL methods, RSFs and CPH, evaluating on Antolini’s C-index and ISBS with a focus on noise-resistance, noting a lack thereof for all models.

## 3 Benchmark experiments

### 3.1 Study design

The experiments in this study are designed to assess the status quo of survival models, including both classical and machine learning approaches. In order to achieve this objective, this study aims to be a “neutral comparison study” (Boulesteix et al. 2013). Following the guidelines put forward by Boulesteix et al. such studies:

**Focus on model comparison**: The focus of this study is on model comparison rather than on the examination of a novel model. We do not favour one dataset or model over another and draw conclusions by comparing all models across all datasets instead of trying to find datasets on which one or more models performed well.

**Fair and neutral setup**: At least one representative of all methods compared in this experiment was contacted, and hyperparameter configurations were discussed with all who responded. Every maintainer of the used software packages implementing the evaluated methods was given equal opportunity to influence the experiment, ensuring there was no bias in model configuration. We are grateful for the maintainers’ time supporting this effort.

**Model, performance measures, and data are chosen in a rational way**: The study is designed to assess the status quo, which excludes models and measures that have been published without peer-review. We focus on a single primary metric to evaluate discrimination and another for overall predictive performance, while additional measures are reported as a secondary reference to complement the primary analysis. We also assess models using measures, even if these are known to be partially flawed (e.g. the Harrell’s C-index exhibits increasing bias with increasing censoring percentages (see, e.g. Schmid and Potapov 2012), as this is the de facto standard and thus enables better comparisons with existing work. The inclusion criteria for datasets were as follows: We use real-world datasets that include at least two features, a right-censoring indicator with a survival time, have at least 100 observed events, and do not qualify as high-dimensional, i.e. have fewer features than observations. We exclude datasets with competing risk endpoints, recurrent events, or other non-standard settings such as left-censoring. No quota was specified regarding censoring proportions in the datasets.

**Implementation, Reproducibility, and Accessibility** Experiments were conducted on R 4.4.3 on the Beartooth Computing Environment (Beartooth Computing Environment, x86_64 cluster 2024). All code required to run the experiments and generate the results is available in a public GitHub repository (https://github.com/slds-lmu/paper_2023_survival_benchmark) licensed under GPL-3. Further details on software used are available in Supplement D. For additional reproducibility, our hyperparameter search spaces will be published with an upcoming release of *mlr3tuningspaces* (Becker 2024) and our datasets will be available as an OpenML benchmark suite (Vanschoren et al. 2014) while already being available from GitHub.

### 3.2 Models and configurations

The algorithms compared in this experiment were chosen by identifying commonly used models with readily available implementations: (1) Kaplan-Meier Estimator (KM) (Kaplan and Meier 1958); (2) Nelson-Aalen Estimator (NEL) (Aalen 1978); (3) Akritas Estimator (AK) (Akritas 1994); (4) Cox PH (CPH) (Cox 1975); (5) Additive Cox Model (GAM) (Wood et al. 2016); (6) Penalized CPH with cross-validated lambda (GLMN) (Simon et al. 2011); (7) Penalized Cox (Pen) (Goeman 2010); (8) MCP-penalized Cox Model (NCV) (Breheny and Huang 2011); (9) Parametric AFT (AFT) (Kalbfleisch and Prentice 2011); (10) Flexible Splines (Flex) (Royston and Parmar 2002); (11) Random Survival Forest (RFSRC) (Ishwaran et al. 2008); (12) Random Survival Forest (RAN) (Wright and Ziegler 2017); (13) Conditional Inference Forest (CIF) (Hothorn et al. 2006); (14) Oblique Random Survival Forest (ORSF) (Jaeger et al. 2024); (15) Relative Risk Tree (RRT) (Breiman 1998); (16) Model-Based Boosting with Cox and AFT objective (MBSTCox and MBSTAFT) (Bühlmann and Yu 2003); (17) CoxBoost (CoxB) (Binder and Schumacher 2008); (18) XGBoost with Cox and AFT objective (XGBCox and XGBAFT) (Chen and Guestrin 2016, Barnwal et al. 2022); (19) Survival-SVM (SSVM) (Van Belle et al. 2011).

The full table of all models, including respective software packages, is given in Supplement C, and the online supplement includes recorded versions of all packages involved in the experiment. All models are made accessible via mlr3 via *mlr3extralearners* (Fischer et al. 2025). In our selection, we focused on well-established models with generally robust implementations, provided as well-maintained packages or wrapper functions within benchmarking software. This excludes some recently proposed DL based methods like DeepSurv (Katzman 2016) and DeepHit (Lee et al. 2019), which have higher computational complexity, require intensive tuning, and in initial experiments could not be evaluated reliably within our benchmark suite due to a lack of stable implementations. The KM and NEL estimators are used as non-parametric baselines, while AK acts as a more flexible baseline as it estimates a conditional survival function without relying on independent or non-informative censoring. An additional table in Supplement C lists the associated hyperparameter and pre-processing configurations. Note that while some studied methods could be considered algorithmically equivalent, e.g. RAN and RFSRC both implement Random Survival Forests, their implementations are not, and for that reason, we opted to include such variants in the comparison. A small number of methods provide internal optimization methods for certain hyperparameters, which we used when available/possible in our global tuning procedure. This affects both XGBoost models and their early-stopping mechanism to tune the nrounds parameter and GLMN for lambda, while CoxB is exclusively tuned using its internal mechanism. For further details we refer to the experiment code on GitHub.

### 3.3 Resampling, tuning, prediction types, and Pre-Processing

**Resampling** is performed as nested repeated cross-validation with three outer and three inner resampling folds for honest generalization error estimates (Bischl et al. 2023). Outer cross-validation is repeated five times for datasets with a total number of events in [500,2500) and ten times for datasets with fewer than 500 events. This aids the stability of performance estimates by increasing the total number of evaluations to up to 30 (10 repetitions × 3 outer folds) for the datasets with few events, while saving computational costs for the largest tasks where the available training data is likely sufficient for reliable evaluation. Inner cross-validation is always repeated twice, leading to six total evaluations per tuning iteration. Resampling is stratified by event indicator to preserve the censoring proportion in respective folds.

**Tuning** is performed on the inner resampling folds of the nested cross-validation. We use Bayesian optimization (Garnett 2023, Bischl et al. 2023) with 50ρ iterations, where ρ is the dimensionality of the search space, ranging from 1 to 8. For example, one parameter of GLMN is tuned for 50 iterations, whereas six parameters of ORSF are tuned for 300 iterations. This method grants each method the opportunity to be tuned to the same relative amount: 50 iterations per tunable parameter. When the tuning space was finite and smaller than 50, we exhausted all configurations to achieve the same tuning result but with lower computational cost; this applied to learners AFT (tuning across three distribution families), Flex (tuning k∈{2,…,10}, and RRT (tuning minbucket∈{5,6,…,50}). We set a time limit for the tuning process of 150 hours to ensure that one outer resampling iteration (tuning and final model fitting) could be completed within seven days, a constraint imposed by the computational environment where all algorithms are run single-threaded to enable large-scale parallelization across resampling iterations; in practice, runtimes would be substantially shorter with multi-threading. This restriction was most frequently violated for memory-intensive models on datasets with many observations, where in some cases the final evaluation was unsuccessful (see Section 4 and Supplement E.5 where errors are enumerated per model and dataset). The tuning process is repeated independently for each tuning measure (see Section 3.4).

**Prediction types:** In general, there are four prediction types in survival analysis (Sonabend 2021): A linear predictor lp, continuous ranking crank (e.g. a relative risk), a distribution distr (e.g. the survival probability), and predicted survival times response. The response time is very uncommon due to its generally poor quality (Henderson et al. 2001) and only directly provided by the Survival SVM and XGBAFT at the time of writing. We focus on evaluating distribution and continuous rank predictions. The prediction types provided by individual methods (and implementations) can vary, which is why *mlr3proba* (Sonabend et al. 2021) includes so called *compositors*, which are functions to derive missing prediction types needed for evaluation when appropriate. Where models only predict a probabilistic prediction, crank is calculated as the expected mortality derived from the distr prediction (Ishwaran et al. 2008, Sonabend et al. 2022). When models predict only crank or lp, then we exclusively evaluate them on discrimination measures, which is the case for RRT, XGBAFT, MBSTAFT, and SSVM. The XGBCox predicts lp, and the distr prediction is composed using the Breslow estimator (Lin 2007) similarly to previous benchmarks (Jaeger et al. 2024).

**Pre-processing** is applied only if either technically required to run a model or in line with standard recommendations for that model class. This includes standardization of covariates to unit variance and zero mean and/or dummy encoding of categorical features. We embedded learners into pipelines with *mlr3pipelines* (Binder et al. 2021) to combine the respective pre-processing operations with the learning algorithm, which also ensures that any pre-processing parameters (e.g. scaling factors) are estimated on the respective training data only, avoiding information leakage (Hornung et al. 2015). Supplement C lists the model-specific pre-processing performed. In addition to these model-specific pre-processing steps, we collapse levels of categorical variables with frequencies below 5% as part of the model pipeline, ensuring that high-cardinality categorical features are handled consistently. As we only applied basic pre-processing, no additional hyperparameters were added to the tuning search space.

### 3.4 Performance evaluation

We assess performance using two primary measures alongside four additional measures. For cases where individual model predictions were not possible during the inner- or outer resampling procedure due to any kind of error (computational issues, non-convergence, etc.), the prediction of the KM estimator was used as a fallback result. This ensures a statistically sound evaluation, and is considered a reasonable compromise between either overpenalizing models by inserting some constant value which would depend on the given metric and dataset, or simply disregarding failed iterations during evaluation, which is overly optimistic (Fischer et al. 2024, Wünsch et al. 2025).

**Measures** chosen for this benchmark are summarized in Table 1. Of these measures, only two are used to provide primary results: Harrell’s C (Harrell et al. 1982) for pure discrimination and the Integrated Survival Brier Score (ISBS) (Graf et al. 1999) for overall predictive ability, including calibration. The benchmark procedure is run twice, tuning either for discrimination (Harrell’s C) or overall predictive ability (ISBS), and evaluated on the corresponding family of measures: Harrell’s C alongside the related Uno’s C (Uno et al. 2011) for discrimination, and ISBS alongside scoring rules for overall predictive ability. For integrated measures, we use the commonly chosen 80% quantile as the upper bound (Sonabend et al. 2025). We additionally explore the calibration measures D-Calibration (Haider et al. 2020) and van Houwelingen’s α (van Houwelingen 2000), which we only apply on models tuned with ISBS.

**Statistical Analysis** is conducted following Demšar (Demšar 2006), initially performing global Friedman rank sum tests for all measures, where the “groups” are the models and the “blocks” are the independent datasets. Significance after Bonferroni-Holm adjustment determines whether post-hoc tests are conducted. Post-hoc Bonferroni-Dunn tests are conducted and presented as critical difference diagrams, using CPH as the reference model for comparison.

As an exploratory post-hoc sensitivity analysis, we use the Plackett-Luce (PL) model (Luce 1959, Plackett 1975, Turner et al. 2020) for rank-analysis, which estimates “(log-)worth” parameters for each survival model and offers quasi-standard errors (Firth 2004). In the context of the PL model, each dataset is a “rater,” ranking each “item” (survival model), and higher estimated worth indicates higher preference for the learner in the overall ranking. We fit PL models both on the full set of rankings for each tuning measure, as well as for dataset subgroups based on pre-defined dichotomization criteria: **(1)** The global Grambsch-Therneau test (Grambsch and Therneau 1994) is significant at the 5% level, which we use as a proxy for misspecification of the CPH model (Keele 2010, Wynant and Abrahamowicz 2014, Metzger 2024), i.e. non-linear effects, interactions or violation of the proportional hazards (PH) assumption; **(2)** The censoring proportion, using the median (45.3%) as cut-off point; **(3)** The sample size to feature ratio $\frac{n}{p}$, as a proxy for dimensionality, using the median (189) as cut-off point; **(4)** The sample size only, dividing datasets at N=1000. We compare each set of subgroup-specific PL models using likelihood ratio test (LRT) to assess statistically significant difference between the subgroup rankings implied by the PL models. As a data-driven alternative, we use PL trees, which use model-based recursive partitioning (Zeileis et al. 2008) and extend Bradley-Terry trees (see Strobl et al. 2011) to automatically detect statistically significant differences in subgroups of datasets based on meta-information using similar features as used in the manual subgroup analysis ($\text{log}\;\frac{n}{p}$, censoring proportion, and CPH misspecification).

### 3.5 Datasets

To obtain a collection of suitable datasets, we ran a search across the CRAN Task View “Survival Analysis” (https://cran.r-project.org/view=Survival), Python’s *pycox* library and related literature (Section 2) and existing collection of survival datasets (Drysdale 2022), yielding over 120 datasets. After applying the dataset inclusion criteria (Section 3.1) and removing duplicates and derivations of other datasets, a total of 34 datasets remained. Minor changes were made to variable names, recoding of factor levels, and deletion of non-informative or “illegal” covariates like ID numbers. Observations were deleted if their event time is equal to zero. Since this benchmark is not concerned with a model’s ability to handle or impute missing covariate data, observations with missing values were removed, which occurred very rarely. Lastly, in cases where datasets had a large number of unique time points, the time variable was coarsened by appropriate rounding, greatly reducing computational cost for some methods, including RAN, AK, and CIF. For full details, see the pre-processing code contained in the GitHub repository (Section 3.1). Summaries of the datasets in terms of the number of observations and covariates after modification and censoring proportions, along with citations for the respective sources, can be found in Supplement B.

## 4 Results

Global Friedman tests were significant for all measures, indicating the presence of significant differences between models and allowing for post-hoc analysis. The number of times models failed to compute results due to either time or memory constraints and required the score imputation using KM (Section 3.3) is tabulated in Supplement E.5. We present critical difference (CD) plots for the baseline-comparison to CPH for both discrimination and overall performance [see Section 3.4, Demšar (2006)]. The top line of a CD plot represents a model’s average performance rank across all datasets in the benchmark, where a lower ranking implies better performance regardless of the evaluation measure applied. Thick horizontal lines around the CPH model rank indicate the symmetric CD, meaning that other models within this range do not statistically significantly differ in rank from the reference model.

### 4.1 Discrimination

**Figure 1:**
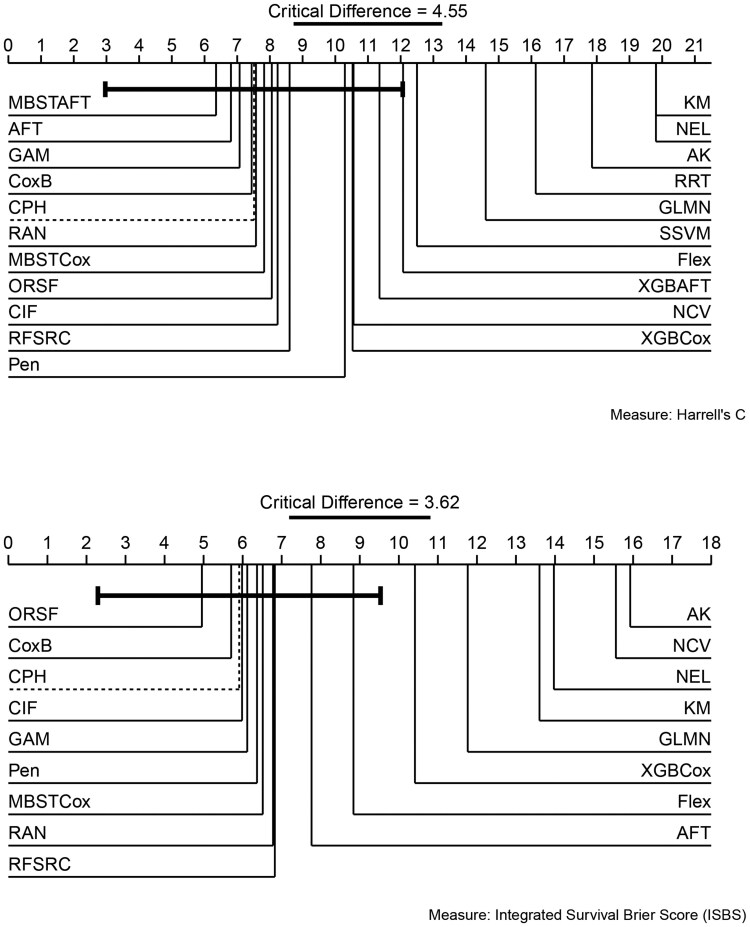
Critical difference plots comparing models with the CPH reference tuned and evaluated on either Harrell’s C (top) or ISBS (bottom). Superior models (lower ranking scores) are on the left with decreasing performance (higher rank) moving right. Models connected by thick horizontal lines do not perform significantly differently from the baseline when adjusting for multiple comparisons.

CD plots for discrimination, tuned and evaluated on Harrell’s C (Fig. 1, top), indicate that all models outperform the baseline learners (KM, NEL, AK) as expected. MBSTAFT, AFT, GAM, and CoxB are the top-performing models but fail to significantly outperform the CPH baseline. The remaining models mostly belong to the classes of RSFs and GBMs, which all achieve average ranks between 6.5 and 8, indicating similar discrimination performance. Pen, XGBCox, NCV, XGBAFT, and Flex fall slightly behind, but still lie within the CD.

### 4.2 Overall performance

The CD plot for overall performance tuned and evaluated on ISBS (Fig. 1, bottom) similarly indicates that no model significantly outperforms CPH. Only ORSF and CoxB perform better, while CIF ranks almost identically to CPH. Pen, MBSTCox, RAN, and RFSRC fall slightly behind, with AFT and Flex further behind. XGBCox, GLMN, and NCV are the only non-baseline models significantly outperformed by CPH.

**Figure 2:**
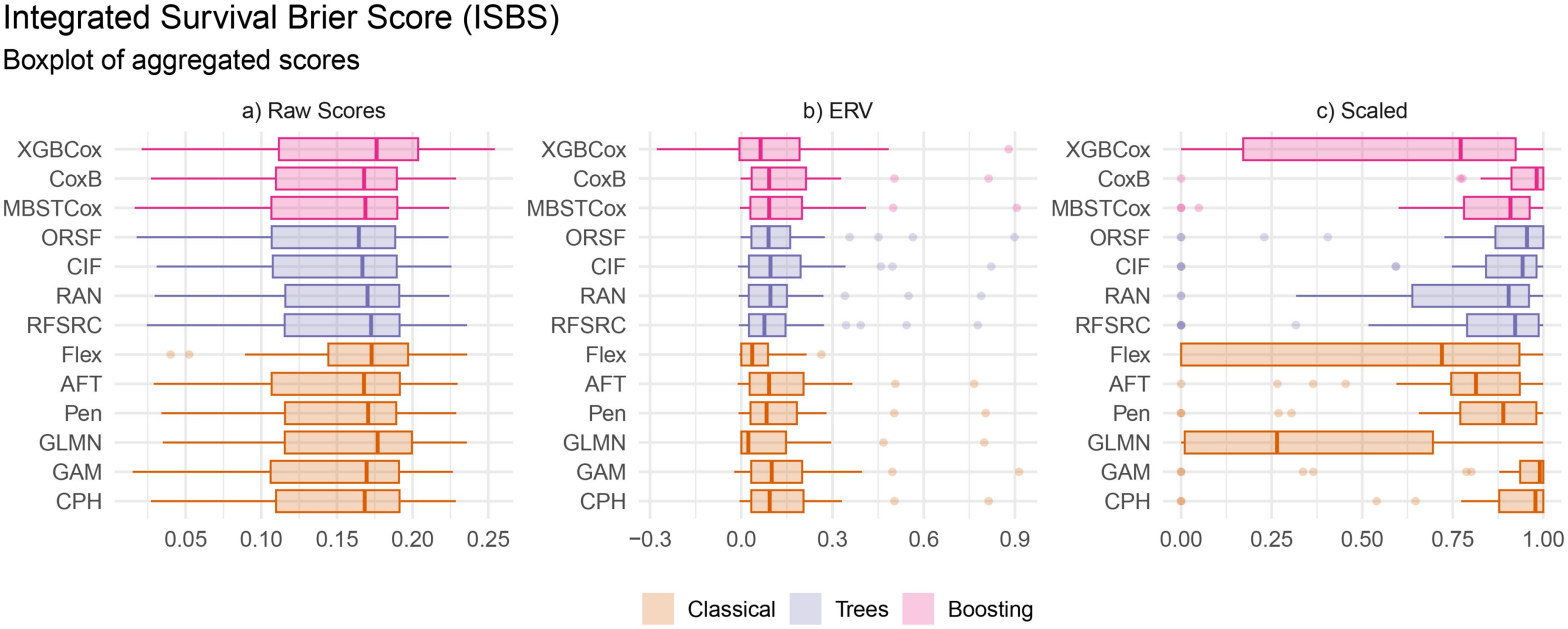
Boxplots of aggregated scores across all datasets for non-baseline models tuned and evaluated with ISBS showing unmodified ISBS scores (a), Explained Residual Variation (ERV) scores (b), and scores scaled such that 0 is equivalent to KM and 1 is achieved by the best model for each dataset and measure. AK and NCV are omitted due to largely off-the-scale negative ERV values to avoid scaling issues.

We additionally present boxplots both for individual scores per dataset and aggregated scores. We offer three versions of these aggregated scores to support evaluation and analysis, illustrated by Fig. 2: a) Raw scores as calculated by the corresponding measure; b) “Explained Residual Variation” (ERV) (Korn and Simon 1991) scores similar to the “Index of Prediction Accuracy” (Kattan and Gerds 2018) where negative values imply performance worse than KM, 0 is equivalent to KM, and 1 denotes a perfect model; c) Scaled scores, whose interpretation is the same as the ERV ones, with the difference that 1 is achieved by the best model for a given task and measure (Caruana and Niculescu-Mizil 2006).

### 4.3 Calibration

Calibration was assessed using D-Calibration and van Houwelingen’s α (see Table 1), with all models tuned for ISBS. Results for these two measures are mildly contradictory, with some models (RFSRC, RAN, ORSF) appearing well-calibrated by one measure but not the other. We note that both measures should be considered experimental as they are not yet well-evaluated, and full results including visualizations are presented in Supplement E.3. Further results for all measures are in line with the findings presented here and can be found in Supplement E, including violin plot versions of all boxplots presented here. Additional tables and visualizations are available on our results website which is linked in our GitHub repository. The repository also provides downloads for all raw results, including tuning archives for the close to 20,000 individual tuning evaluations performed in this experiment.

### 4.4 Sensitivity analysis

The full PL model’s result is consistent with our primary analysis: No model is statistically superior to CPH across all datasets. In the manual subgroup analysis, LRTs indicate that CPH misspecification status significantly affects model rankings for both measures (*P* < .01), while censoring proportion and $\frac{n}{p}$ ratio only significantly affect rankings when evaluated on ISBS (*P* < .01 and *P* < .05, respectively) but not Harrell’s C. When the CPH model is misspecified, CPH loses its leading position: for Harrell’s C, MBSTAFT and MBSTCox rank best, while for ISBS, CIF and ORSF are superior. Censoring proportion largely affects ORSF and CIF, which rank considerably better on low-censoring datasets. The $\frac{n}{p}$ ratio has little practical effect on model rankings. The sample size subgroup analysis (N<1000 vs. N≥1000) is significant for both measures (*P* < .001): on larger datasets, models with larger hypothesis spaces such as MBSTAFT and MBSTCox rank higher, while CPH and CoxB lead on smaller datasets. PL trees were run on two configurations: strict (α=0.1, minsize = 10), where a split on CPH misspecification was found for ISBS-tuned models, and lenient (α=0.2, minsize = 5), where splits on CPH misspecification were found for both Harrell’s C and ISBS. No other variables were used for splitting. Full results including worth parameter plots for all subgroups and PL tree visualizations are in Supplement E.4.

## 5 Discussion

**Discrimination and Overall Performance** Our main finding across both evaluation measures is that no model statistically significantly outperforms CPH in aggregate, despite CPH rarely ranking in first place on any dataset. For discrimination, the top-performing models include both classical (AFT, CPH) and ML methods (MBSTAFT, RAN, CoxB), each falling within the CD of CPH. For overall performance as measured by ISBS, results are similar: ORSF and CoxB rank slightly above CPH, but remain within the CD as well. Several ML methods that perform on par with CPH for discrimination rank noticeably lower on ISBS, suggesting that their calibration may be lacking, which is an important aspect of distributional prediction. CoxB and ORSF are exceptions, performing consistently well across both settings. Conversely, AFT-based approaches rank well for discrimination but fall behind Cox-based methods on ISBS, indicating that their distributional predictions may be less reliable. Among the penalized Cox variants, Pen consistently outperforms GLMN despite their similarity; the main difference is that GLMN performs internal cross-validation for its primary regularization parameter, which in this low-dimensional setting could be a disadvantage. Analogously, both GBMs with Cox objective (MBSTCox, XGBCox) rank differently, with XGBCox’s poor showing partly attributable to computational errors requiring imputation (Supplement E.5). CoxB did not require any explicit tuning outside of its internal optimization, making it computationally more efficient while achieving comparable or better performance. We also note that our use of the parametric AFT model can be considered unconventional, as we tune the distribution family (i.e. whether to use a Weibull, log-normal, or log-logistic distribution) within the tuning iterations, thus performance evaluation in the outer resampling iterations could be based on a different distribution. This also affects XGBAFT analogously. In real-world scenarios, a specific distribution is typically chosen in advance. Since not all methods could be evaluated on both metrics (Section 3.3), the CD in each comparison are affected by the differing number of methods, which makes a direct comparison difficult.

**Calibration** The two calibration measures employed yield partially contradictory results, with some models (RFSRC, RAN, ORSF) appearing well-calibrated by one measure but not the other. This discrepancy highlights the current lack of well-established calibration measures for survival models and suggests that calibration assessment remains an open challenge requiring further methodological research (see e.g. Austin et al. 2020). Models with poor calibration by both measures, notably AK and XGBCox, also rank poorly on ISBS, which reaffirms that ISBS captures both discrimination and calibration. Full calibration results including visualizations are presented in Supplement E.3. Generally speaking, multiple measures should be considered for performance evaluation, as they may highlight aspects of performance relevant in different contexts. Results on individual datasets are presented in Supplement F, where the overall trend is similar to the aggregated results with expected variation between individual tasks.

**Sensitivity Analysis** The sensitivity analysis qualifies the primary finding: the aggregate result that no model statistically significantly outperforms CPH may mask meaningful heterogeneity across characteristics of the datasets and data-generating processes. Analysis of datasets for which the Grambsch-Therneau test is significant (indicating presence of non-linear effects, interactions, or violation of the PH assumption) suggests that more flexible methods offer a genuine advantage in some cases. This result should be interpreted carefully, however: CPH not performing well on datasets where CPH assumptions are violated is partly a tautology, although other models with linear predictors (CoxB, AFT) show similar patterns. It does however validate the common practice of benchmarking multiple models with varying degrees of flexibility on a specific dataset in order to assess best fit. The sample size result further indicates that more complex data-generating processes may also require a sufficiently large dataset to provide enough power for their estimation. Importantly, these two findings are not independent: the power of the Grambsch-Therneau test itself increases with sample size, so datasets flagged as misspecified tend to also be larger. Finally, censoring proportion affects distributional predictions more than discrimination, potentially pointing to challenges in tail estimation under heavy censoring. The $\frac{n}{p}$ ratio has negligible practical impact, consistent with the low-dimensional nature of the benchmark, where the available datasets may have insufficient range to reveal such effects.

### 5.1 Limitations

Our use of the AFT model prioritizes prediction over interpretation and could be simplified by splitting the tuned model into one model for each functional form (“Weibull,” “log-normal,” “log-logistic”), which would yield more interpretable results. Since our focus lies on generalizability to low-dimensional, right-censored settings, our results will not generalize to more complex settings. However, extension to left-censoring, competing risks, or other more complex endpoints first necessitates more comprehensive support by models and their implementations, as well as adaptation of available evaluation measures to these scenarios. Similar limitations apply to the datasets with hierarchical data structures (e.g. multi-centre studies) or time-dependent covariates, both of which may affect relative model performance but require specialized modelling strategies beyond the scope of the current study but are interesting targets for future research. The number of datasets included in the study exceeds that of the vast majority of previous benchmarks, but could and should still be extended in order to increase power for post-hoc analyses and reliability of overall results.

### 5.2 Conclusions and future work

Our results demonstrate that in aggregate, no ML method outperforms CPH across low-dimensional, right-censored survival tasks with statistical significance. However, the sensitivity analyses suggest that tree-based approaches like CIF, ORSF, and boosting methods like MBSTAFT and MBSTCox, offer a meaningful advantage when the data-generating process contains non-linear effects, interactions, or violation of the PH assumption (as assessed by the Grambsch-Therneau test). We therefore recommend that practitioners start with CPH (or CoxB) as strong, efficient baselines, and consider other ML alternatives primarily when there is evidence of CPH misspecification or when distributional predictions under low censoring are required. While it is possible to achieve better predictive accuracy in individual cases (see Supplement F), the additional computational cost and loss of interpretability may not be justified in many cases. Expanding this benchmark with a wider range of settings would be beneficial, particularly regarding high-dimensional datasets and more complex endpoints, pending the corresponding software support and data availability.

## Supplementary Material

btag186_Supplementary_Data

## Data Availability

The data underlying this article are available in the online supplement at https://github.com/slds-lmu/paper_2023_survival_benchmark, which is also archived on Zenodo at https://doi.org/10.5281/zenodo.19075310.
