# Supplementary material for: A large-scale neutral comparison study of survival models on low-dimensional data
Source: Bioinformatics. 2026 Apr 16;42(5):btag186. doi: 10.1093/bioinformatics/btag186 (PMC13148964; doi:10.1093/bioinformatics/btag186)
Supplement: btag186_Supplementary_Data [file btag186_supplementary_data.pdf]

Supplementary Material:
A Large-Scale Neutral Comparison Study of Survival Models on Low-Dimensional Data

Lukas Burk, John Zobolas, Bernd Bischl, Andreas Bender, Marvin N. Wright, Raphael Sonabend

A Literature Review

The following represents an extended literature review complementary to the summary provided in the main article.

Comparisons of ML and Classical Models The experiments carried out in this paper fall into this category. Only two prior experiments could be found that neutrally benchmarked more than one ML model class on low-dimensional data. Kattan [2003] benchmarked tree-based models, ANNs and CPH with Harrell's C-index across three datasets with varying censoring proportions. The models are compared for significant differences by repeating the experiments up to 50 times with different seeds thus allowing for different hyperparameter configurations and folds in cross-validation. Boxplots across all replications indicate that no machine learning model outperformed the CPH. Zhang et al. [2021] compare classical and ML methods, taking into account feasibility and computational efficiency for various tasks in the biomedical field. Methods are evaluated on six clinical and 16 omics datasets using 11 metrics, including time-dependent AUC, Brier score and multiple variations of the C-index. However, methods were applied with specific hyperparameter sets without tuning, thereby limiting the generalizability of their results.

Comparisons on High-Dimensional Data Herrmann et al. [2021] performed a large-scale benchmark experiment of survival models on multi-omics high-dimensional data. Models fall into the following groups: Penalized regression, GBMs, and RSFs. Comparisons are made with Uno's C and the Integrated Survival Brier Score (ISBS). The ISBS for all models overlapped with the Kaplan-Meier baseline though all C-indices were significantly higher than the baseline. It is not stated in the paper if multiple testing correction is applied. The authors also note that their results should be treated with caution due to the small performance differences and high variability. Spooner et al. [2020] also compared machine learning models on high-dimensional data. In this study GBMs, RSFs, CPH and some extensions thereof were compared. Models were evaluated by Harrell's C-index only. The results indicated that all models outperformed CPH when no additional feature selection was used but that there were no significant differences when feature selection was applied to the Cox model. There were few significant statistical differences between models. Wissel et al. [2023] provide a systematic comparison of multi-omics cancer survival models comparing eight DL methods, RSFs and CPH. They primarily focus on the noise-resistance of these models for high-dimensional settings, finding a general lack thereof when evaluating on Antolini's C-index and ISBS.

Comparisons of Classical Models Moghimi-Dehkordi et al. [2008] compare CPH to AFT models with various distributions. Out-of-sample measures for comparison are not provided though the AIC produced by the CPH is far higher (and therefore inferior) than those of the parametric models. Model inspection demonstrated that all models provided similar (non-significantly different) confidence intervals for hazard ratios. Georgousopoulou et al. [2015] compared the CPH to a Weibull and Exponential AFT model. Again no out-of-sample measures were utilized, models were compared by the Cox-Snell residuals and the Bayesian Information Criterion (BIC). Similarly to Moghimi-dehkordi *et al.*, hazard ratios produced from all three models were nearly identical. The authors claim the CPH is inferior to the parametric models though only graphical comparisons are included. Zare et al. [2015] provide another comparison of the CPH to AFT models, using Cox-Snell residuals and AIC as their measures of comparison. Similarly to the previous studies the Cox model has the highest AIC though the plotted Cox-Snell residuals are very similar. The authors acknowledge no significant differences between the model classes and instead conclude that AFT is a useful and more interpretable alternative. No significant differences were found between the different AFT parameterizations. Dirick et al. [2017] make use of a financial setting to compare the CPH, AFT, flexible Cox models using splines to model the hazard, and mixture cure models. The authors compare the models using a time-dependent AUC, the mean squared error (MSE), and the mean absolute error (MAE). Survival times are generated from the CPH with a deterministic composition using quantiles chosen to minimize the MSE and MAE, and it is not clear if this is performed in an unbiased nested resampling manner or after predictions are made. By averaging the ranking of model performance the authors conclude that CPH with penalized splines outperformed the other models with respect to the chosen metrics.

Habibi et al. [2018] performed another experiment on PH and AFT models. Models were again compared exclusively by the AIC with the PH having the highest result and log-normal AFT the lowest; differences between AFT models were non-significant. Confidence intervals for hazard ratios were similar (non-significantly different) for all models.

Comparisons of a Novel Model Class Luxhoj and Shyur [1997] benchmarked neural networks against CPH in the engineering field of reliability analysis. The baseline hazard of the Cox model is modeled by splines with a single knot. The models are compared using the mean squared error on a validation set of sample size 40, of these 40 there are only 9 unique failure times that are used for model testing. Insufficient information is provided to determine the architecture or training procedure of the neural networks compared. The MSE difference between the Cox and ANN was 0.003, which is highly unlikely to be a significant difference on a test set of only 40 observations with nine observed events. Ohno-Machado [1997] also compared CPH to ANNs. Several Cox models were fit with automated variable selection by backwards elimination. For each model, survival curves were predicted and for a given patient they were considered dead at a particular time point if the predicted survival curve at the time is less than the "arbitrary" Ohno-Machado [1997] probability of 0.5. The Cox models were compared to a single hidden layer ANN. This model made probabilistic predictions of death in four time-intervals that were within the predicted time of the Cox models. The probabilistic predictions from both models were compared with the AUC and its corresponding ROC. No significant differences in performance were found between the two models. Goli et al. [2016] provide a comprehensive comparison of support vector machine models with CPH as a reference class (Kaplan-Meier is not included). Models are compared against the C-index and log-rank test, though it is unstated which C-index is utilized. No model outperformed CPH with respect to the chosen C-index. Jaeger et al. [2024] compare multiple variations of a novel implementation of oblique RSFs ("aorsf") to the previous implementation, as well as other

RSFs, GBMs, penalized CPH, and ANNs. They compare methods on 21 datasets, including low- and high-dimensional settings. Results are analyzed using post-hoc Bayesian ROPE and evaluated using the Index of Prediction Accuracy (IPA) [Kattan and Gerds, 2018] based on the ISBS and time-dependent C-index [Blanche et al., 2013]. They present results relative to the method performing best in their benchmark ("aorsf-fast" for both measures), with GBMs among the lowest-performing methods. Only minimal tuning was conducted.

A.1 Surveys of Survival Models

The final class of papers do not perform empirical benchmark experiments but instead survey/review available survival models. These are therefore only discussed very briefly. Ohno-Machado [2001] provide an overview of models available for survival analysis from non-parametric estimators and classical models to neural networks. The review highlights useful applications of the models and their respective limitations. In particular their Table 1 clearly states advantages and disadvantages of Cox models versus ANNs. Patel et al. [2006] compare proportional hazards and accelerated failure time models. This comparison is primarily theoretical and based on model properties, no analytical comparison with measures is provided though comparisons of predicted median survival times are compared to those from a Kaplan-Meier estimator. The authors conclude that AFT models should be considered more often due to simpler interpretation. Wang et al. [2019] provide a review of survival analysis models and measures that is strongly recommended here as a precise and comprehensive introduction to the field of machine learning in survival analysis. The authors provide strong arguments for comparing classical models against one another, though this is not extended to the machine learning setting. More mathematical detail is provided for the classical setting; however, a clear and detailed overview is still provided for machine learning models. Some attention is also given to the more complex cases of competing risks and multiple events. Lee and Lim [2019] provide a short but concise overview of survival analysis models with an emphasis on genetic data and implementation in R. Their review covers classical models, penalization, and many machine learning models. They provide a clear, practical illustration (but no full benchmark experiment) comparing the models on a real dataset against Harrell's C. No model outperforms CPH. Wiegrebe et al. [2024] provide a comprehensive overview of deep learning methods for survival analysis and compare methods based on their capabilities regarding various common challenges in survival analysis such as time-varying features, competing events, different censoring types, dimensionality, modality, and interpretability. Their extensive comparison is also available as a web-based interactive table.

B Datasets

Table 1 lists datasets included in the benchmark along with their sources and common descriptive statistics. Section B lists the licenses declared by the source packages from which the dataset is taken.

Table 1: Datasets used in benchmark experiment. Columns: (1) Dataset name and citation; (2) OpenML dataset ID, retrievable via `mlr3oml::odt(id)`; (3) Censoring proportion (%); (4–5) Number of continuous and discrete features before recoding; (6–7) Number of observations and features after preprocessing; (8) Number of observed events; (9) Source package.

**Dataset**	**OpenML ID**	**Cens %**	n_C	n_D	**n**	**p**	n_E	**Package**
aids.id [Carlin and Louis, 2018]	46130	60	1	4	467	5	188	*JM* [Rizopoulos, 2010]
aids2 [Venables and Ripley, 2002]	46158	38	1	3	2814	4	1733	*MASS* [Venables and Ripley, 2002]
bladder0 [Sylvester et al., 2006]	46172	48	0	3	397	3	206	*frailtyHL* [Ha et al., 2019]
CarpenterFdaData [Carpenter, 2002]	46159	36	15	11	408	26	262	*simPH*
cat_adoption [Kuhn, 2024]	47176	37	14	4	2257	18	1434	*modeldata* [Kuhn, 2024]
channing [Klein and Moeschberger, 2003]	46154	62	1	1	458	2	176	*KMsurv*
check_times [Kuhn, 2024]	47177	1	22	0	13626	22	13523	*modeldata*
child [Broström, 2021]	46147	79	1	3	26574	4	5616	*eha* [Broström, 2021]
colrec [M. Pohar and J. Stare, 2006]	46145	17	3	2	5578	13	4602	*relsurv* [M. Pohar and J. Stare, 2006]
cost [Jørgensen et al., 1996]	46148	22	3	10	518	13	404	*pec* [Mogensen et al., 2014]
dataFTR [Trébern-Launay et al., 2013]	46149	86	0	2	2206	2	300	*RISCA* [Foucher et al., 2023]
dataSTR [Trébern-Launay et al., 2013]	46150	82	0	4	546	4	101	*RISCA*
e1684 [Kirkwood et al., 1996]	46157	31	1	2	284	3	196	*smcure* [Cai et al., 2012]
flchain [Dispenzieri et al., 2012]	46161	72	4	3	7871	7	1082	*survival*
gbsg [Katzman et al., 2018]	46131	43	3	4	2232	7	1267	*pycox* [Kvamme, 2018]
grace [Hosmer Jr et al., 2011]	46168	68	4	2	1000	6	324	*mlr3proba* [Sonabend et al., 2021]
hdfail [Monaco et al., 2018]	46167	94	1	4	52422	5	2885	*frailtySurv* [Monaco et al., 2018]
kidtran [Klein and Moeschberger, 2003]	46155	84	1	3	863	4	140	*KMsurv*
liver [Andersen et al., 1993]	46160	40	1	1	488	2	292	*joineR* [Williamson et al., 2008]
lung [Loprinzi et al., 1994]	46162	28	5	3	167	8	120	*survival*
metabric [Katzman et al., 2018]	46142	42	5	4	1903	9	1103	*pycox*
mgus [Kyle, 1993]	46163	6	6	1	176	7	165	*survival*
nafld1 [Allen et al., 2018]	46164	92	4	1	12446	5	1018	*survival*
nwtco [Breslow and Chatterjee, 1999]	46165	86	1	2	4028	3	571	*survival*
ova [Van Houwelingen et al., 1989]	46151	26	1	4	358	5	266	*dynpred*
rdata [M. Pohar and J. Stare, 2006]	46146	47	1	3	1040	4	547	*relsurv* [M. Pohar and J. Stare, 2006]
std [Klein and Moeschberger, 2003]	46156	60	3	18	877	21	347	*KMsurv*
support [Katzman et al., 2018]	46144	32	10	4	8873	14	2705	*pycox*
tumor [Bender and Scheipl, 2018]	46171	52	1	6	776	7	375	*pammtools*
uis [Hosmer et al., 2008]	46153	19	7	5	575	12	464	*quantreg* [Koenker, 2021]
veteran [Kalbfleisch and Prentice, 2011]	46166	7	3	3	137	6	128	*survival*
wa_churn [Kuhn, 2024]	47178	73	8	10	7032	18	1869	*modeldata*
wbc1 [The Benelux C M L Study Group, 1998]	46152	43	2	0	190	4	109	*dynpred*
whas [Hosmer Jr et al., 2011]	46169	48	3	6	481	9	249	*mlr3proba*

Table 2: Software licenses of the R and Python packages used as sources for datasets in this benchmark.

License	Packages
BSD-2	pycox
GPL	relsurv
GPL (≥ 2)	jm, eha, pec, RISCA, dynpred, quantreg
GPL (≥ 3)	KMsurv
GPL-2	smcure
GPL-2 $\|$ GPL-3	nnet
GPL-3	simPH, joineR
LGPL (≥ 2)	survival
LGPL-2	frailtySurv
LGPL-3	mlr3proba
MIT	modeldata, pammtools
Unlimited	frailtyHL

C Models and Configurations

Table 3 lists all the compared algorithms, along with their R package name, and prediction types. The horizontal lines separate the models into different groups: (1) Baseline learners (2) Classical parametric models including penalized versions (3) Tree-based methods, including random survival forests and Trees (4) Boosting algorithms including gradient- and likelihood-boosting (5) Support Vector Machines (SVMs). Table 5 shows hyperparameter search spaces and non-default parameter values as well as common pre-processing requirements.

Table 3: Models used for benchmarking with associated packages and prediction types.

Model Information			Prediction Types		
**Model Name**[1]	**Learner**[2]	**Package**[3]	**distr**[4]	**crank**[5]	**lp**[6]
Kaplan-Meier (KM) [Kaplan and Meier, 1958]	kaplan	survival	✓	ExpMort	✗
Nelson-Aalen (NEL) [Aalen, 1978]	nelson	survival	✓	ExpMort	✗
Akritas Estimator (AK) [Akritas, 1994]	akritas	survivalmodels	✓	ExpMort	✗
Cox PH (CPH) [Cox, 1972]	coxph	survival	✓ (Breslow)	lp	✓
Additive Cox Model (GAM) [Wood et al., 2016]	gam	mgcv	✓	lp	✓
Penalized CPH with CV (GLMN) [Simon et al., 2011]	cv_glmnet	glmnet	✓ (Breslow)	lp	✓
Penalized Cox (Pen) [Goeman, 2010]	penalized	penalized	✓ (Breslow)	ExpMort	✗
MCP-penalized Cox (NCV)	cv_ncv	surv.cv_ncvsurv	✓	lp	✓
Parametric (AFT) [Kalbfleisch and Prentice, 2011]	parametric	survival	AFT	lp	✓
Flexible Splines (Flex) [Royston and Parmar, 2002]	flexible	flexsurv	✓	lp	✓
Random Survival Forest (RFSRC) [Ishwaran et al., 2008]	rfsrc	randomForestSRC	✓	ExpMort	✗
Random Survival Forest (RAN) [Ishwaran et al., 2008, Wright and Ziegler, 2017]	ranger	ranger	✓	ExpMort	✗
Conditional Inference Forest (CIF) [Hothorn et al., 2006]	cforest	partykit	✓	ExpMort	✗
Oblique Random Survival Forest (ORSF) [Jaeger et al., 2024]	orsf	aorsf	✓	ExpMort	✗
Relative Risk Tree (RRT) [Breiman, 1998]	rpart	rpart	✗	✓	✗
Model-Based Boosting (MBSTCox) [Bühlmann and Yu, 2003]	mboost	mboost	✓ (Breslow)	lp	✓
Model-Based Boosting (MBSTAFT) [Bühlmann and Yu, 2003]	mboost	mboost	✗	lp	✓
CoxBoost (CoxB) [Binder and Schumacher, 2008]	cv_coxboost	CoxBoost	✓ (Breslow)	lp	✓
XGBoost (XGBCox) [Chen and Guestrin, 2016]	xgboost	xgboost	Breslow	lp	✓
XGBoost (XGBAFT) [Barnwal et al., 2022]	xgboost	xgboost	✗	lp	✓
SSVM-Hybrid (SSVM) [Van Belle et al., 2011]	svm	survivalsvm		✓	✗

1. Identifier for the algorithm. Model abbreviations in parentheses are used in results.
2. Learner ID in *mlr3*.
3. Package in which the learner is implemented. Most learners are available in *mlr3extralearners*, with KM, RRT, and CPH provided via *mlr3proba*, where existing R implementations are wrapped into the *mlr3* framework. AK is natively implemented in *survivalmodels*.
4. `distr` predict type in *mlr3proba* is the probabilistic prediction. A check (✓) indicates that the distribution is provided directly by the package, sometimes via the Breslow estimator (in parentheses). Parametric (AFT) models construct the distribution internally in *survivalmodels*, while XGBCox relies on *mlr3proba* 's `breslow()` function since *xgboost* does not estimate distributions by default. A cross (✗) means the prediction is not available.
5. `crank` predict type in *mlr3proba* is the continuous ranking prediction. A check (✓) indicates that the ranking is being predicted directly by the package. 'ExpMort' stands for expected mortality and is a risk score composed from the predicted survival distribution (`distr`). 'lp' represents the ranking being

identical to the predicted linear predictor.
6. `lp` predict type in *mlr3proba* is the linear predictor prediction. (✓) indicates the linear predictor being provided by the package whereas a cross (✗) means the prediction is not available (and cannot be composed).

Table 4: Model properties to aid interpretation of results. NL: non-linear covariate effects; IA: covariate interactions; NPH: does not assume proportional hazards. ✓ yes, ✕ no, – n/a. [a]With `btree` base learner (depth-1 stumps): non-linear but additive (no interactions).

**Model**	**NL**	**IA**	**NPH**
KM: Kaplan-Meier	–	–	✓
NEL: Nelson-Aalen	–	–	✓
AK: Akritas	✓	✓	✓
CPH: Cox PH	✕	✕	✕
GAM: Additive Cox	✓	✕	✕
GLMN: Penalized CPH	✕	✕	✕
Pen: Penalized Cox	✕	✕	✕
NCV: MCP-penalized Cox	✕	✕	✕
AFT: Parametric AFT	✕	✕	✓
Flex: Flexible Splines	✕	✕	✕
RFSRC: Random Survival Forest	✓	✓	✓
RAN: Random Survival Forest	✓	✓	✓
CIF: Conditional Inference Forest	✓	✓	✓
ORSF: Oblique RSF	✓	✓	✓
RRT: Relative Risk Tree	✓	✓	✓
MBSTCox: Boosting, Cox obj.	✓[a]	✕	✕
MBSTAFT: Boosting, AFT obj.	✓[a]	✕	✓
CoxB: CoxBoost	✕	✕	✕
XGBCox: XGBoost, Cox obj.	✓	✓	✕
XGBAFT: XGBoost, AFT obj.	✓	✓	✓
SSVM: Survival SVM	✕	✕	–

Table 5: Hyper-parameter search-spaces for tuning and non-default configurations for models.

**Model**	**Hyper-parameters**[1]	**Values**[2]	**Standardize**[3]	**Encode**[4]
KM	-	-	✗	✗
NEL	-	-	✗	✗
AK	lambda	$[0, 1]$	✗	✗
CPH	-	-	✗	✗
GLMN	alpha	$[0, 1]$	✗	✓
Pen	lambda1	$2^{[-10,10]}$	✗	✗
	lambda2	$2^{[-10,10]}$		
NCV	penalty	MCP	✗	✓
	alpha	1		
	gamma	$\exp[0, \ln(10)]$		
AFT	dist	{weibull, lognormal, loglogistic}	✗	✓
Flex	k	$\{1, ..., 10\}$	✗	✗
RFSRC	splitrule	{logrank, bs.gradient}	✗	✗
	ntree	1000		
	mtry	$\{1, ..., p\}$		
	nodesize	$\{1, ..., 50\}$		
	samptype	{swr, swor}		
	sampsize	$[0, 1]$		
RAN	splitrule	{logrank,C,maxstat}	✗	✗
	num.trees	1000		
	mtry	$\{1, ..., p\}$		
	min.node.size	$\{1, ..., 50\}$		
	replace	{TRUE, FALSE}		
	fraction	$[0, 1]$		

Continued on next page...

Table 5: (continued)

Model	Hyper-parameters[1]	Values[2]	Standardize[3]	Encode[4]
CIF	ntree	1000	×	×
	mtry	$\{1, ..., p\}$		
	minsplit	{1,...,50}		
	mincriterion	$[0, 1]$		
	replace	{TRUE, FALSE}		
	fraction	$[0, 1]$		
ORSF	control_type	fast	×	×
	n_tree	1000		
	mtry	$\{1, ..., p\}$		
	leaf_min_events	{5,...,50}		
	min_obs_to_split_node	min_events_to_split_node + 5		
	alpha	$(0, 1)$		
RRT	minbucket	$\{5, ..., 50\}$	×	×
MBSTCox	family	coxph	×	×
	mstop	$\{10, ..., 5000\}$		
	nu	$(0, 0.1]$		
	baselearner	{bols, btree}		
MBSTAFT	family	{gehan, weibull}	×	×
	mstop	$\{10, ..., 5000\}$		
	nu	$(0, 0.1]$		
	baselearner	{bols, btree}		
CoxB	penalty	optimCoxBoostPenalty	×	✓
	maxstepno	5000		
	K	3		

Continued on next page...

Table 5: (continued)

Model	Hyper-parameters[1]	Values[2]	Standardize[3]	Encode[4]
	objective	survival:cox		
	tree_method	hist		
	booster	gbtree		
	max_depth	{1,...,20}		
XGBCox	subsample	$[0, 1]$	✗	✓
	colsample_bytree	$(0, 1]$		
	early_stopping_rounds	50		
	eta	$[10^{-5}, 10^{5}]$		
	grow_policy	{depthwise, lossguide}		
	objective	survival:aft		
	tree_method	hist		
	booster	gbtree		
	max_depth	{1,...,20}		
	subsample	$[0, 1]$		
XGBAFT	colsample_bytree	$(0, 1]$	✗	✓
	early_stopping_rounds	50		
	eta	$[10^{-5}, 10^{5}]$		
	grow_policy	{depthwise, lossguide}		
	aft_loss_distribution	{normal, logistic, extreme}		
	aft_loss_distribution_scale	$[0.5, 2]$		
	type	hybrid		
	diff.meth	makediff3		
SSVM[5]	gamma.mu	$([2^{-10}, 2^{10}], [2^{-10}, 2^{10}])$	✓	✓
	kernel	{lin_kernel, rbf_kernel, add_kernel}		
	kernel.pars	$[2^{-5}, 2^{5}]$		

[1] Hyper-parameters for model tuning. The choice of hyper-parameters is largely informed by recommendations from the model author and subsequent papers exploring optimization. A '-' indicates no tuning is performed.
[2] Value ranges for the respective hyper-parameters to tune over. Omitted parameters use the package defaults.
[3] Pre-processing of covariates by scaling to unit variance and centering to zero mean is performed (✓) before training the model, or (✗) if not.
[4] Pre-processing of covariates by treatment encoding with `model.matrix` is performed (✓) before training the model, or (✗) if not.

D Implementation, Reproducibility, Accessibility

Platform All experiments were conducted on R 4.4.3 (2025-02-28) – "Trophy Case" on the Beartooth Computing Environment [Beartooth Computing Environment, x86_64 cluster, 2024].

Reproducibility and Accessibility Seeds were set with L'Ecuyer's random number generator [L'Ecuyer, 1999] to ensure reproducible results. All code required to run the experiments, as well as the results, are freely available in a public GitHub repository (`https://github.com/slds-lmu/paper_2023_survival_benchmark`). Software, packages, and version numbers that were utilized to conduct benchmark and analysis are listed below. We also employ the *renv* R package [Ushey and Wickham, 2024] to record and restore package dependencies to further aid reproducibility.

Packages All experiments and analyses are implemented in R and the experiment was run with *batchtools* [Lang et al., 2017]. Learners are implemented in *mlr3proba* [Sonabend et al., 2021], *mlr3extralearners* [Fischer et al., 2025, Sonabend and Schratz, 2024] and *survivalmodels* [Sonabend, 2020]. Tuning is implemented in *mlr3tuning* [Lang et al., 2023]. Benchmarking functionality is implemented in *mlr3* [Lang et al., 2019]. Statistical benchmark analysis is implemented in *mlr3benchmark* [Sonabend and Pfisterer, 2020]. Prediction type compositions and pre-processing steps are implemented through *mlr3pipelines* [Binder et al., 2021]. The implementing packages for all algorithms are given in Table 3 and all measures are implemented in *mlr3proba*. The Plackett-Luce model used for sensitivity analysis is implemented in *PlackettLuce* [Turner et al., 2020, 2017] and the Plackett-Luce trees implemented there rely on *partykit* [Hothorn and Zeileis, 2015].

E Results

The following figures show boxplots and violin plots of the respective evaluation measure across all outer resampling iterations. Results are also available following links on the GitHub repository at `https://github.com/slds-lmu/paper_2023_survival_benchmark`.

As described in **??**, we provide aggregated results based on average ranks across all datasets as boxplots and violin plots with two versions for discrimination measures and three different scaling options for scoring rules:

1. Raw scores as produced by the evaluation measures, e.g. "ISBS",
2. Explained Residual Variation (ERV) scores, e.g. "ISBS [ERV]",
3. Scaled scores, e.g. "ISBS [Scaled]", which scales raw scores such that 0 is the score achieved by KM and 1 is the score achieved by the best-performing model within the given combination of dataset and tuning- and evaluation measure.

Since the discrimination measures Harrell's C and Uno's C are already scaled from 0 (worst) to 1 (best) with KM achieving a score of 0.5 by design, the ERV option is omitted and only options 1) and 3) are presented.

E.1 Discrimination Measures

E.1.1 Raw Scores

Boxplots of raw evaluation scores using discrimination measures for tuning (Harrell's C)

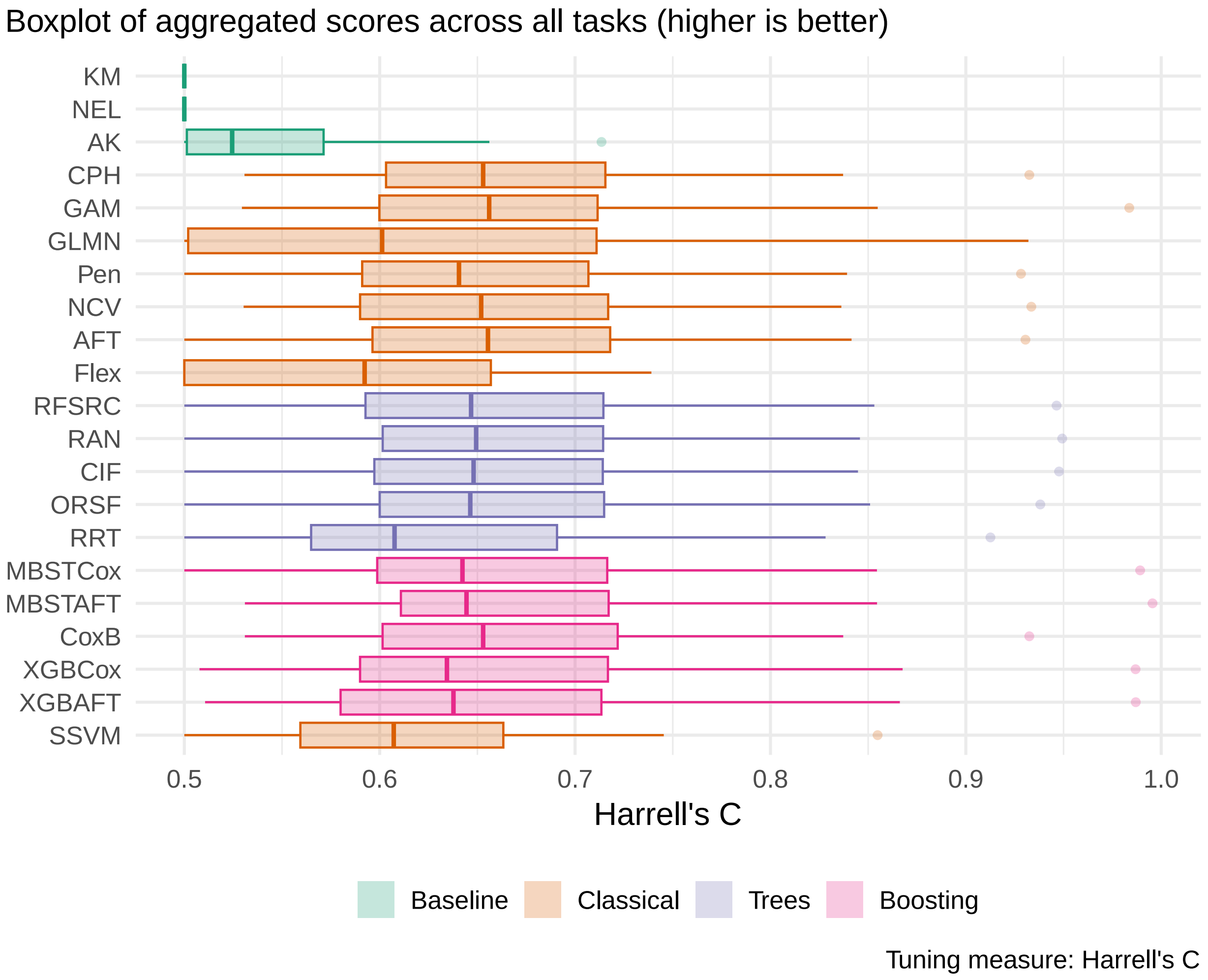

Figure 1: Learners tuned and evaluated with Harrell's C

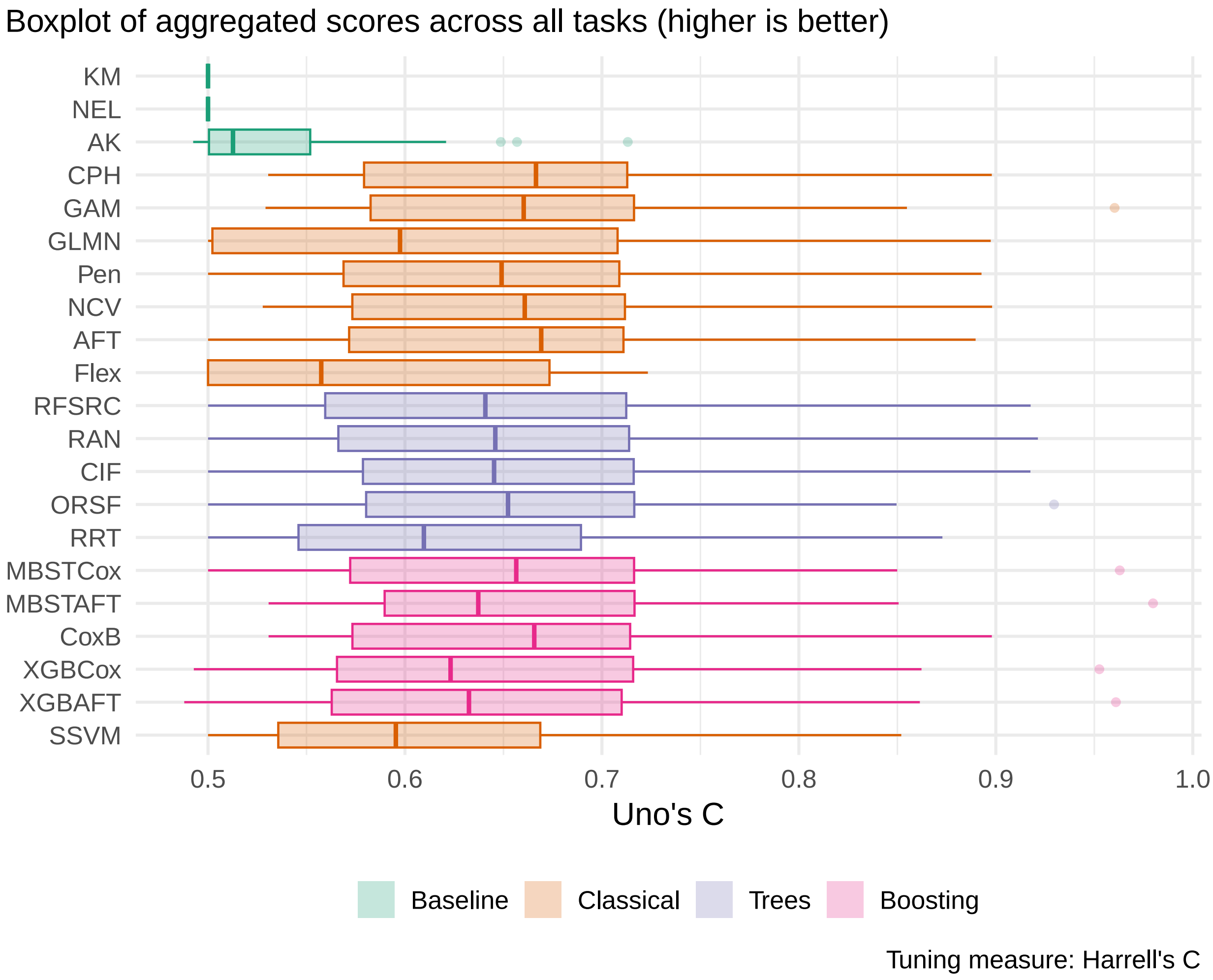

Figure 2: Learners tuned for Harrell's C and evaluated with Uno's C

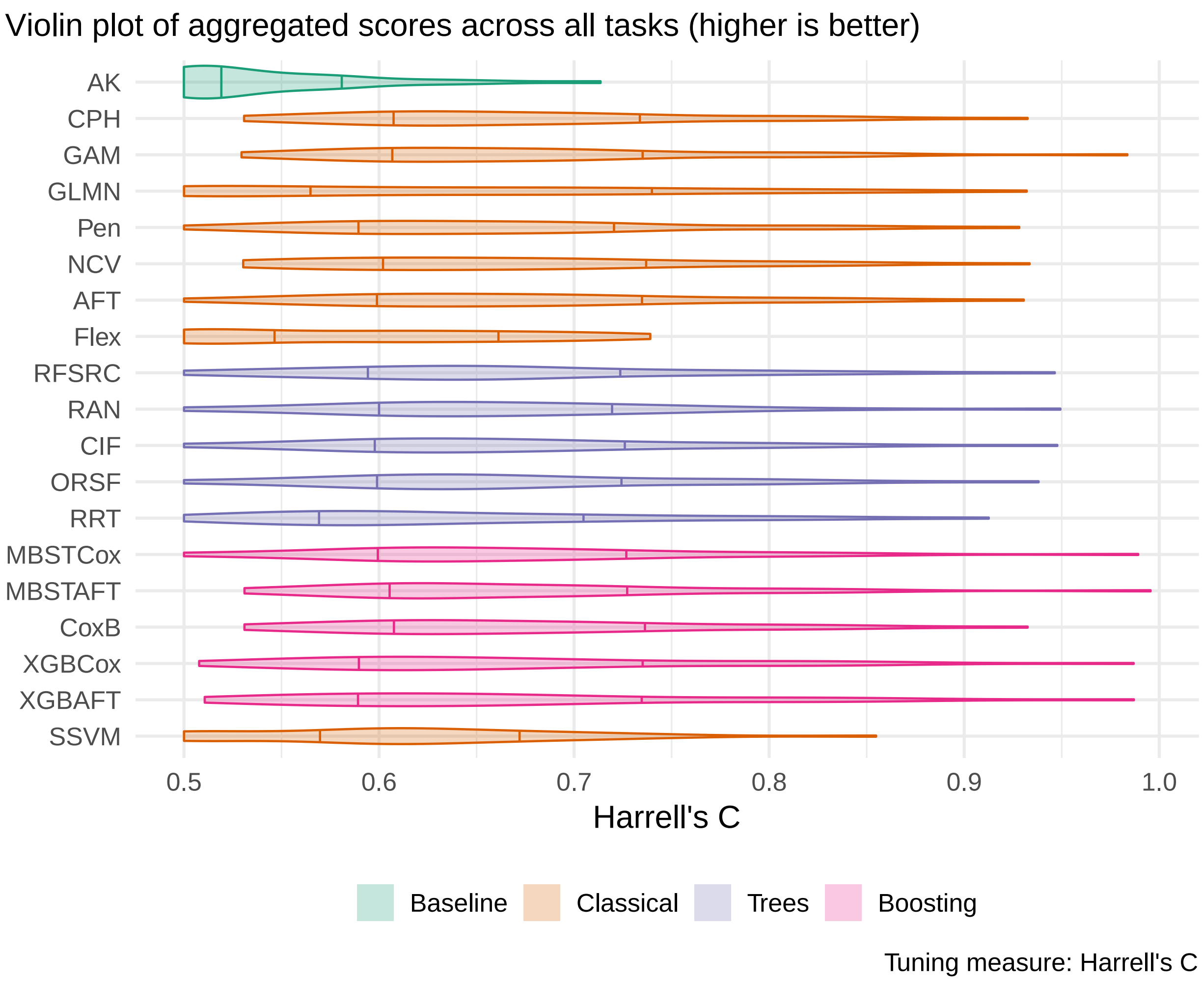

Figure 3: Learners tuned and evaluated with Harrell's C (violin plot)

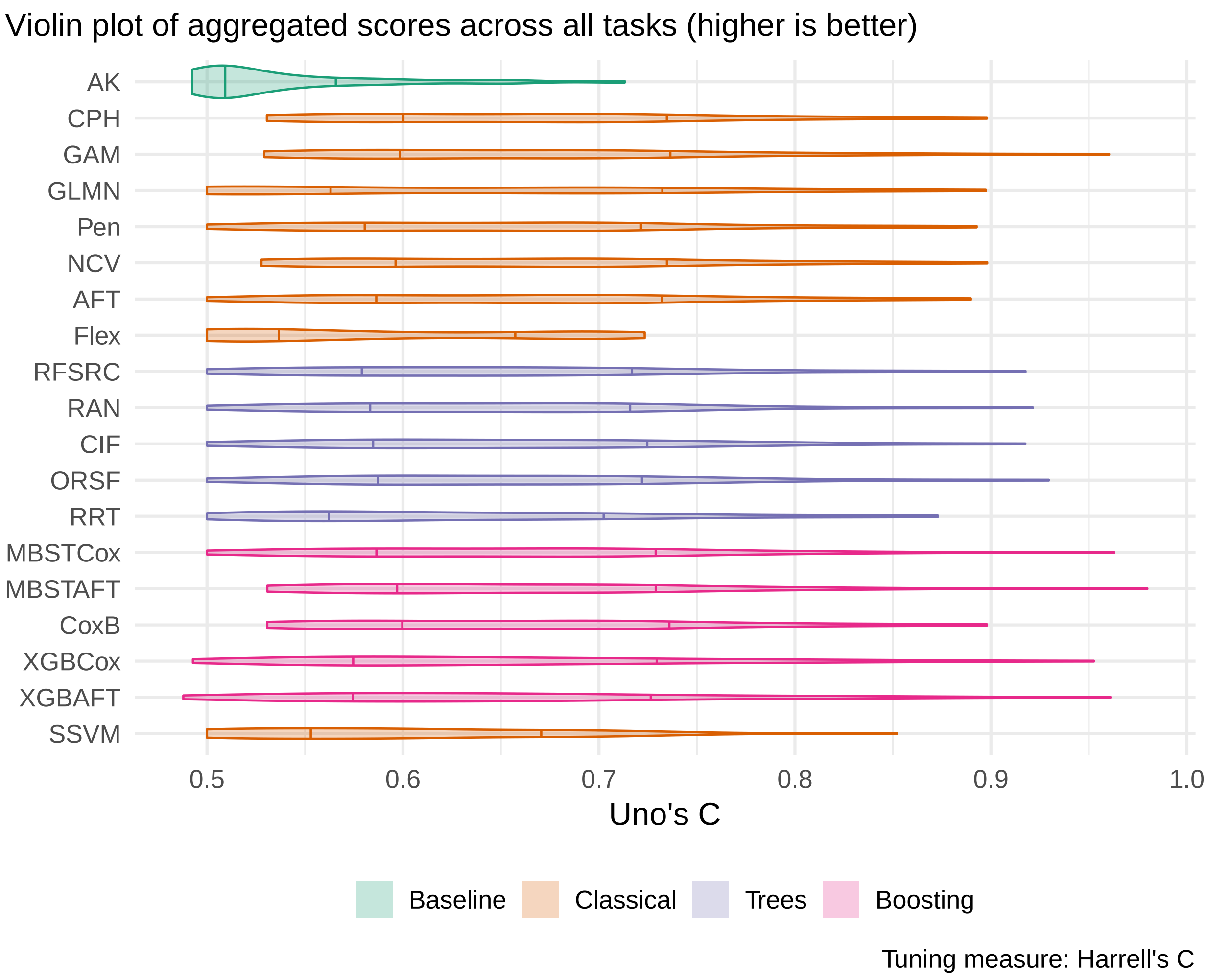

Figure 4: Learners tuned for Harrell's C and evaluated with Uno's C (violin plot)

E.1.2 Scaled from worst (0) to best (1)

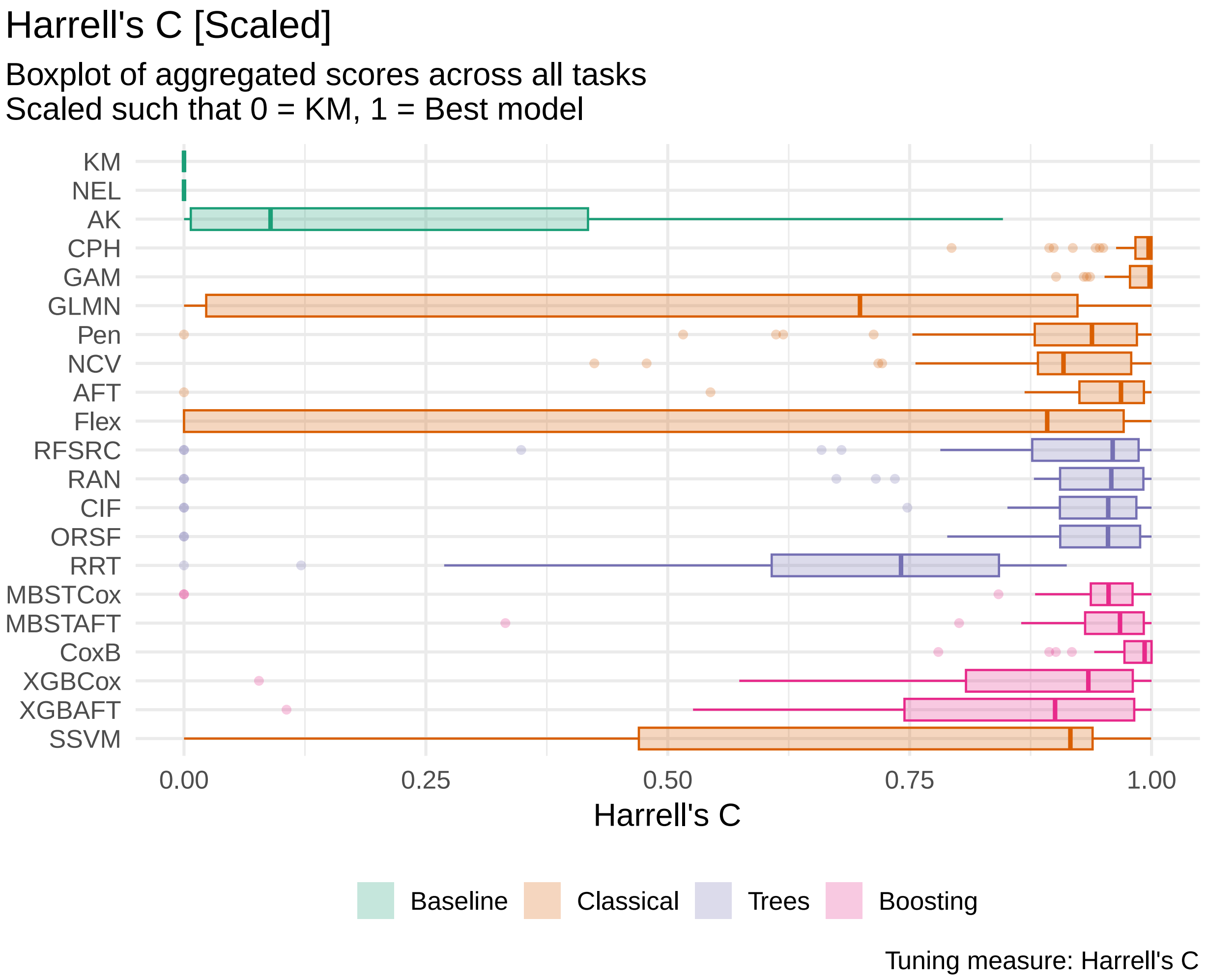

Figure 5: Learners tuned and evaluated with Harrell's C (scaled scores)

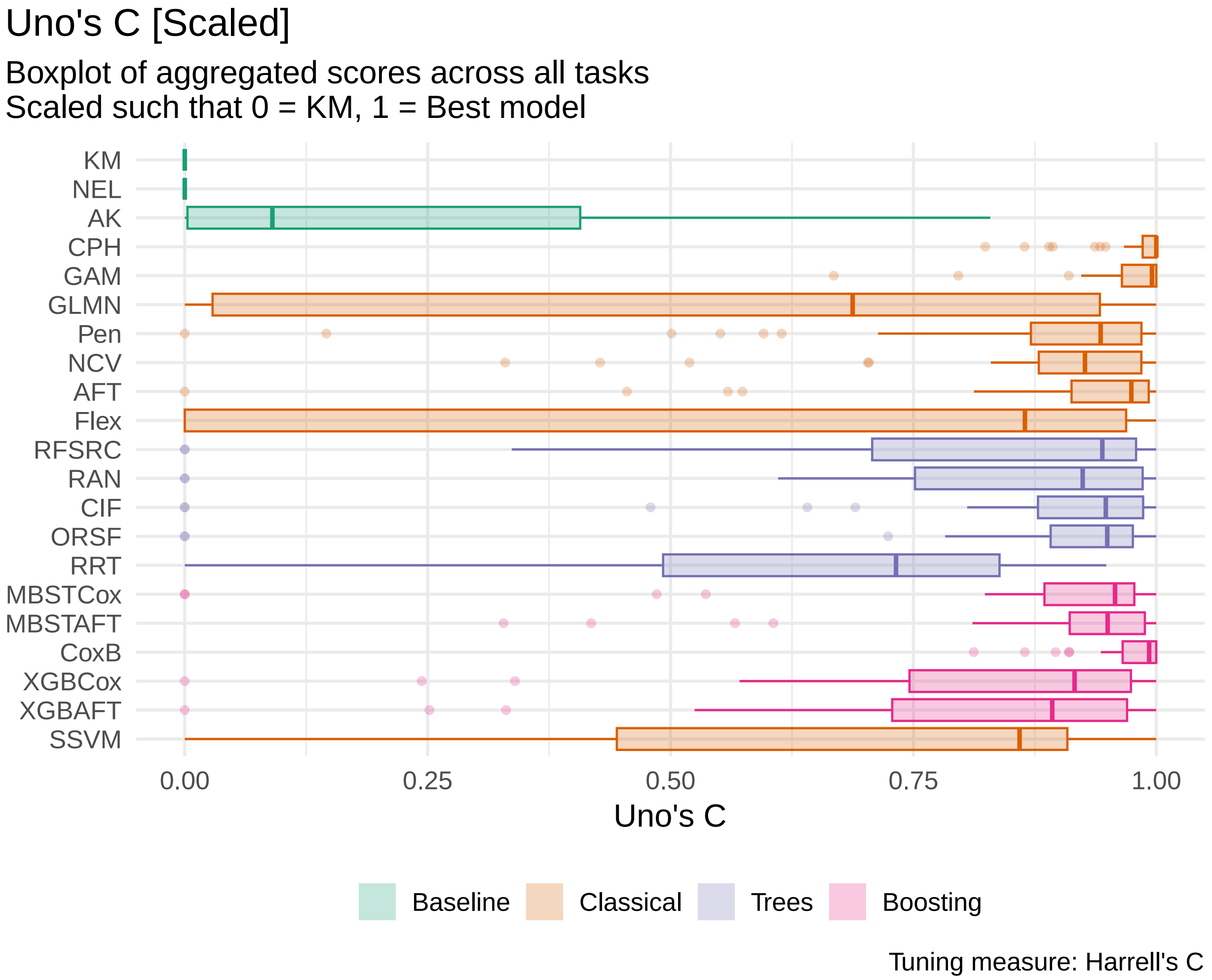

Figure 6: Learners tuned for Harrell's C and evaluated with Uno's C (scaled scores)

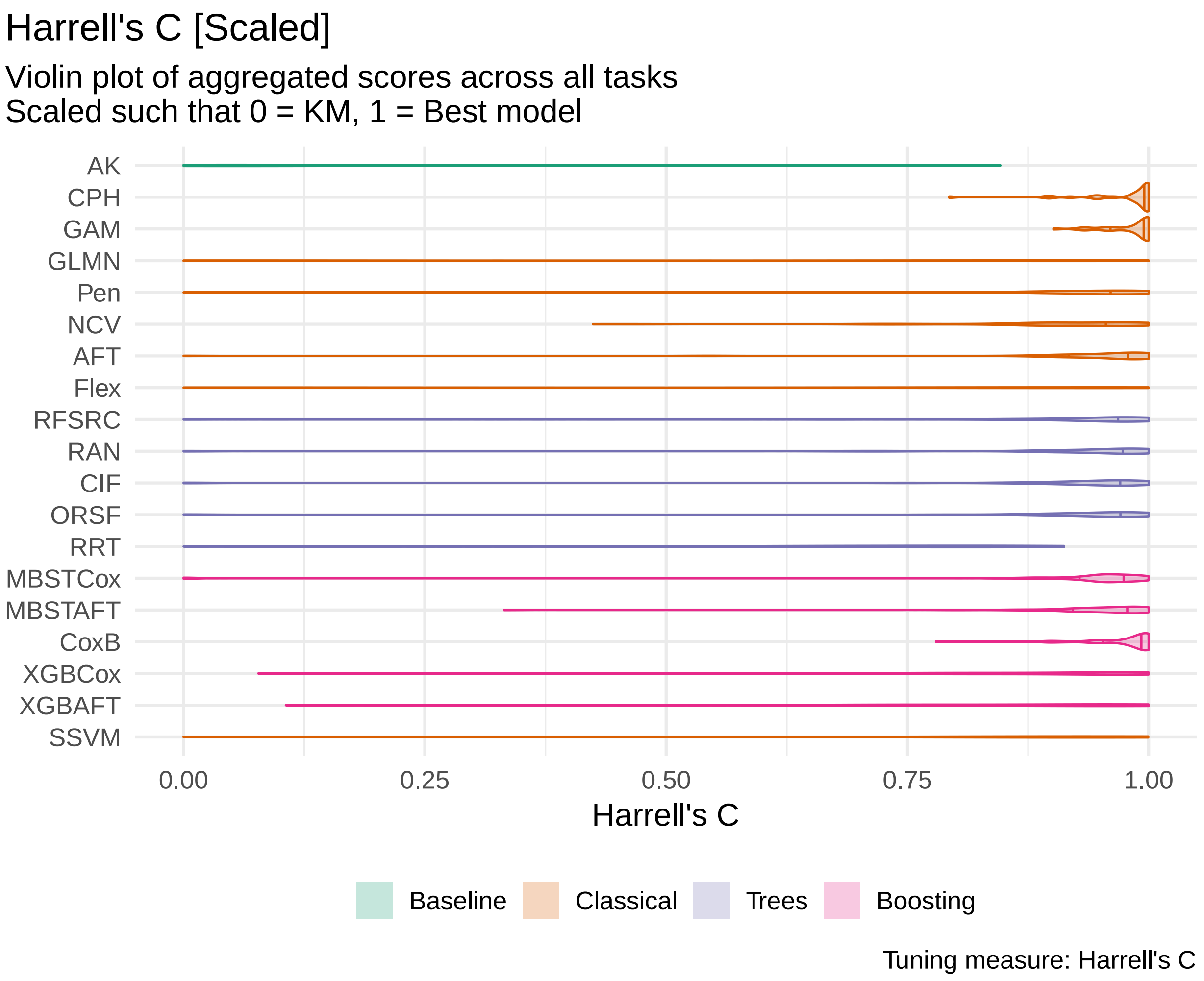

Figure 7: Learners tuned and evaluated with Harrell's C (scaled scores, violin plot)

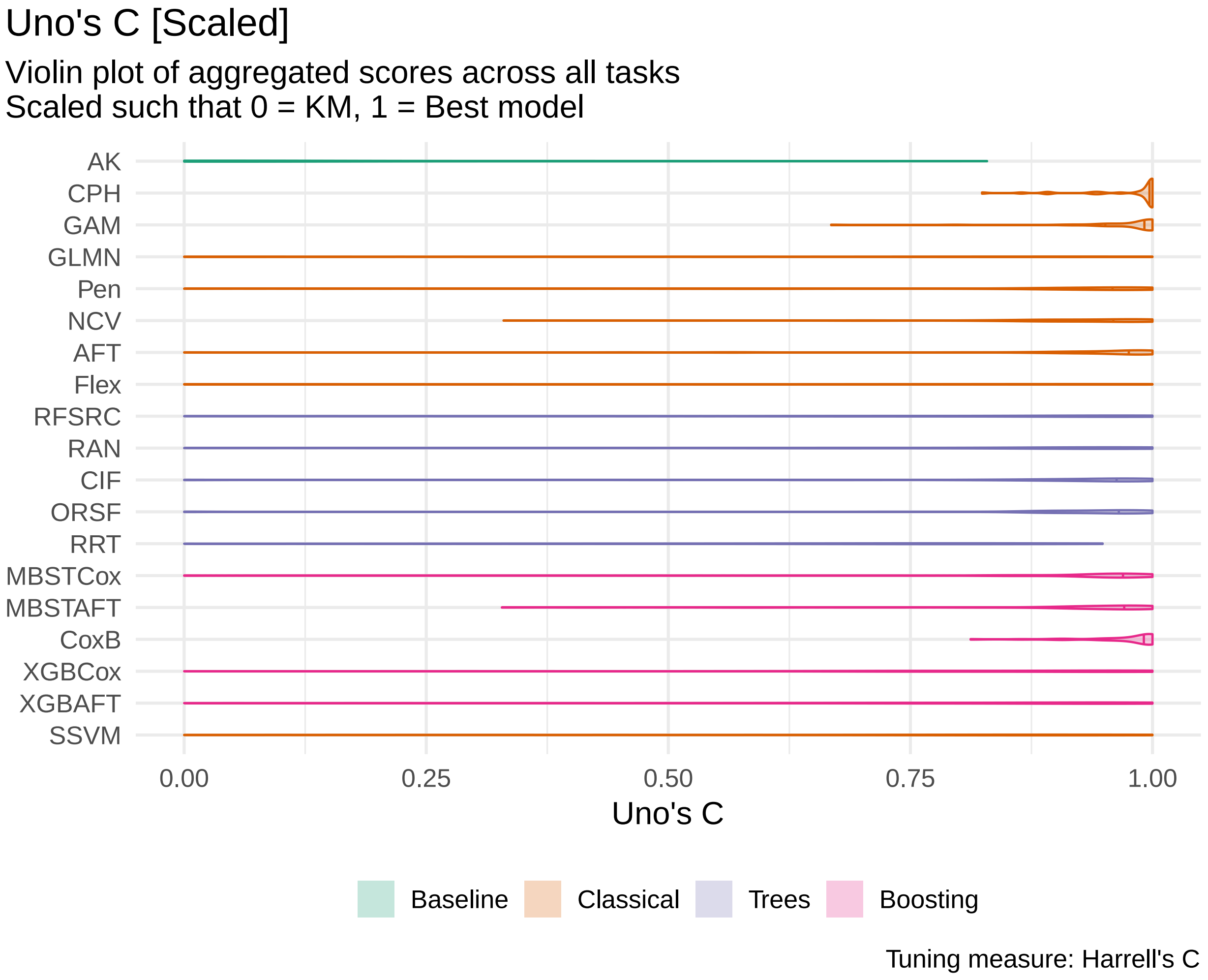

Figure 8: Learners tuned for Harrell's C and evaluated with Uno's C (scaled scores, violin plot)

E.2 Scoring Rules

E.2.1 Raw Scores

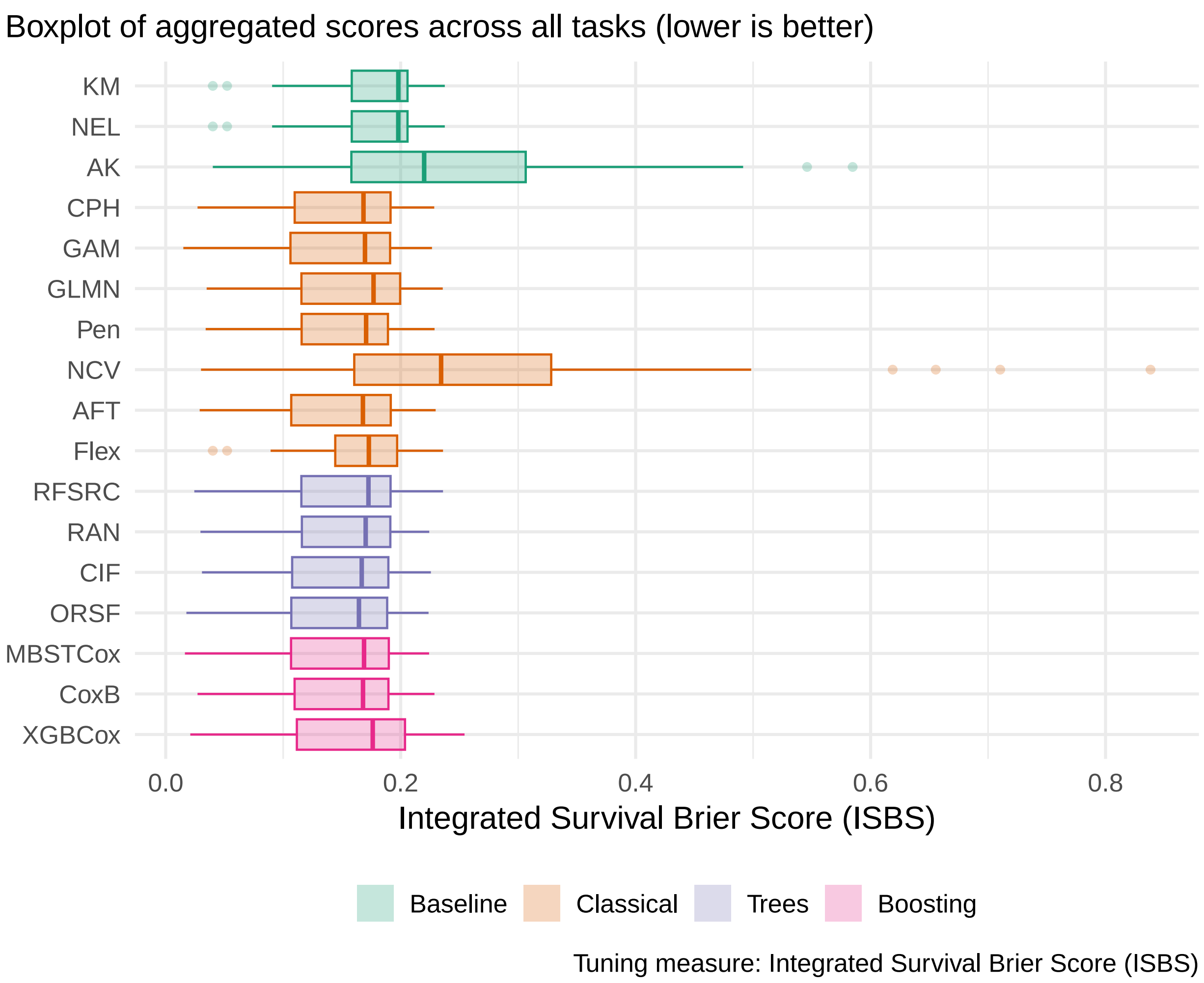

Figure 9: Learners tuned for ISBS and evaluated with ISBS (raw scores)

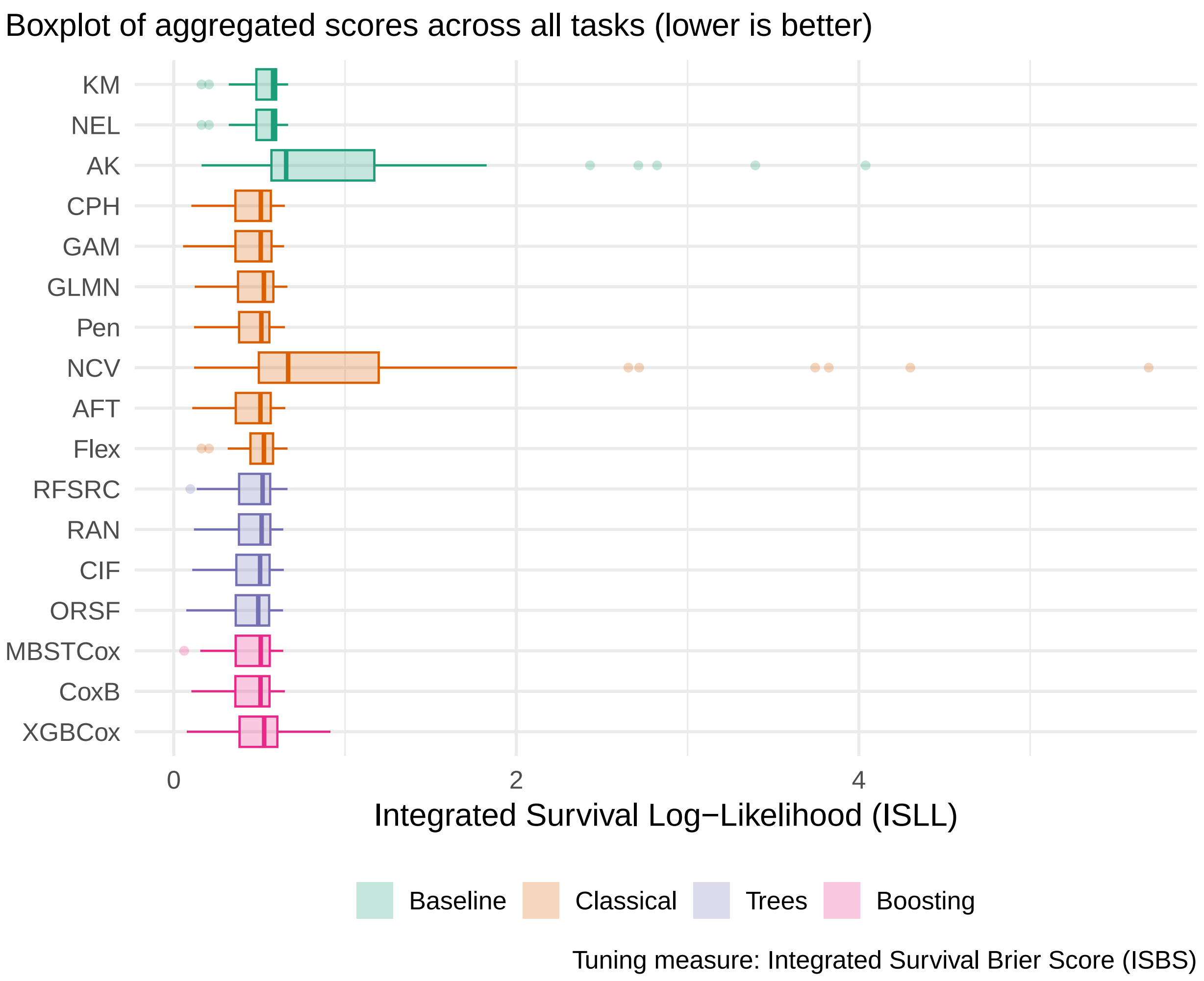

Figure 10: Learners tuned for ISBS and evaluated with ISLL (raw scores)

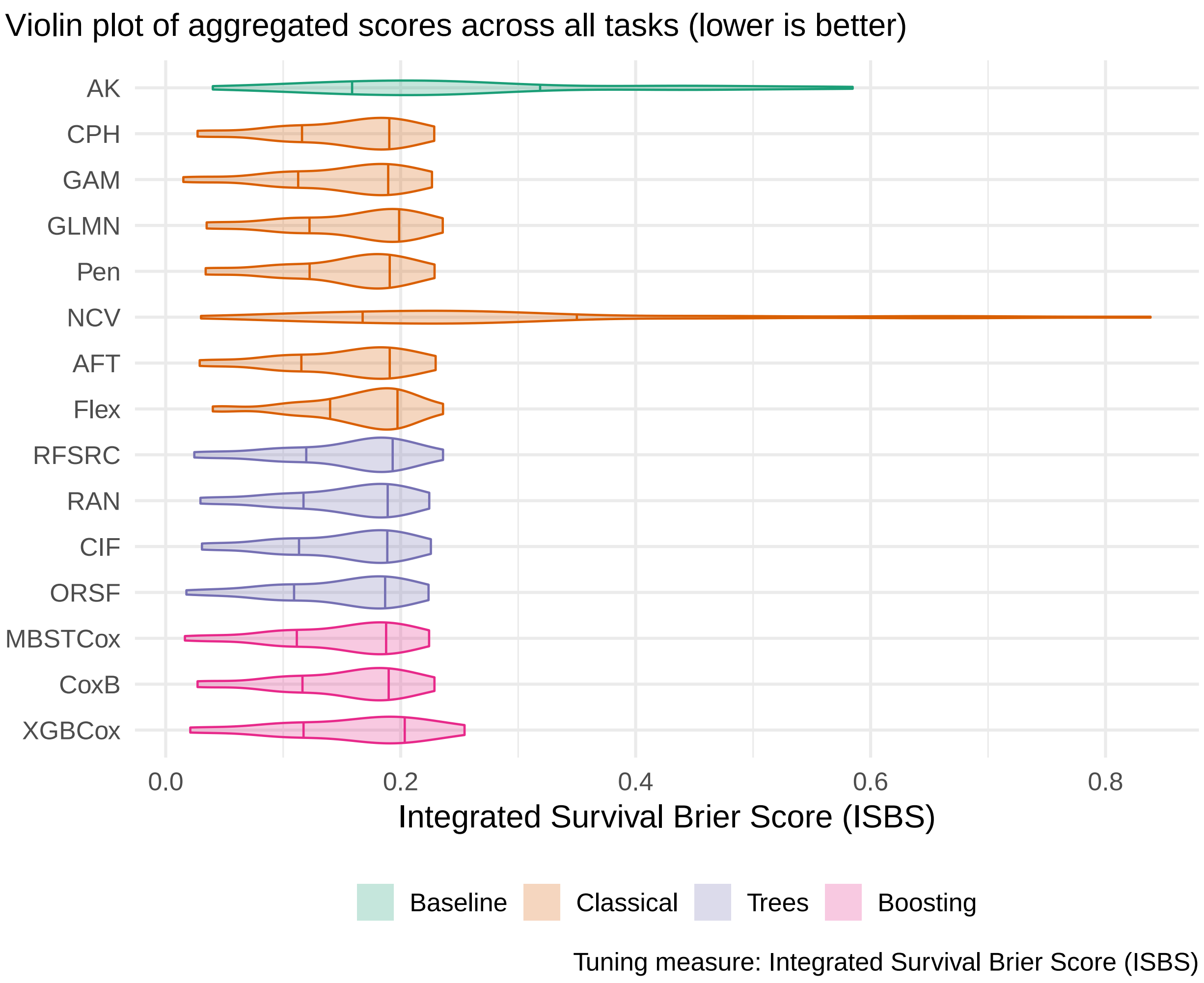

Figure 11: Learners tuned for ISBS and evaluated with ISBS (raw scores, violin plot)

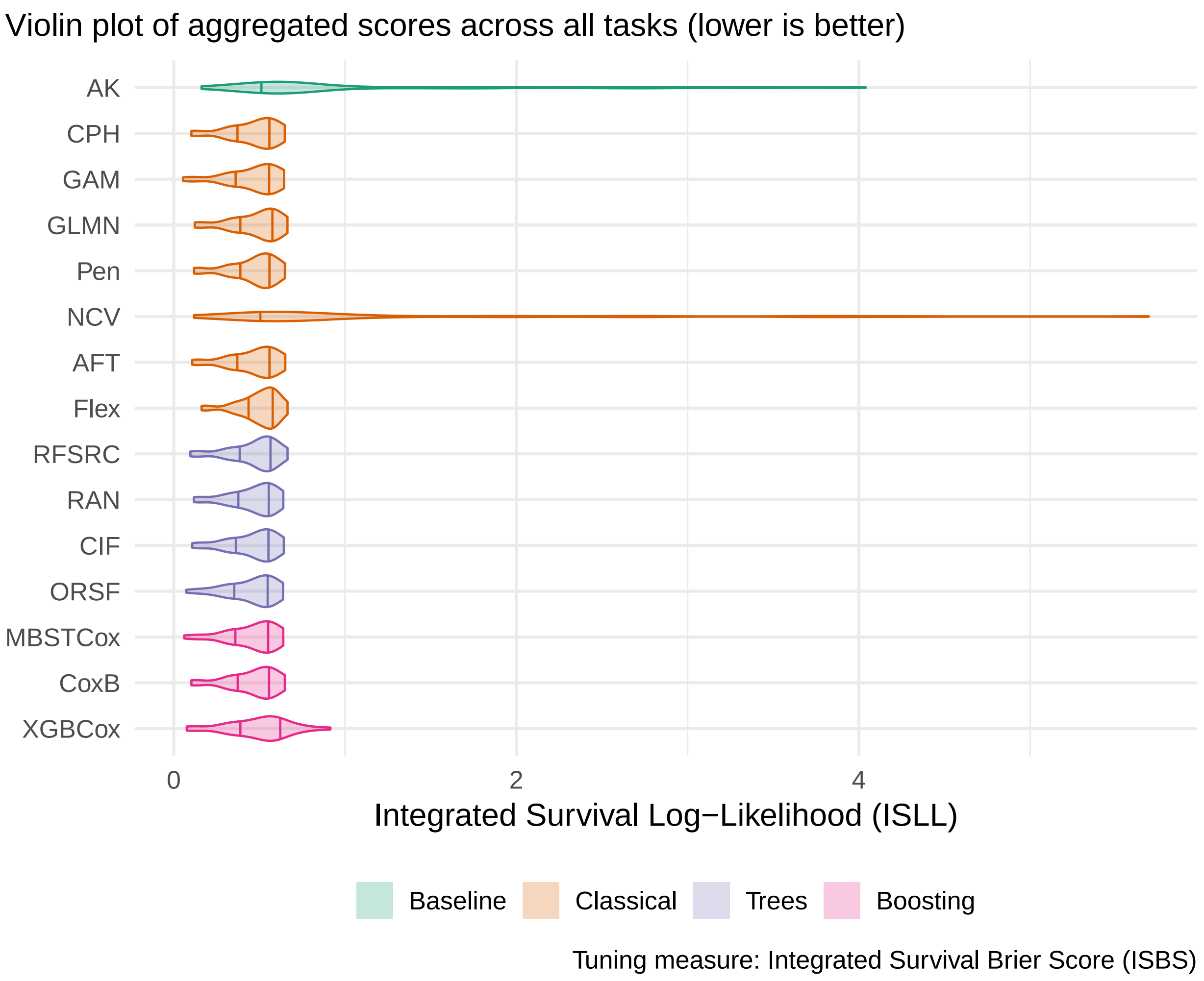

Figure 12: Learners tuned for ISBS and evaluated with ISLL (raw scores, violin plot)

E.2.2 ERV

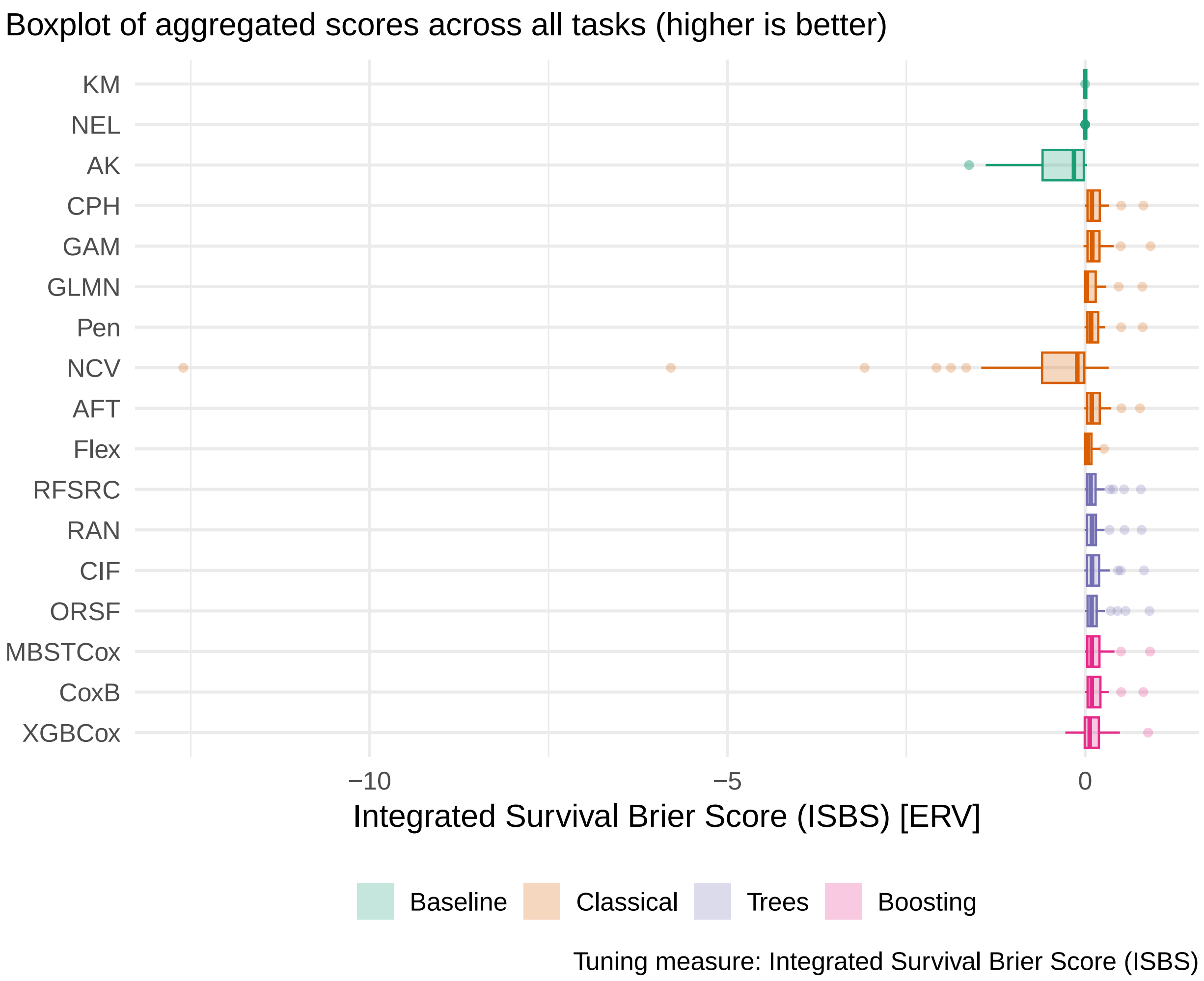

Figure 13: Learners tuned for ISBS and evaluated with ISBS (ERV)

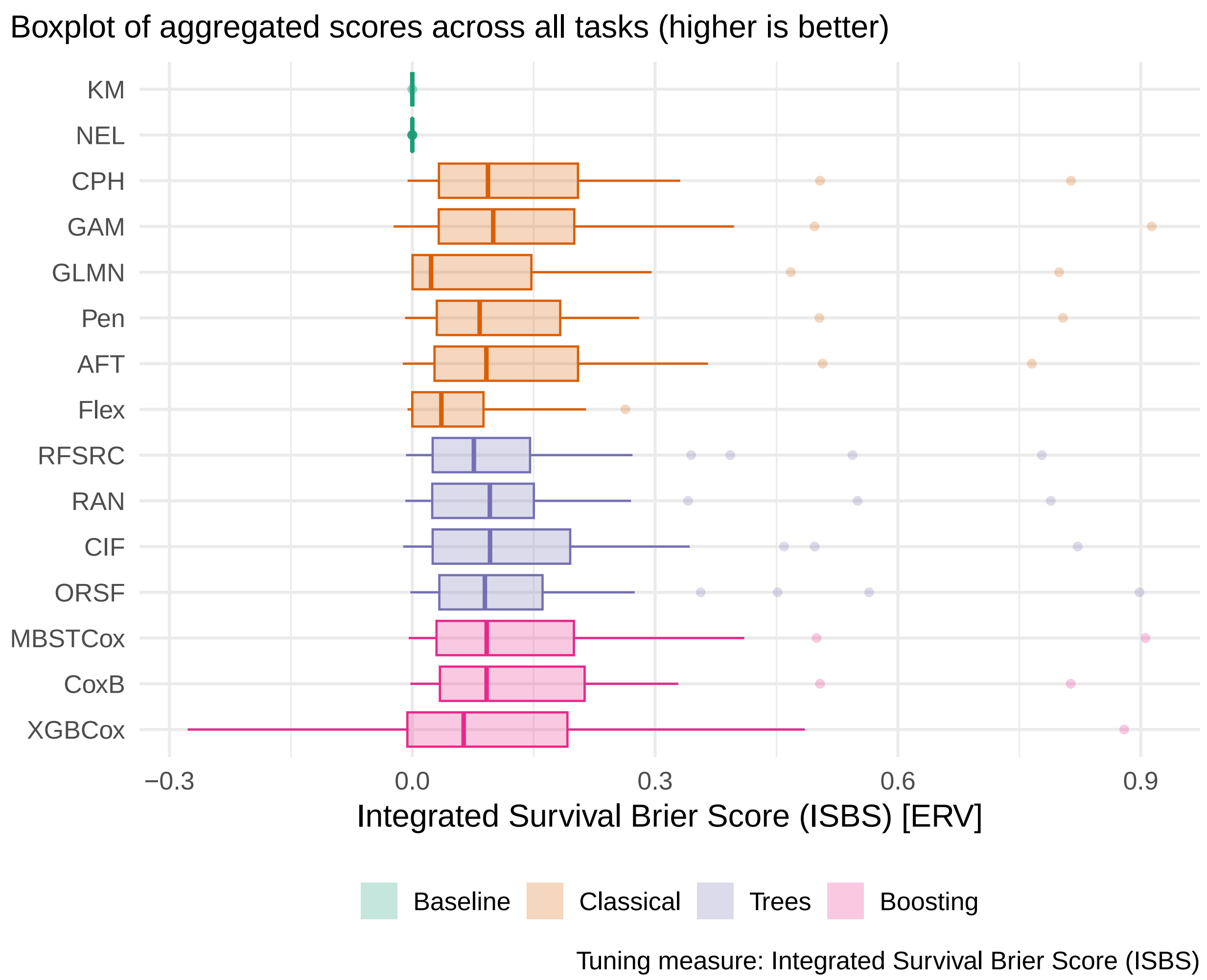

Figure 14: Learners tuned for ISBS and evaluated with ISBS (ERV). Learners with excessive outliers are excluded for readability.

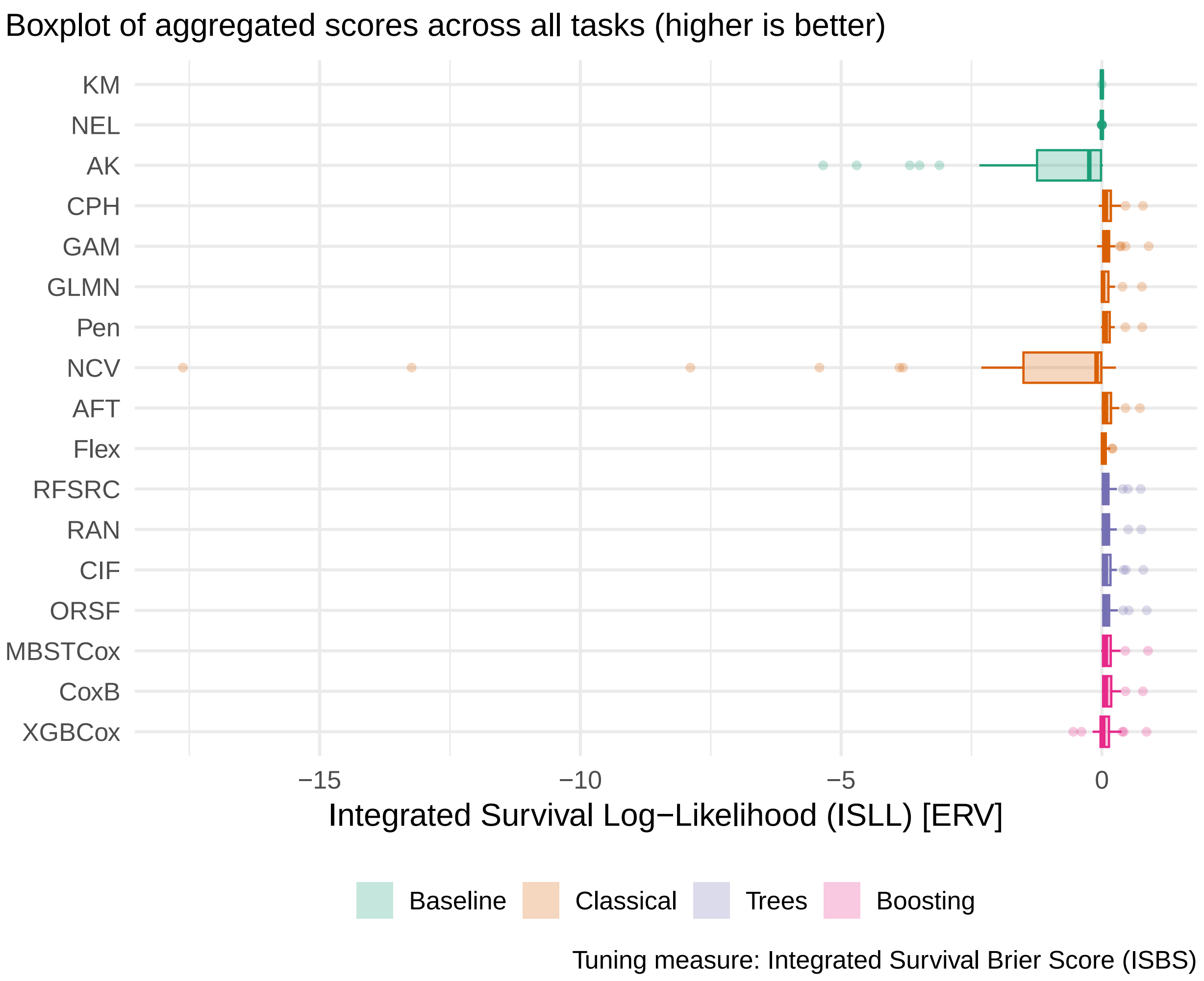

Figure 15: Learners tuned for ISBS and evaluated with ISLL (ERV)

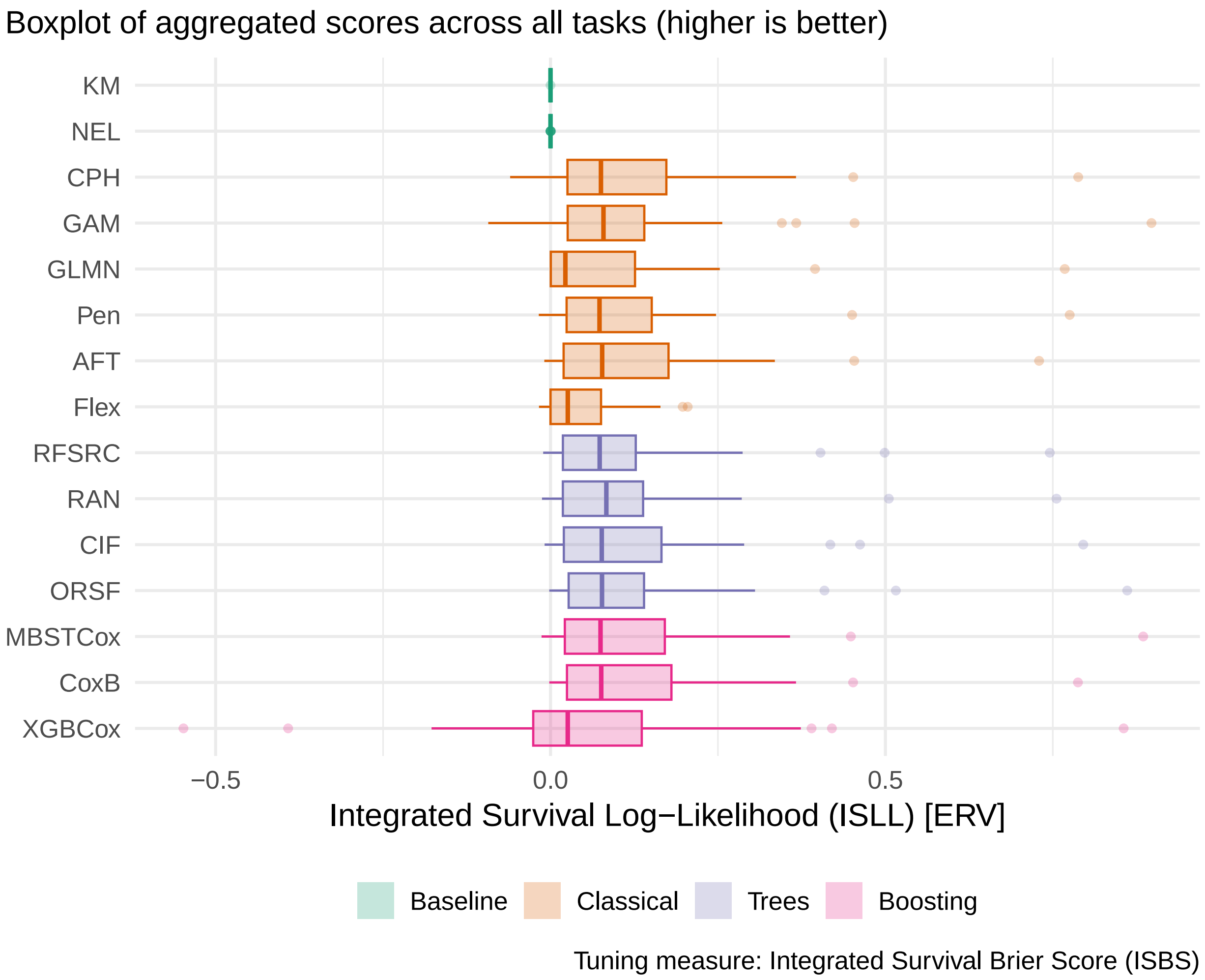

Figure 16: Learners tuned for ISBS and evaluated with ISLL (ERV). Learners with excessive outliers are excluded for readability.

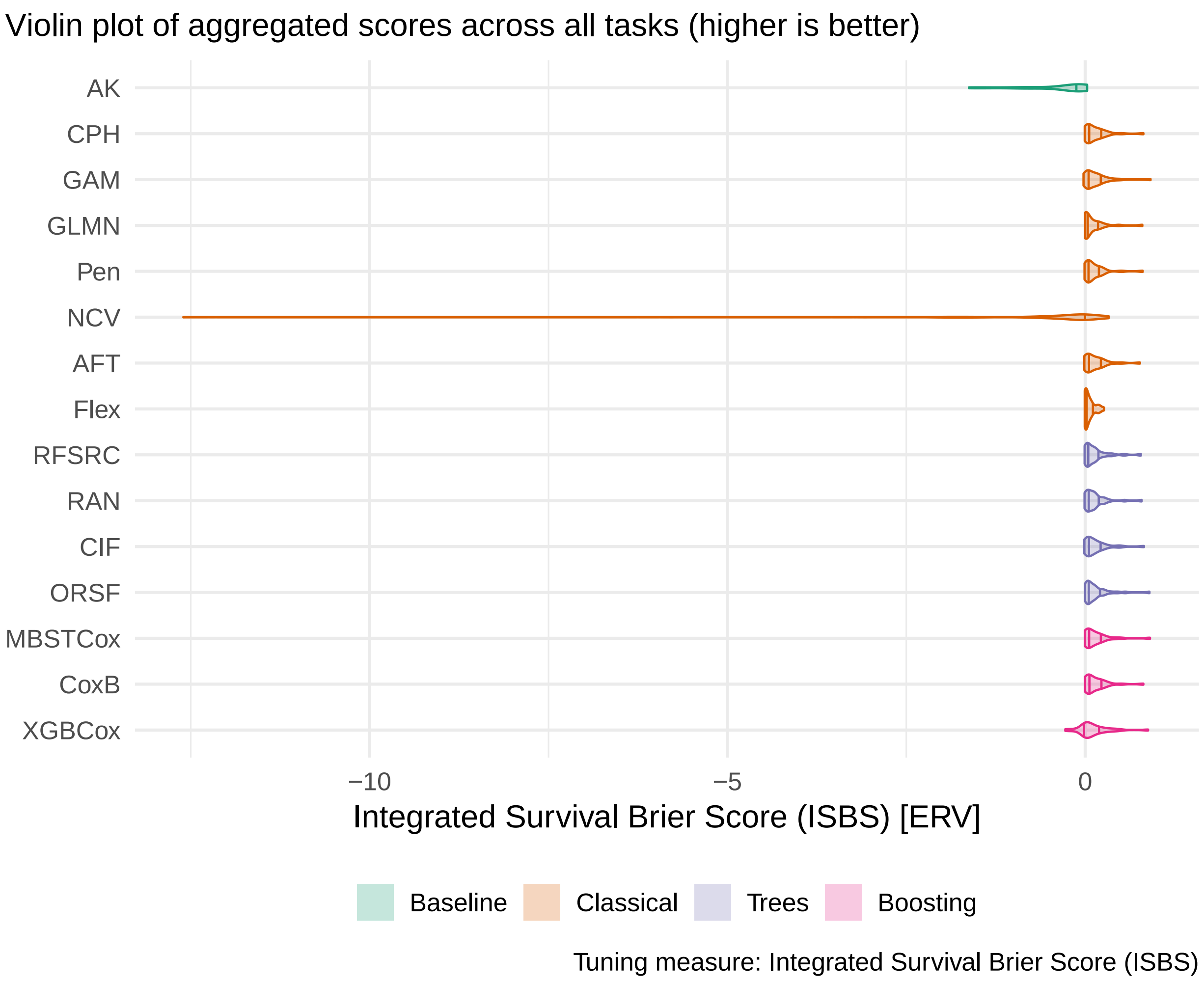

Figure 17: Learners tuned for ISBS and evaluated with ISBS (ERV, violin plot)

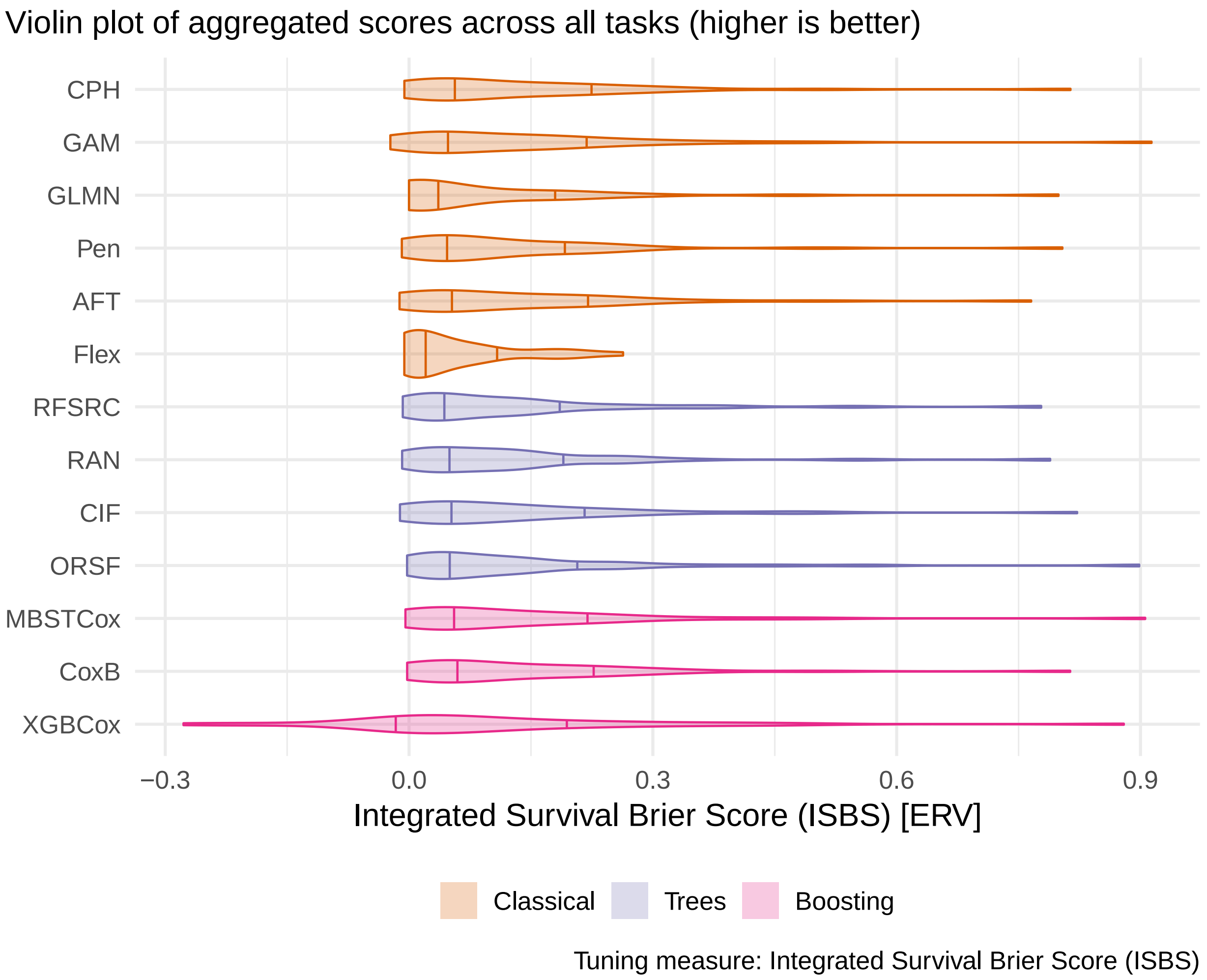

Figure 18: Learners tuned for ISBS and evaluated with ISBS (ERV, violin plot). Learners with excessive outliers are excluded for readability.

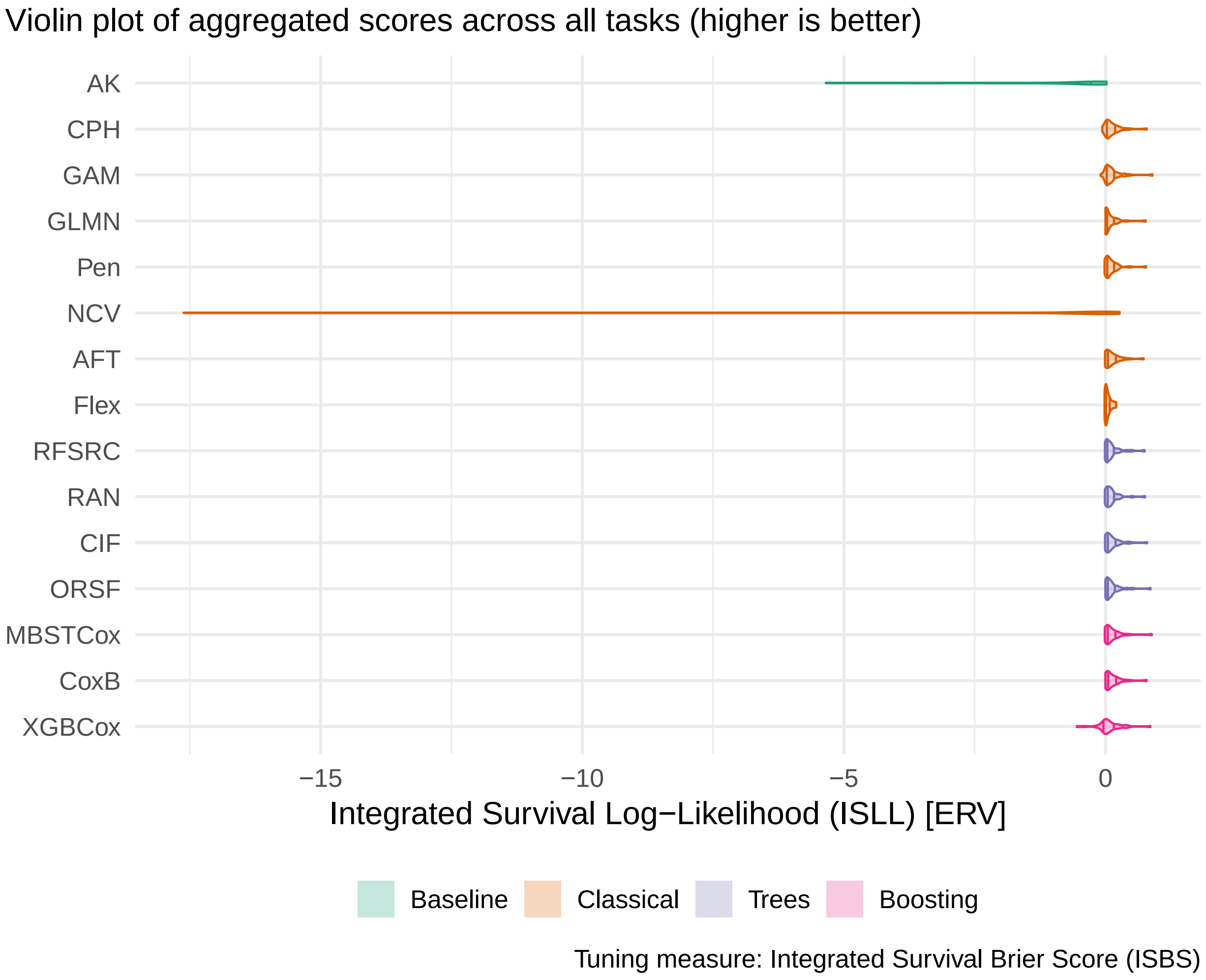

Figure 19: Learners tuned for ISBS and evaluated with ISLL (ERV, violin plot)

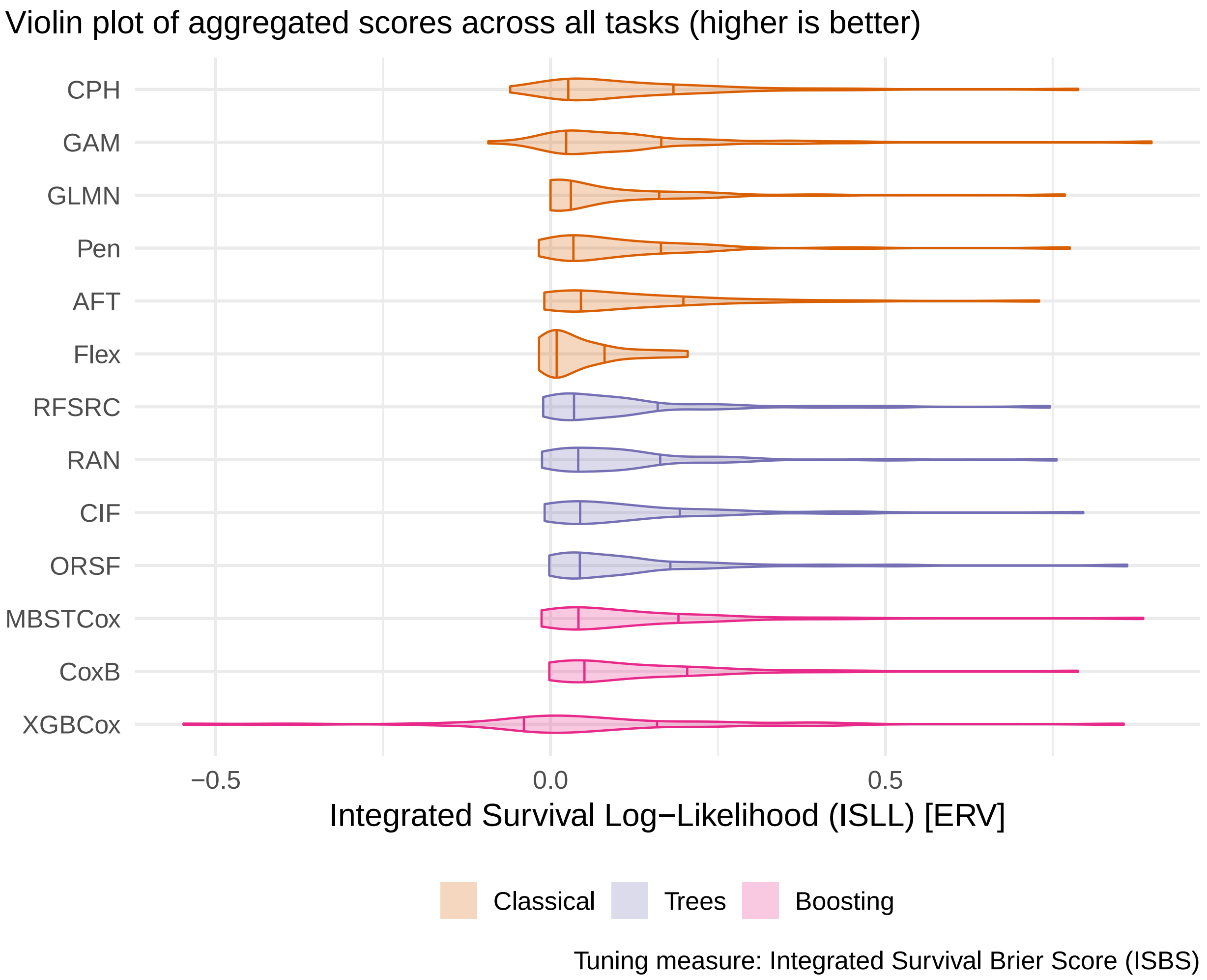

Figure 20: Learners tuned for ISBS and evaluated with ISLL (ERV, violin plot). Learners with excessive outliers are excluded for readability.

E.2.3 Scaled

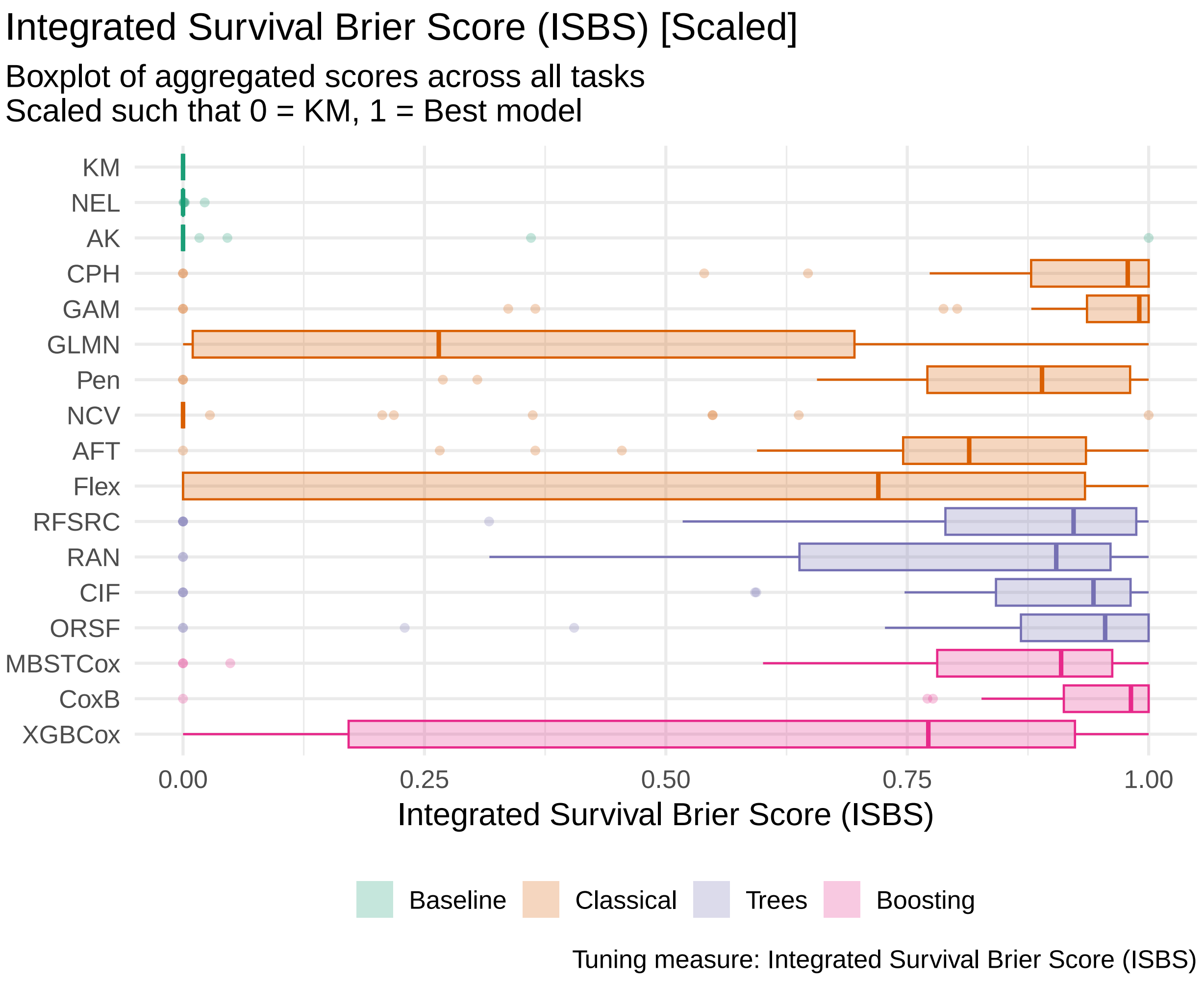

Figure 21: Learners tuned for ISBS and evaluated with ISBS (scaled)

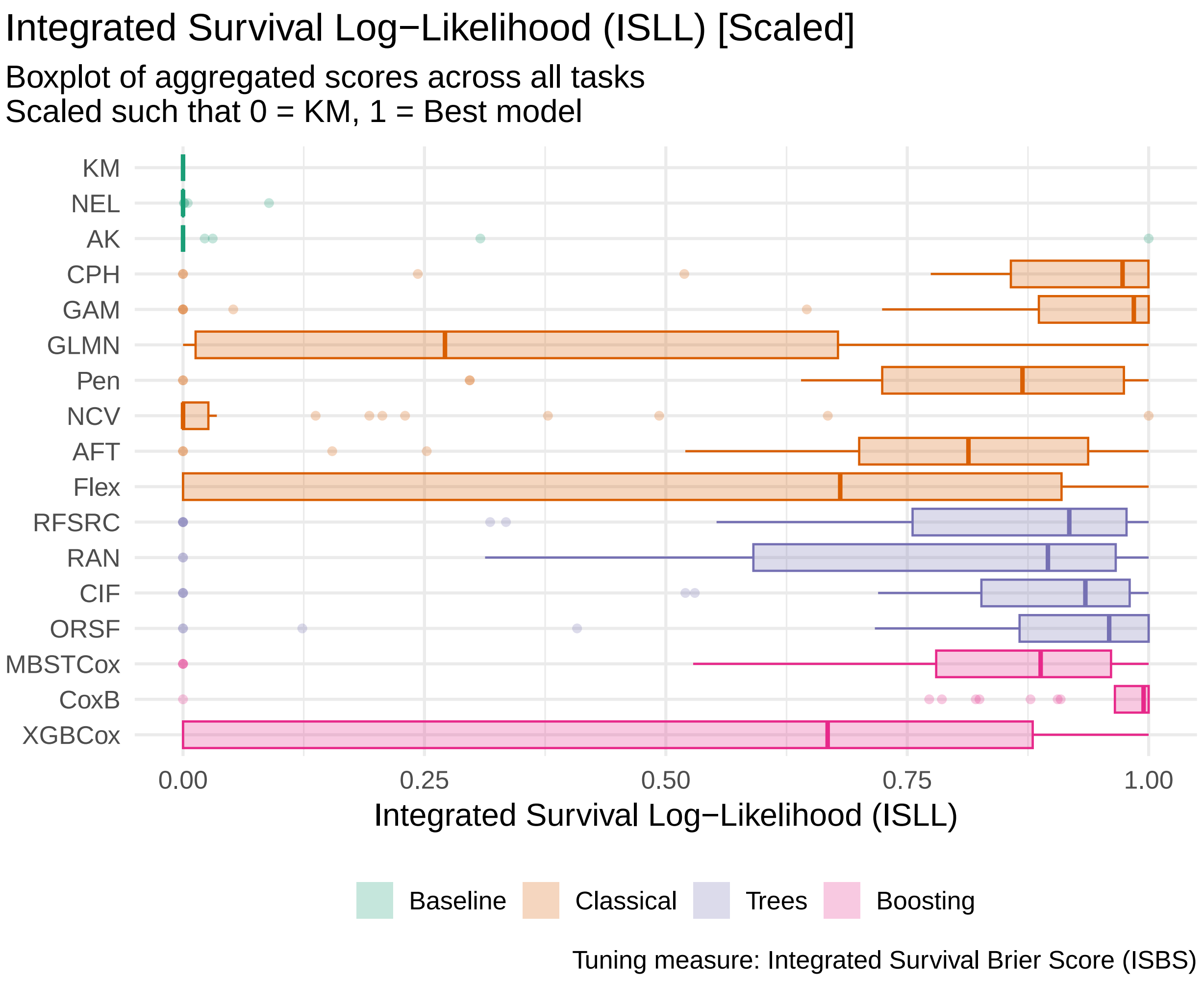

Figure 22: Learners tuned for ISBS and evaluated with ISLL (scaled)

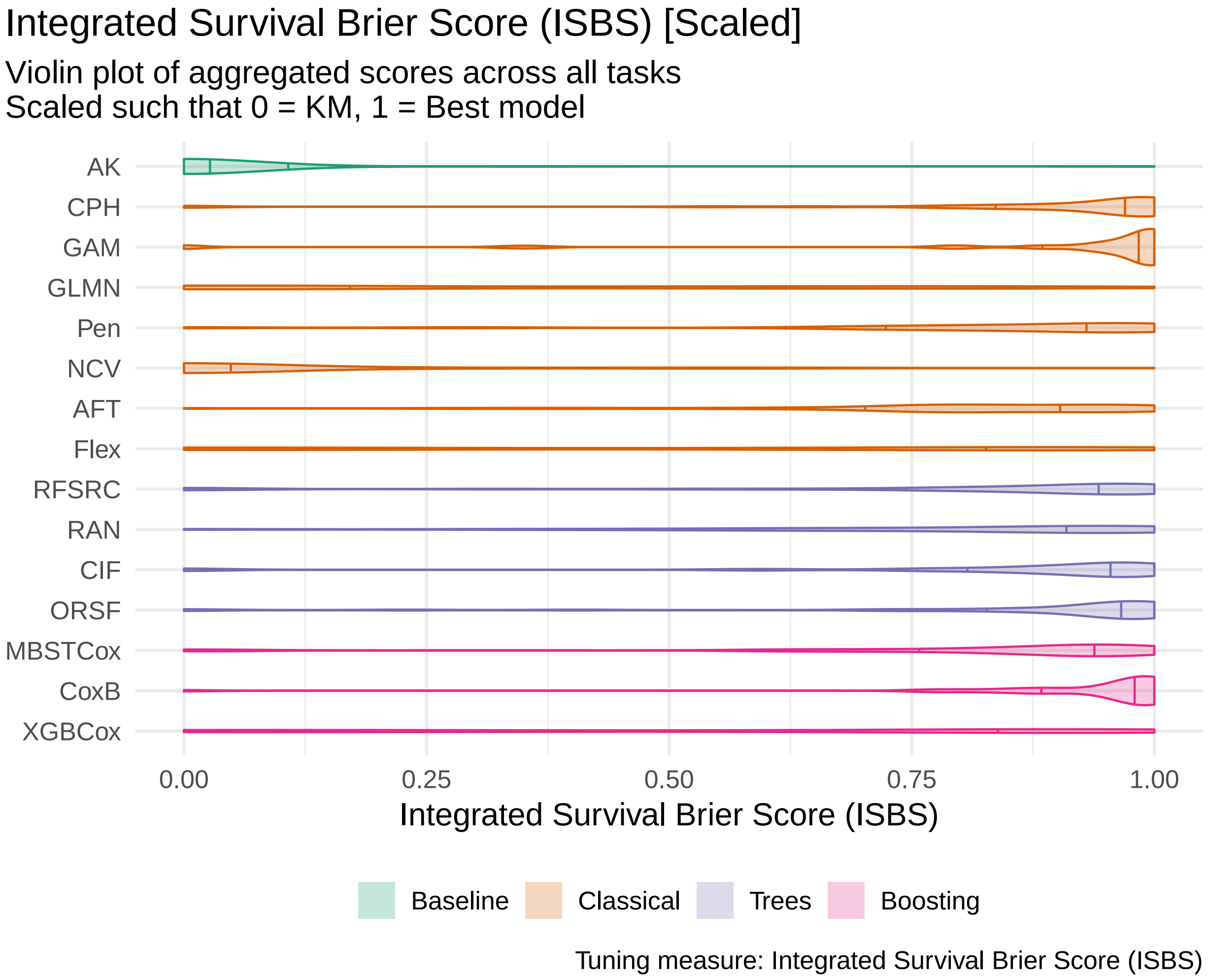

Figure 23: Learners tuned for ISBS and evaluated with ISBS (scaled, violin plot)

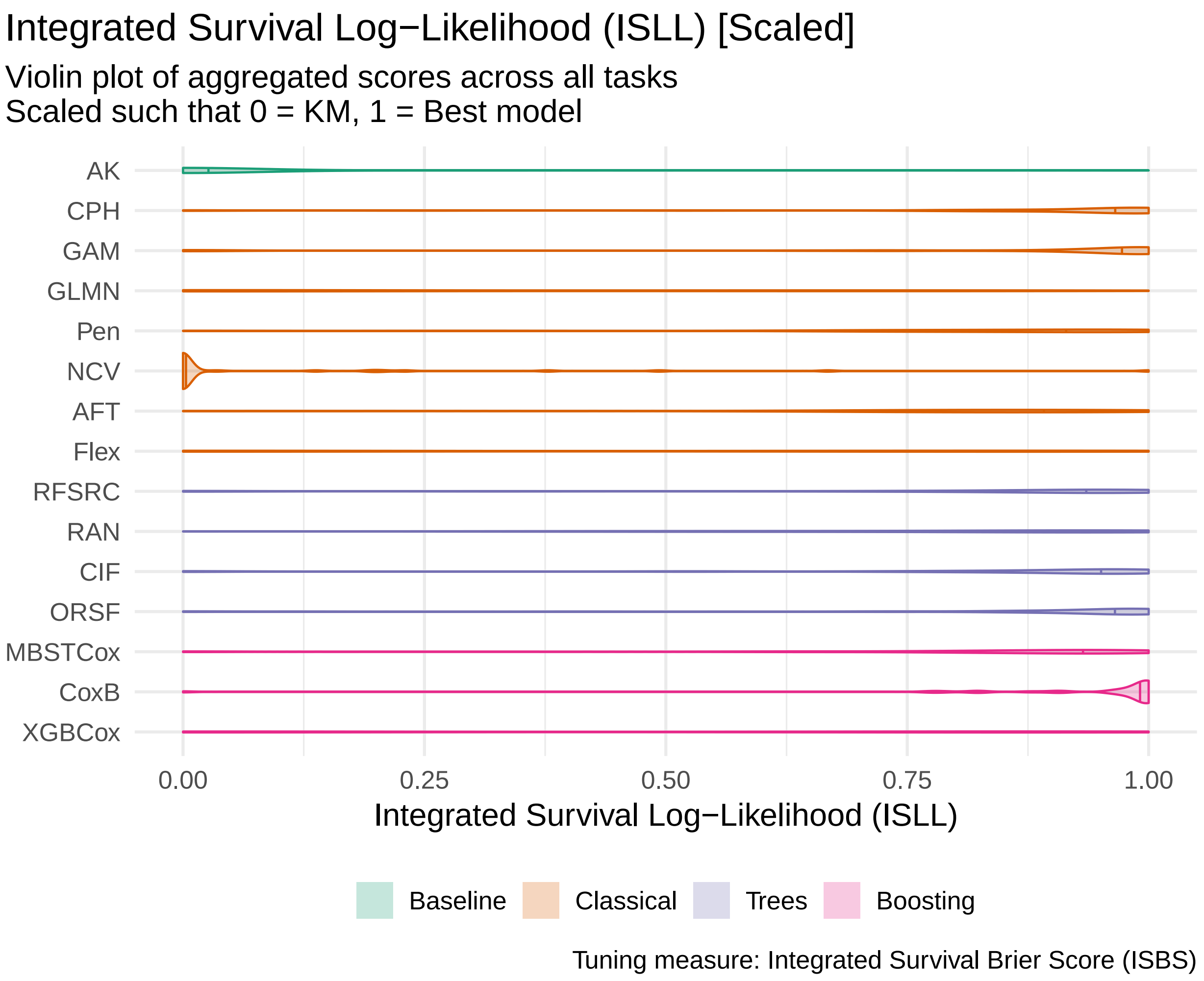

Figure 24: Learners tuned for ISBS and evaluated with ISLL (scaled, violin plot)

E.3 Calibration Measures

Calibration results presented in this section are obtained for models tuned for ISBS and consider calibration as measured by D-Calibration and van Houwelingen's α. Results for these measures are presented to provide additional insights, but we want to note that the measures used are not well-established and are considered experimental with respect to underlying theory and respective implementation.

D-Calibration considers a model well-calibrated if the p-value of the underlying test is greater than 0.05, which Figure 25 shows in the form of a heatmap with X indicating a significant test result, indicating a model is not well-calibrated. Here, NEL, RFSRC, RAN, and ORSF are among the best calibrated, while AK, XGBCox, CIF, AFT and CPH would be considered poorly calibrated.

Van Houwelingen's α is shown in Figure 26 and relates predicted and observed hazards, with a value close to 1 implying a well-calibrated model. Here, KM, NEL, GLMN, and Pen show good calibration, while XGBCox shows a wide spread indicating poor calibration. RFSRC, RAN, and ORSF similarly show more skewed scores, indicating poor calibration.

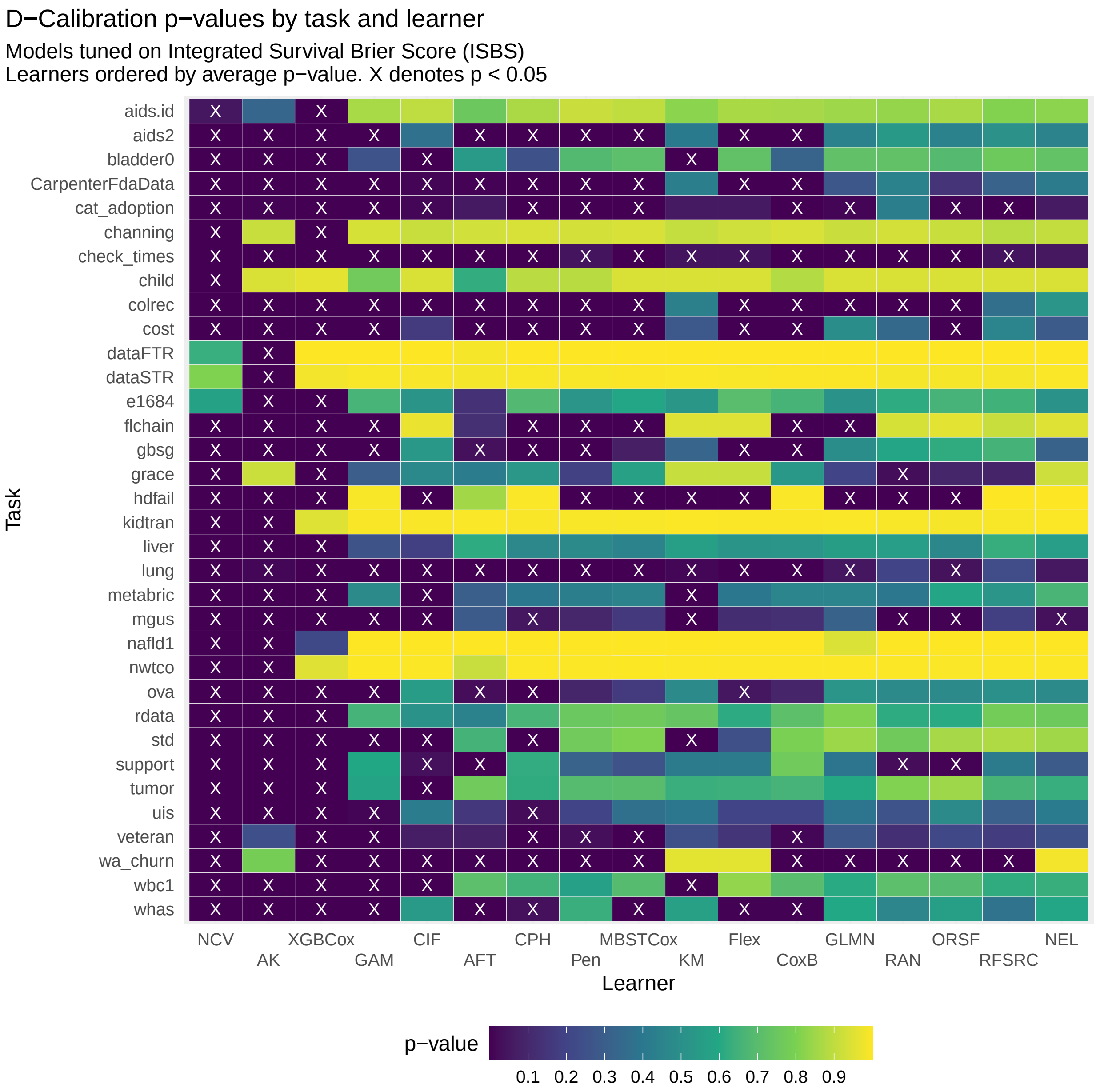

Figure 25: D-Calibration p-value heatmap across all datasets and models. 'X' indicates $p < 0.05$ while a non-significant result indicates good calibration.

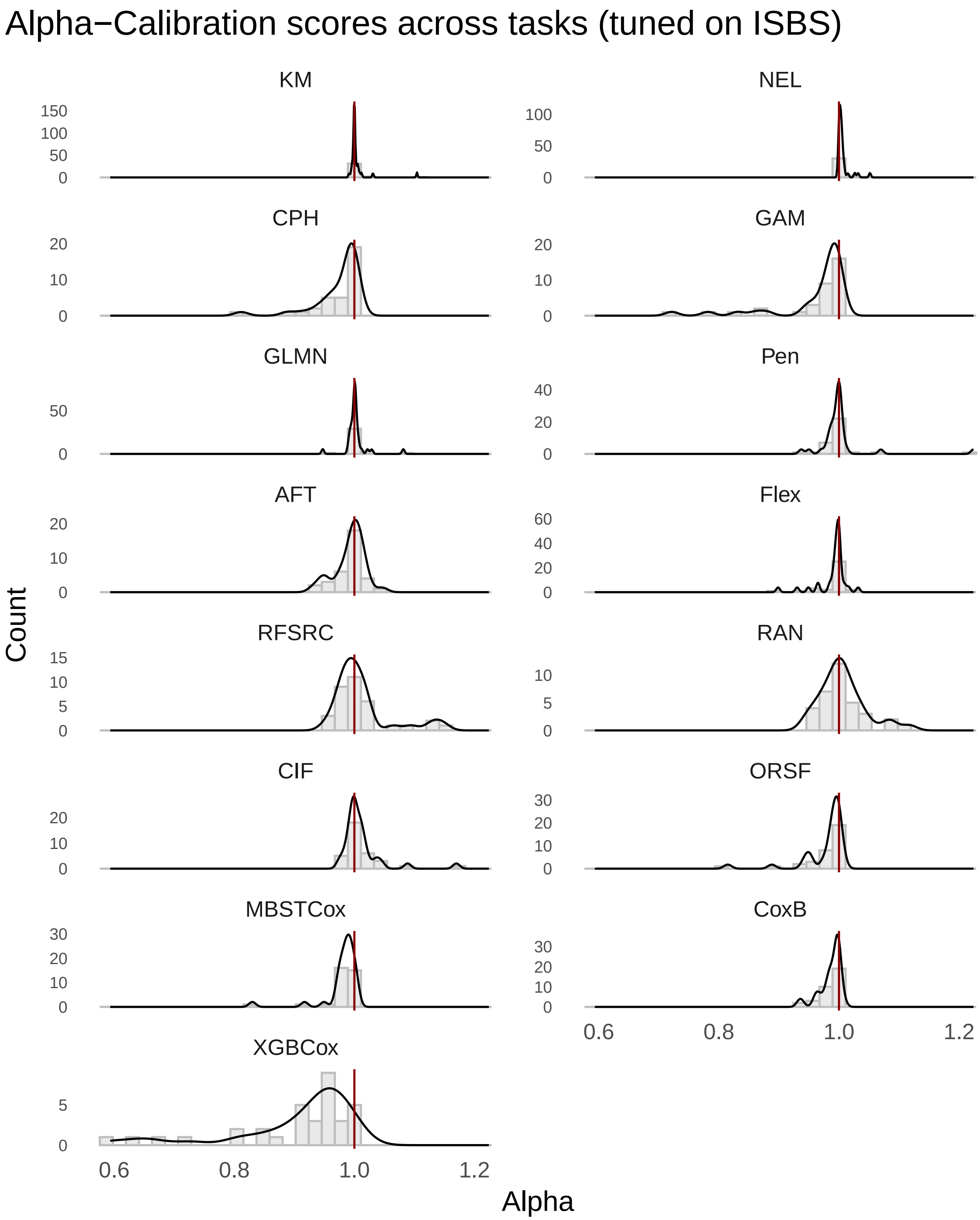

Figure 26: Calibration scores across all datasets for each model using van Houwelingen's α indicate good calibration when values are close to 1.

E.4 Sensitivity Analysis (Plackett-Luce Models)

Here we present detailed results for the Plackett-Luce (PL) sensitivity analysis as introduced in **??**. Per-iteration scores are first averaged per model-dataset combination and then ranked to obtain one complete ranking ("rater") per dataset over all models ("items"). KM and NEL baselines are excluded from all PL analyses as they are considered uninformative for model comparison and consume degrees of freedom, making model fitting more difficult. All analyses are run separately for both tuning measures (Harrell's C and ISBS).

E.4.1 Full Plackett-Luce Rankings

We first fit a single PL model on the full set of rankings across all datasets. Figures 27 and 28 show the estimated log-worth parameters with quasi-standard errors relative to CPH as the reference model, such that models to the right of the dashed line are estimated to rank higher than CPH on average.

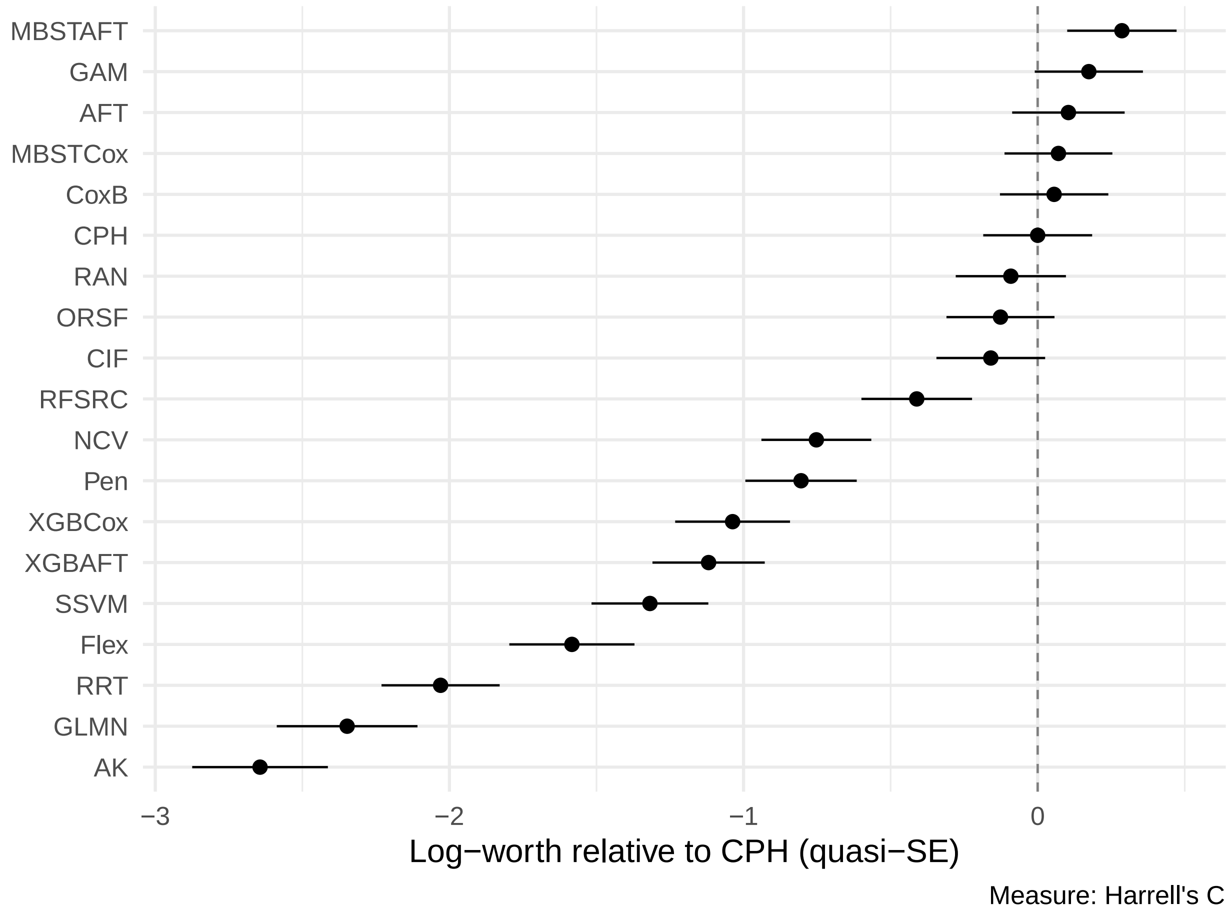

Figure 27: Full PL model log-worth parameters relative to CPH with quasi-standard errors, for models tuned and evaluated on Harrell's C.

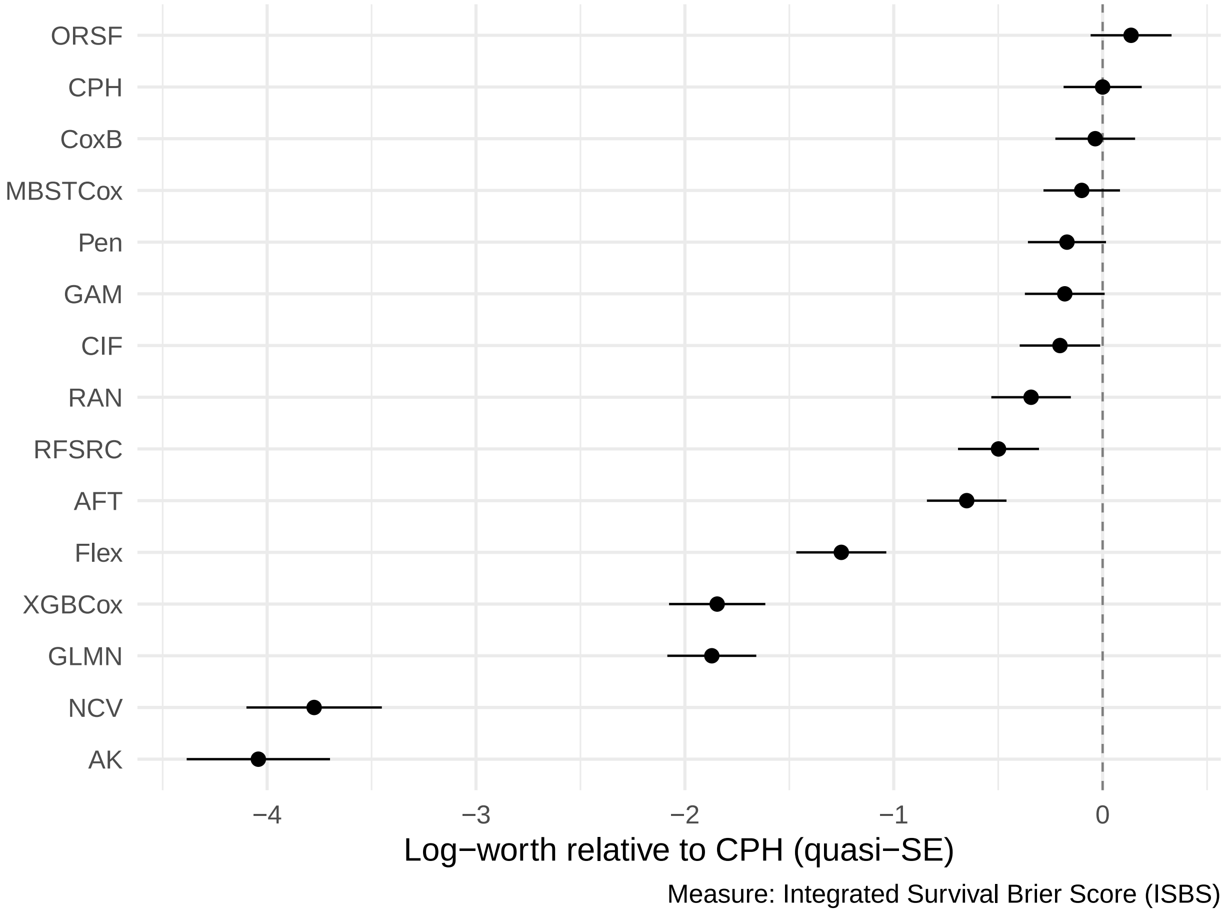

Figure 28: Full PL model log-worth parameters relative to CPH with quasi-standard errors, for models tuned and evaluated on ISBS.

E.4.2 Manual Subgroup Analysis

To investigate whether dataset characteristics influence model rankings, we fit separate PL models for subgroups based on four pre-defined dichotomization criteria:

1. **CPH misspecification**: Whether the global Grambsch-Therneau test [Grambsch and Therneau, 1994] indicates misspecification of the CPH model (uncorrected $p < 0.05$).
2. **Censoring proportion**: Split at the median censoring proportion (45.3%) across datasets.
3. **Sample size to feature ratio** ($\frac{n}{p}$): Split at the median $\frac{n}{p}$ ratio (189), as a proxy for dimensionality.
4. **Sample size**: Split at $N = 1000$.

For each split, we compare the combined log-likelihood of the two subgroup-specific PL models against the full (pooled) model using a likelihood ratio (LR) test. A significant LR test indicates that the ranking of models differs meaningfully between the two subgroups.

CPH Misspecification Figures 29 and 30 show the log-worth parameters for datasets where the CPH model is misspecified versus where it is not. The LR test is significant for both measures ($p < 0.01$), indicating that CPH misspecification status influences model rankings. For Harrell's C, CPH ranks best when the PH assumption is not violated, with CoxB close behind, while MBSTAFT leads when the assumption is violated, followed by MBSTCox and GAM. For ISBS, CoxB ranks best when the PH assumption holds, but when violated, CIF ranks highest with ORSF close behind.

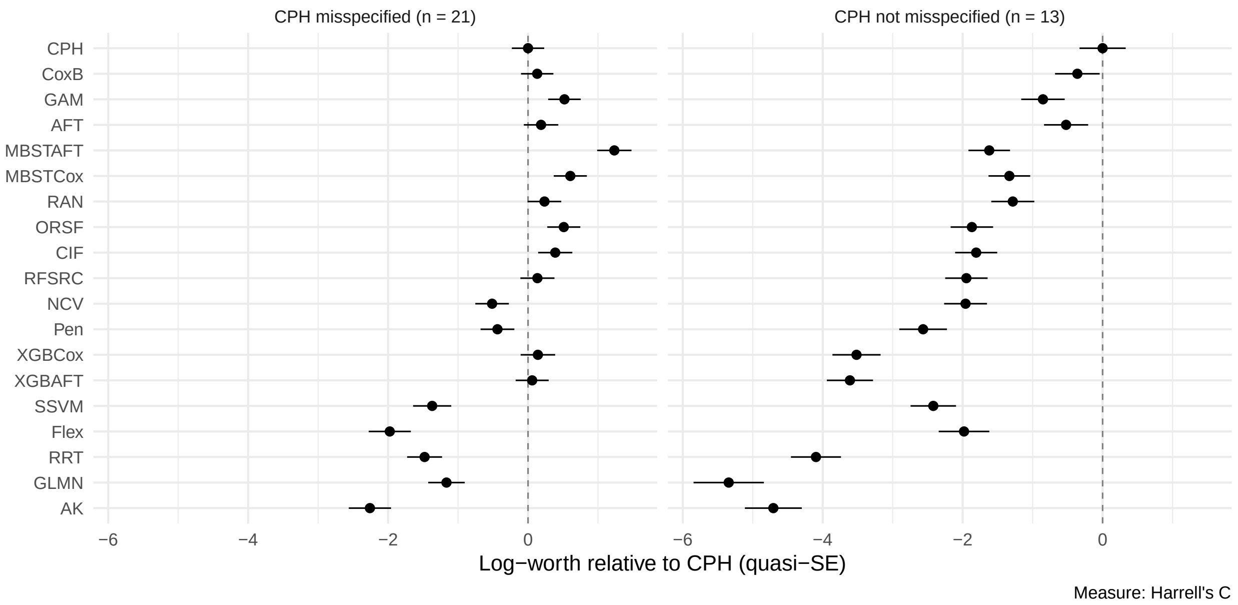

Figure 29: PL log-worth by CPH misspecification status, tuned and evaluated on Harrell's C. Log-worth is relative to CPH with quasi-standard errors.

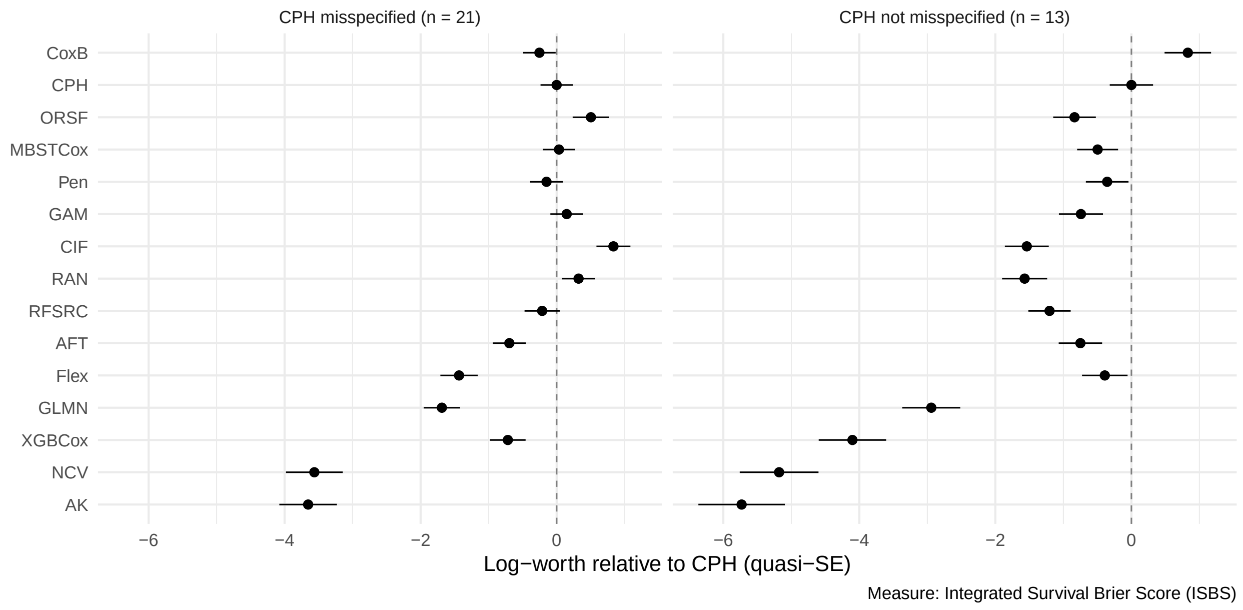

Figure 30: PL log-worth by CPH misspecification status, tuned and evaluated on ISBS.

Censoring Proportion Figures 31 and 32 show the log-worth parameters for datasets with low versus high censoring proportion. The LR test is significant for ISBS ($p < 0.01$) but not for Harrell's C ($p = 0.79$), suggesting that censoring proportion affects the ranking of distributional predictions but not discrimination. For Harrell's C, MBSTAFT ranks better than other models on low-censoring datasets, though error bars overlap with CPH and others. For ISBS, ORSF ranks considerably better on low-censoring than on high-censoring datasets, with ORSF and CIF performing well in the low-censoring subgroup.

Sample Size to Feature Ratio Figures 33 and 34 show the log-worth parameters for datasets with low versus high n/p ratio. The LR test is marginally significant for ISBS ($p < 0.05$) but not for Harrell's C ($p = 0.44$). For Harrell's C, MBSTAFT leads slightly in the high n/p subgroup, but overall the picture is similar across subgroups, with most well-performing learners showing comparable worth to CPH. For ISBS, there are barely any differences between the two subgroups.

Sample Size Figures 35 and 36 show the log-worth parameters for small ($n < 1000$) versus large ($n \geq 1000$) datasets. The LR test is significant for both Harrell's C and ISBS ($p < 0.001$), indicating that sample size influences model rankings for both discrimination and distributional predictions. In particular, it is apparent that more complex methods (MBSTAFT, GAM) tend to perform better on larger datasets compared to CPH, in particular in terms of discrimination.

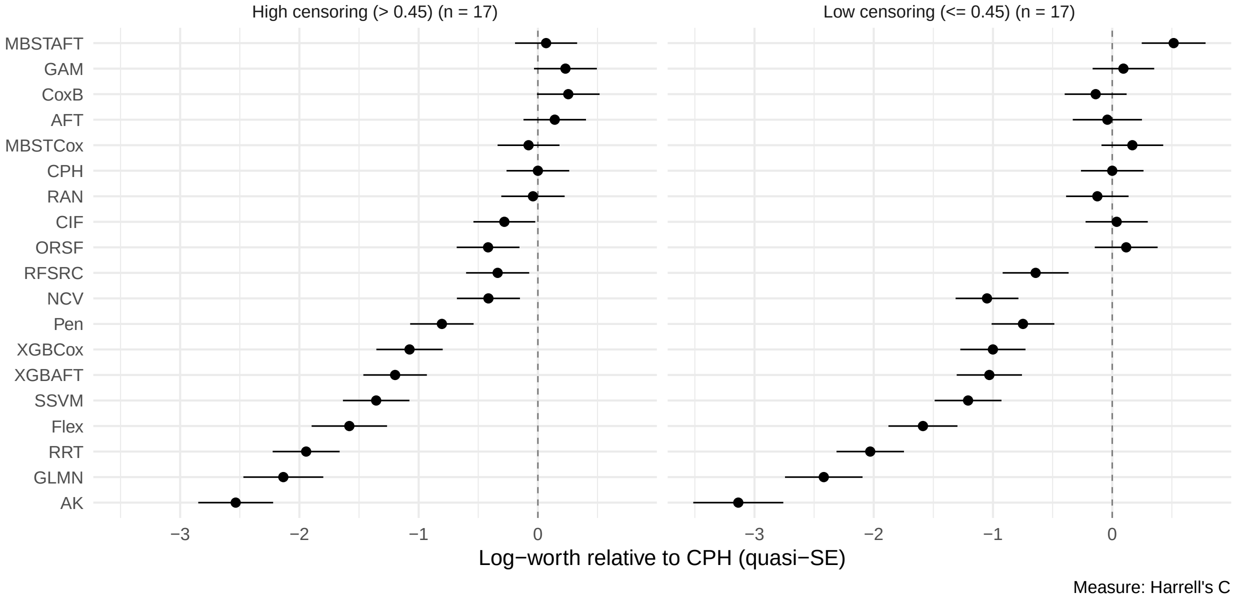

Figure 31: PL log-worth by censoring proportion, tuned and evaluated on Harrell's C. Log-worth is relative to CPH with quasi-standard errors.

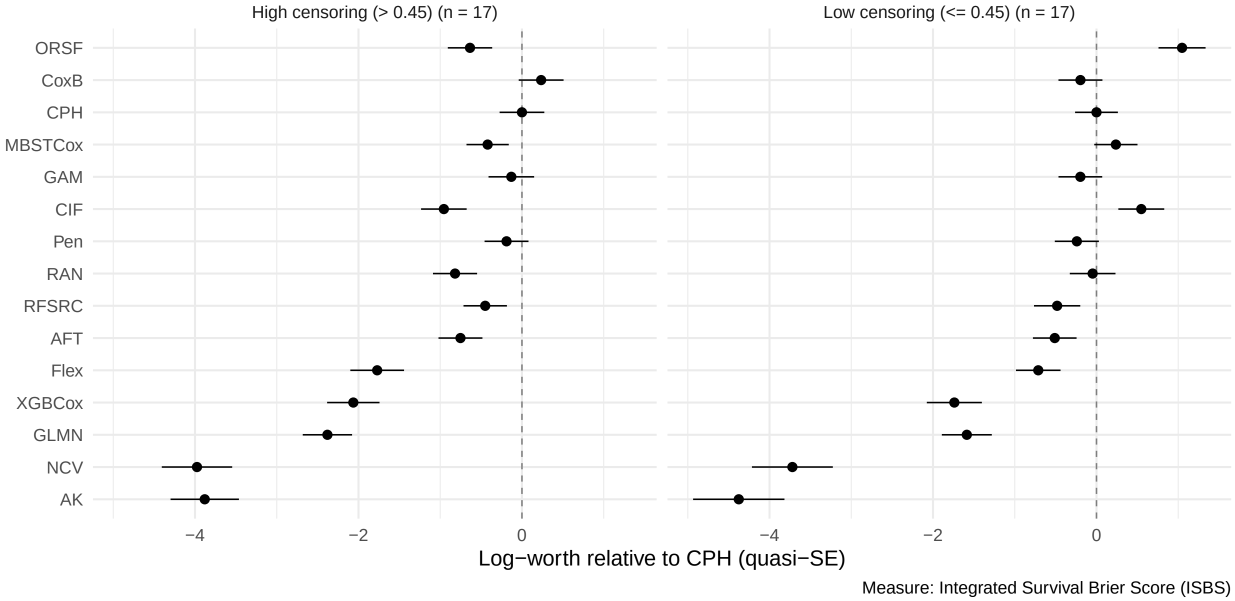

Figure 32: PL log-worth by censoring proportion, tuned and evaluated on ISBS.

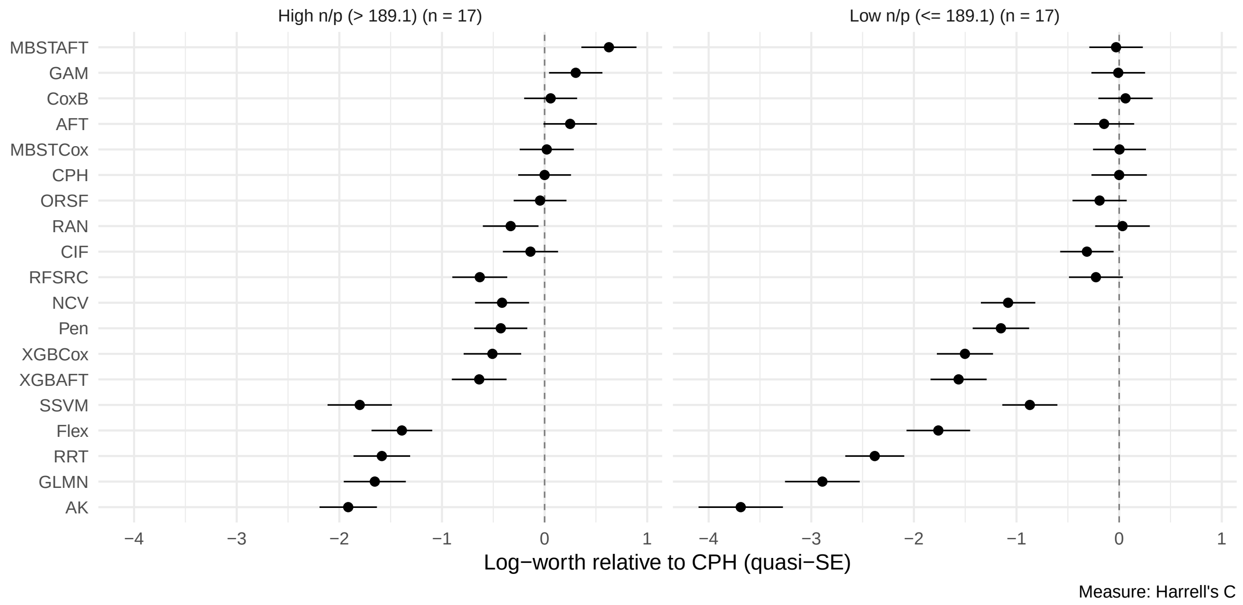

Figure 33: PL log-worth by n/p ratio, tuned and evaluated on Harrell's C. Log-worth is relative to CPH with quasi-standard errors.

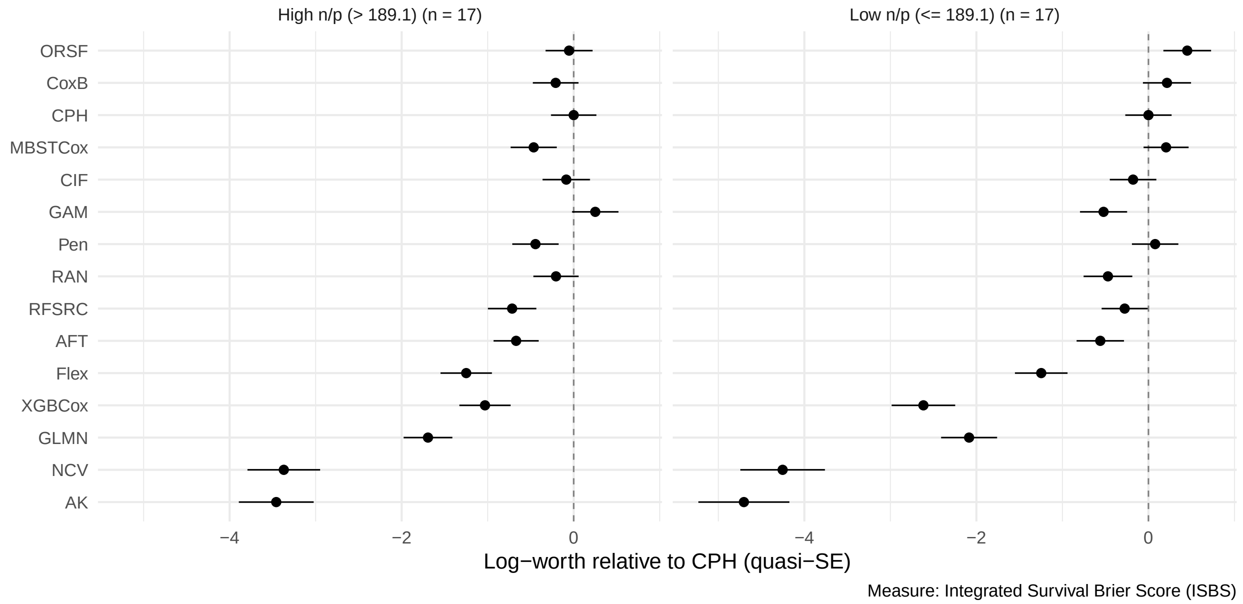

Figure 34: PL log-worth by n/p ratio, tuned and evaluated on ISBS.

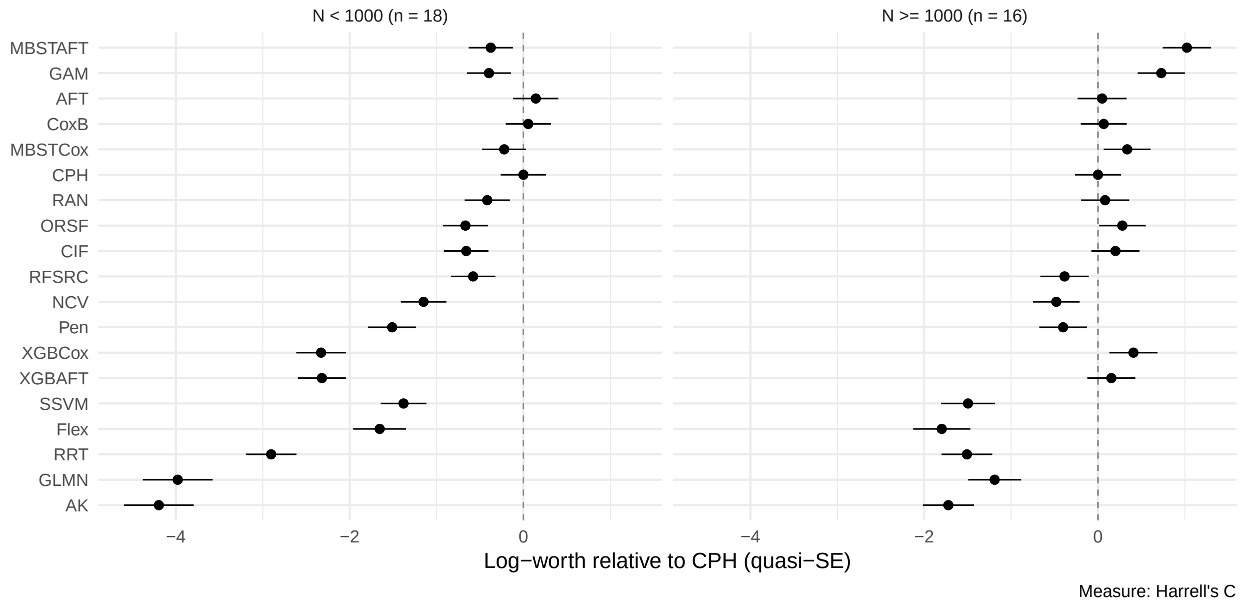

Figure 35: PL log-worth by sample size ($n < 1000$ vs. $n \geq 1000$), tuned and evaluated on Harrell's C. Log-worth is relative to CPH with quasi-standard errors.

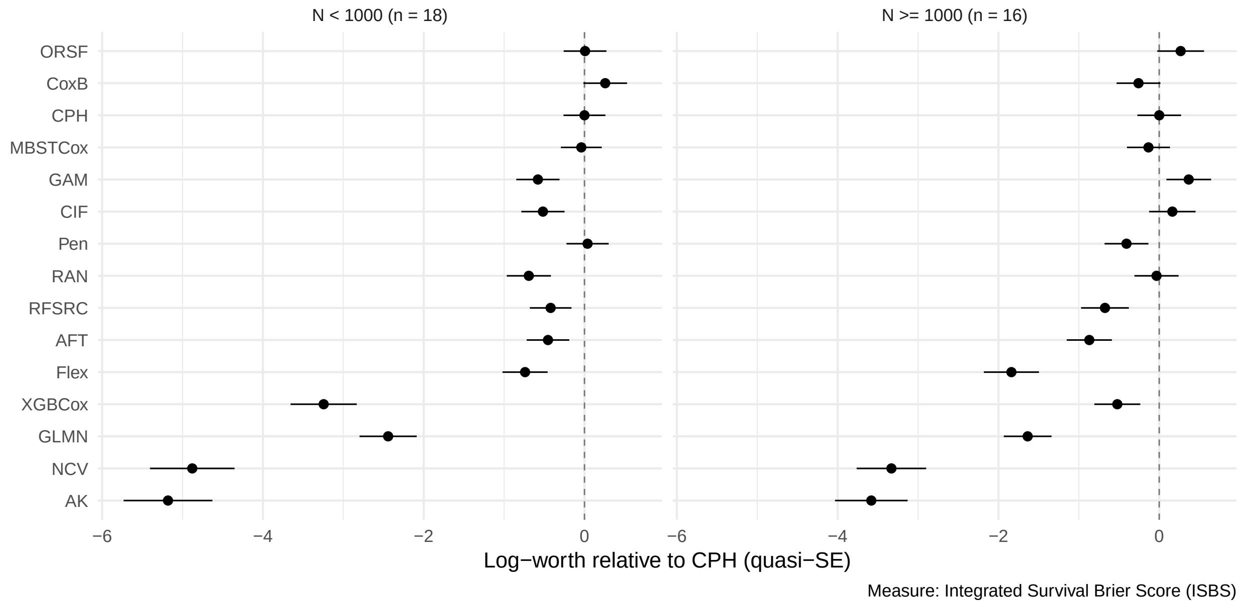

Figure 36: PL log-worth by sample size ($n < 1000$ vs. $n \geq 1000$), tuned and evaluated on ISBS.

E.4.3 Plackett-Luce Trees

As a data-driven alternative to the manual subgroup analysis, we use Plackett-Luce trees [Strobl et al., 2011], which use model-based recursive partitioning to automatically detect subgroups of datasets where model rankings differ significantly. The tree tests for parameter instability with respect to the covariates $\log(n/p)$, censoring proportion, and (binary) CPH misspecification status. A split is performed when a significant instability is detected, yielding terminal nodes with separate PL model fits and corresponding worth parameters.

We fit PL trees in two configurations:

- **Strict**: $\alpha = 0.1$ with Bonferroni correction across covariates, minimum node size of 10. This configuration controls the Type I error rate more conservatively.
- **Lenient**: $\alpha = 0.2$ with Bonferroni correction, minimum node size of 5. This configuration allows for detecting weaker signals at the cost of a higher risk of spurious splits.

Under the strict configuration, PL trees found no significant split for Harrell's C, while ISBS yielded a split (Figure 37). Under the lenient configuration, both measures produced splits (Figures 38 and 39). The resulting trees were identical also when $\log \frac{n}{p}$ was substituted with n. Worth parameters displayed in the terminal nodes indicate the estimated model ranking within each subgroup, with taller bars corresponding to higher-ranked models.

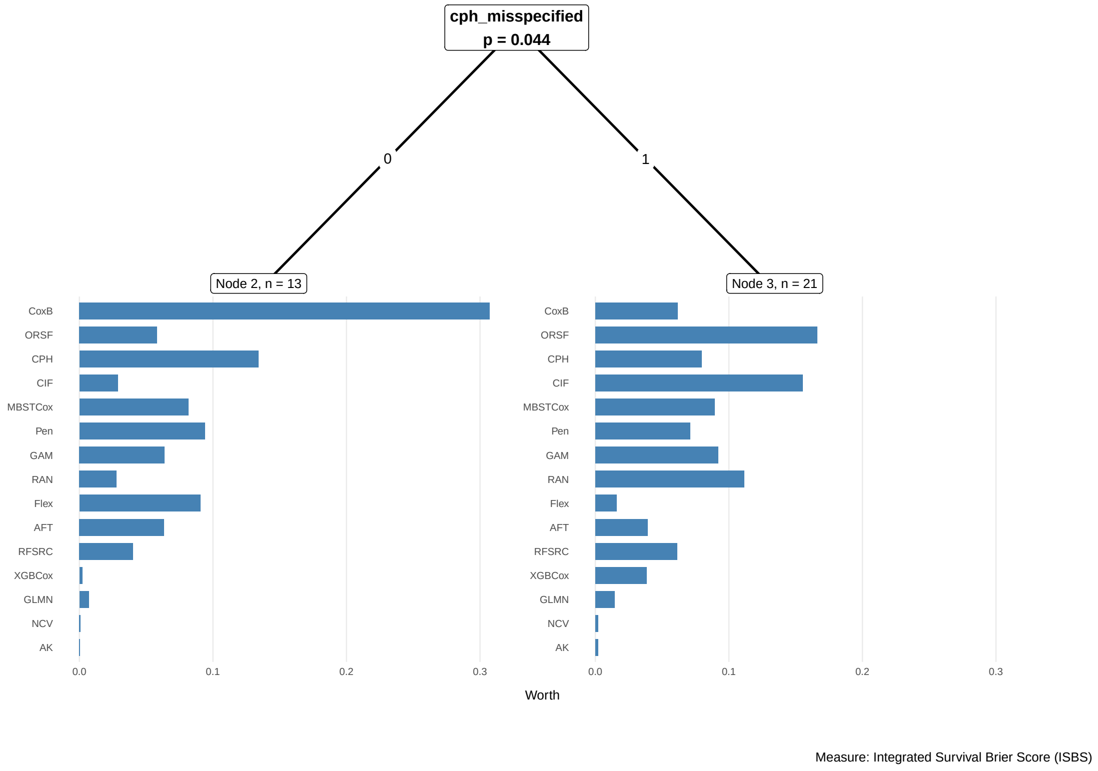

Figure 37: PL tree for ISBS under the strict configuration ($\alpha = 0.1$, Bonferroni-corrected, minimum node size 10).

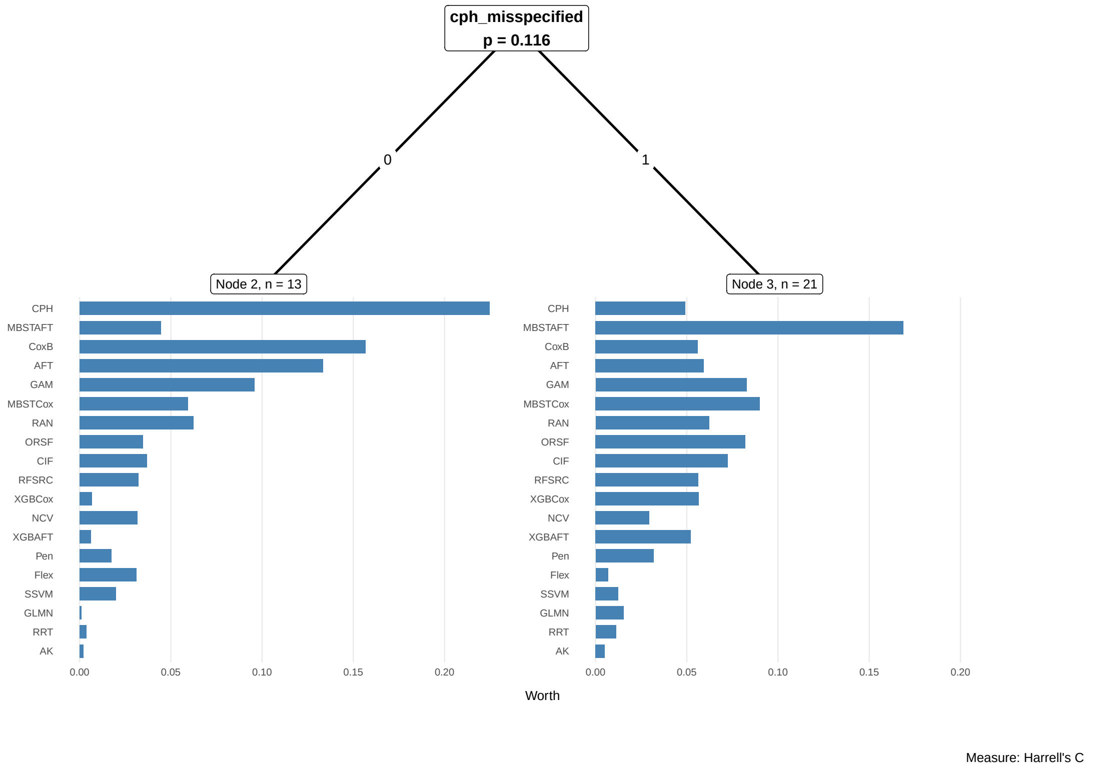

Figure 38: PL tree for Harrell's C under the lenient configuration ($\alpha = 0.2$, Bonferroni-corrected, minimum node size 5).

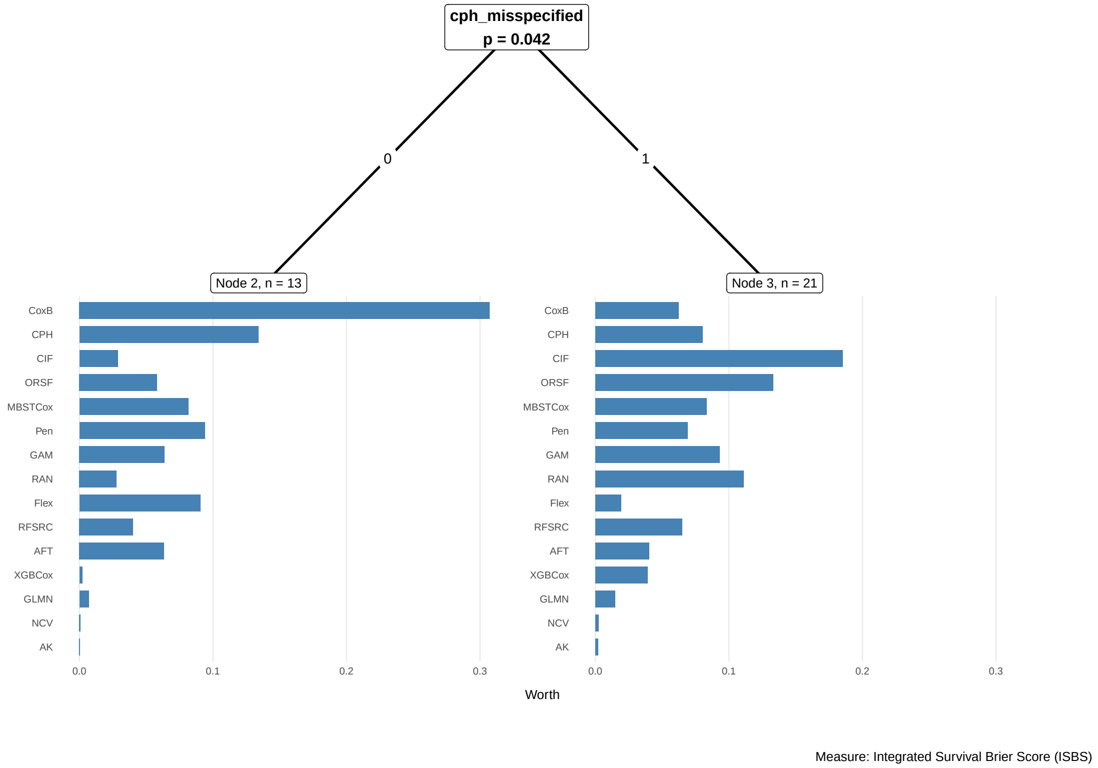

Figure 39: PL tree for ISBS under the lenient configuration ($\alpha = 0.2$, Bonferroni-corrected, minimum node size 5).

E.5 Errors

During the many computational steps performed during this benchmark, software errors are inevitably bound to happen. As we noted in **??**, we impute missing evaluation scores in resampling folds using the score of the Kaplan-Meier estimator, which affects the results presented in this paper.

Table 6 counts the number of errors encountered for each model, dataset, and tuning measures used in the benchmark per outer resampling iteration (up to 30). We note that particularly the tasks hdfail and child have caused the majority of the runtime- or memory-related errors here due to their large sample sizes and number of unique time points.

Table 6: Number of errors per outer resampling iteration (up to 30), separated by model, dataset, and tuning measure.

Model	Dataset	Harrell's C	ISBS	Total Errors
AK	CarpenterFdaData	0 (0%)	1 (3.3%)	1
AK	channing	1 (3.3%)	1 (3.3%)	2
AK	child	3 (100%)	3 (100%)	6
AK	e1684	3 (10%)	0 (0%)	3
AK	hdfail	3 (100%)	3 (100%)	6
AK	lung	8 (26.7%)	0 (0%)	8
AK	uis	2 (6.7%)	0 (0%)	2
AK	veteran	3 (10%)	0 (0%)	3
GLMN	bladder0	1 (3.3%)	0 (0%)	1
GLMN	channing	0 (0%)	1 (3.3%)	1
GLMN	check_times	2 (66.7%)	0 (0%)	2
GLMN	cost	12 (40%)	0 (0%)	12
GLMN	dataSTR	2 (6.7%)	0 (0%)	2
GLMN	hdfail	0 (0%)	3 (100%)	3
GLMN	std	6 (20%)	0 (0%)	6
GLMN	uis	4 (13.3%)	0 (0%)	4
GLMN	veteran	0 (0%)	14 (46.7%)	14
GLMN	wbc1	0 (0%)	4 (13.3%)	4
Pen	aids.id	1 (3.3%)	9 (30%)	10
Pen	bladder0	8 (26.7%)	0 (0%)	8
Pen	channing	1 (3.3%)	0 (0%)	1
Pen	check_times	3 (100%)	3 (100%)	6
Pen	cost	3 (10%)	0 (0%)	3
Pen	dataSTR	11 (36.7%)	3 (10%)	14
Pen	hdfail	0 (0%)	2 (66.7%)	2
Flex	aids.id	0 (0%)	10 (33.3%)	10
Flex	check_times	3 (100%)	3 (100%)	6
Flex	child	3 (100%)	3 (100%)	6
Flex	dataFTR	2 (6.7%)	0 (0%)	2
Flex	hdfail	3 (100%)	3 (100%)	6
Flex	lung	9 (30%)	0 (0%)	9
Flex	nafld1	14 (93.3%)	14 (93.3%)	28
Flex	nwtco	15 (100%)	15 (100%)	30

Flex	support	3 (100%)	3 (100%)	6
Flex	wa_churn	15 (100%)	15 (100%)	30
RFSRC	check_times	3 (100%)	3 (100%)	6
RFSRC	child	3 (100%)	3 (100%)	6
RFSRC	colrec	1 (33.3%)	2 (66.7%)	3
RFSRC	nafld1	2 (13.3%)	1 (6.7%)	3
RFSRC	support	2 (66.7%)	3 (100%)	5
RAN	check_times	3 (100%)	2 (66.7%)	5
RAN	child	3 (100%)	3 (100%)	6
RAN	cost	1 (3.3%)	0 (0%)	1
RAN	hdfail	1 (33.3%)	1 (33.3%)	2
RAN	mgus	0 (0%)	2 (6.7%)	2
RAN	nafld1	4 (26.7%)	9 (60%)	13
CIF	child	3 (100%)	3 (100%)	6
CIF	hdfail	3 (100%)	3 (100%)	6
ORSF	child	3 (100%)	3 (100%)	6
ORSF	cost	1 (3.3%)	0 (0%)	1
ORSF	gbsg	1 (6.7%)	0 (0%)	1
ORSF	hdfail	3 (100%)	3 (100%)	6
ORSF	nafld1	1 (6.7%)	9 (60%)	10
ORSF	uis	0 (0%)	1 (3.3%)	1
ORSF	veteran	1 (3.3%)	0 (0%)	1
RRT	dataFTR	5 (16.7%)	0 (0%)	5
RRT	lung	8 (26.7%)	0 (0%)	8
RRT	metabric	7 (46.7%)	0 (0%)	7
RRT	nwtco	7 (46.7%)	0 (0%)	7
RRT	ova	3 (10%)	0 (0%)	3
RRT	tumor	3 (10%)	0 (0%)	3
MBSTCox	child	3 (100%)	3 (100%)	6
MBSTCox	dataSTR	0 (0%)	1 (3.3%)	1
MBSTCox	hdfail	3 (100%)	3 (100%)	6
MBSTAFT	hdfail	2 (66.7%)	0 (0%)	2
SSVM	check_times	3 (100%)	0 (0%)	3
SSVM	child	3 (100%)	0 (0%)	3
SSVM	colrec	3 (100%)	0 (0%)	3
SSVM	flchain	11 (73.3%)	0 (0%)	11
SSVM	hdfail	3 (100%)	0 (0%)	3
SSVM	nafld1	15 (100%)	0 (0%)	15
SSVM	nwtco	8 (53.3%)	0 (0%)	8
SSVM	ova	3 (10%)	0 (0%)	3
SSVM	support	3 (100%)	0 (0%)	3
SSVM	wa_churn	15 (100%)	0 (0%)	15

F Results per Dataset

For completeness, we display boxplots and violin plots of the evaluation scores across the outer resampling iterations per dataset, evaluation measure and tuning measure. Figures are shown grouped by model family and by model hypothesis space (see also Table 4).

F.1 Grouped by Learner Family

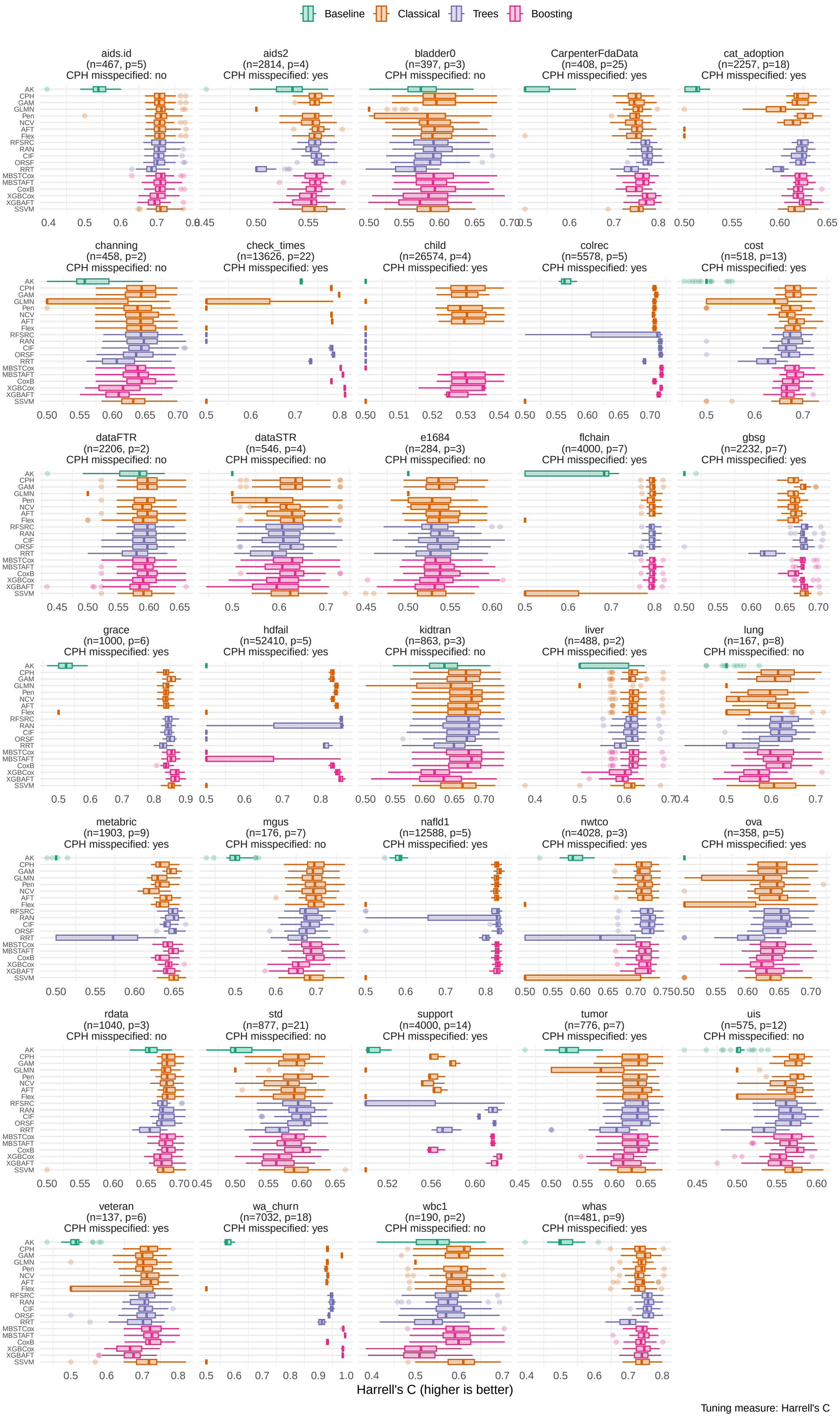

Figure 40: Per-dataset scores grouped by learner family for learners tuned and evaluated with Harrell's C

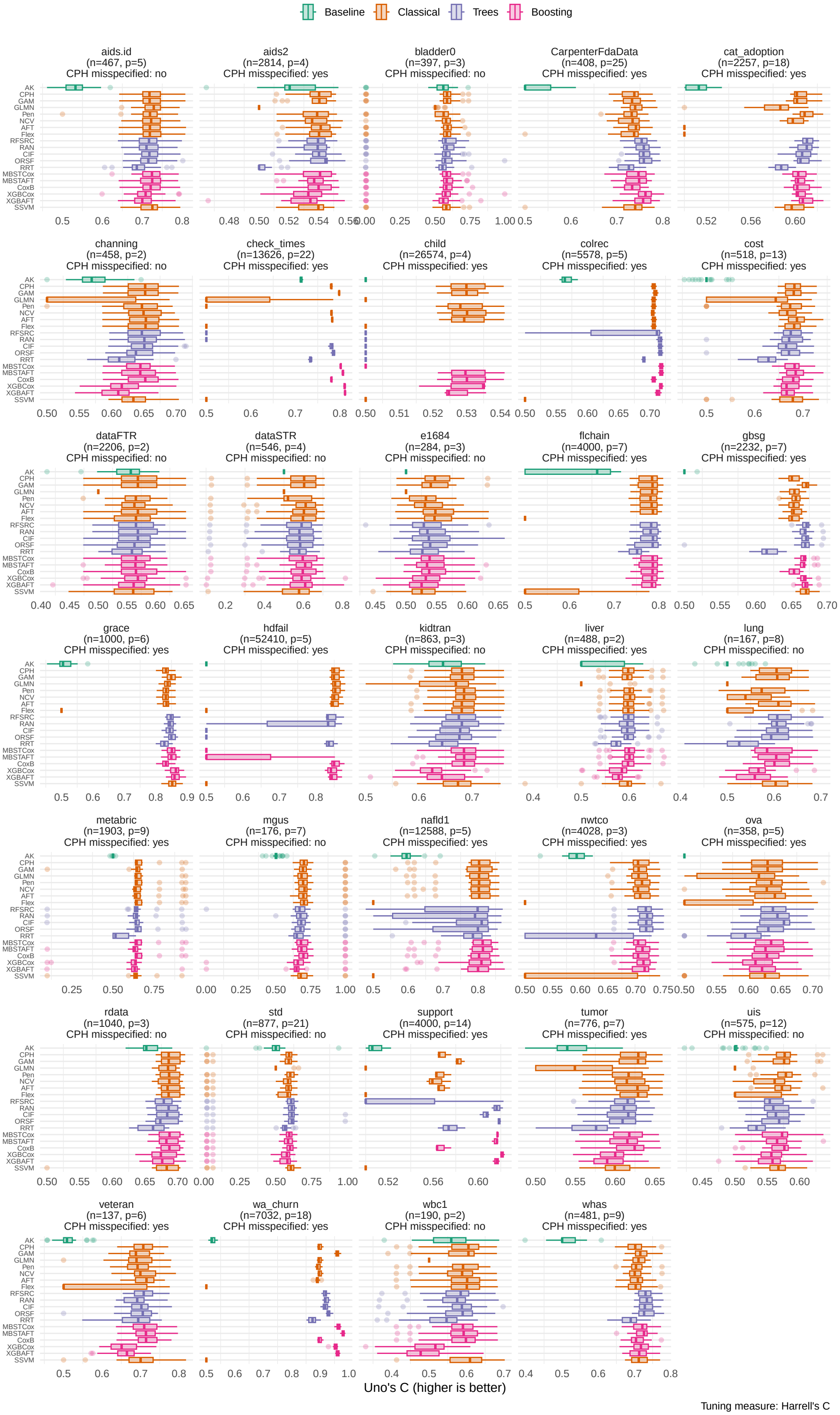

Figure 41: Per-dataset scores grouped by learner family for learners tuned on Harrell's C and evaluated with Uno's C

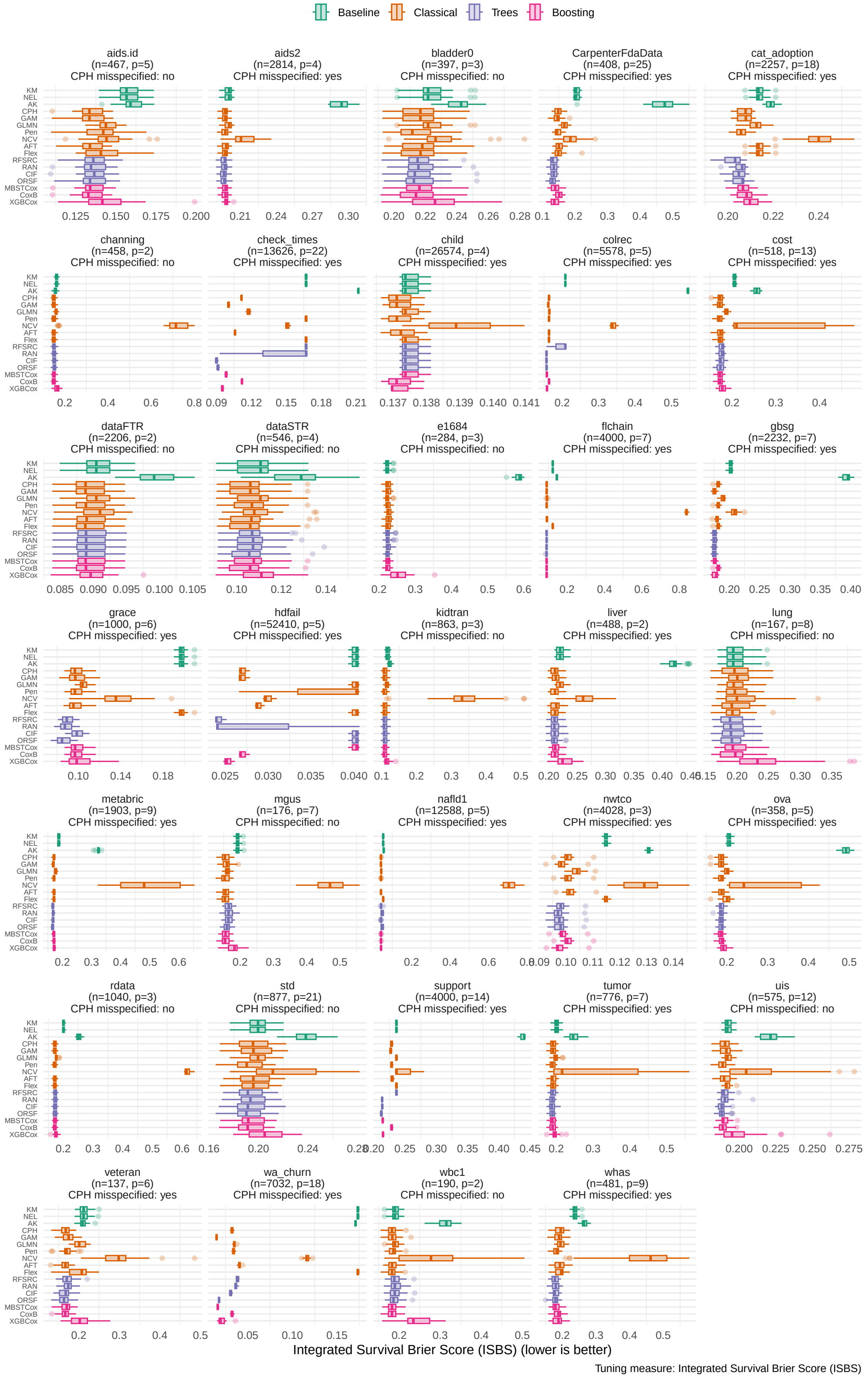

Figure 42: Per-dataset scores grouped by learner family for learners tuned and evaluated with ISBS

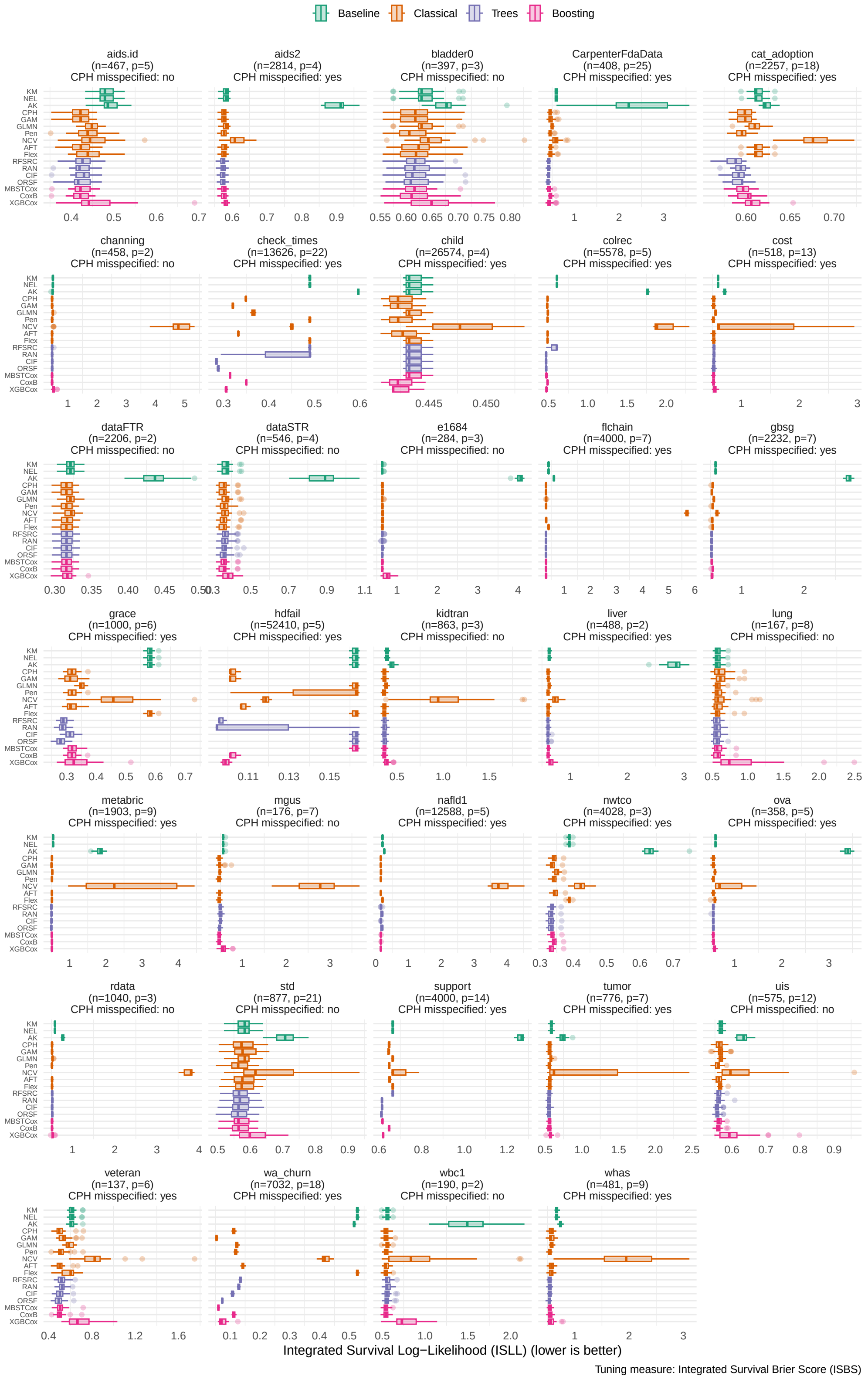

Figure 43: Per-dataset scores grouped by learner family for learners tuned on ISBS and evaluated with ISLL

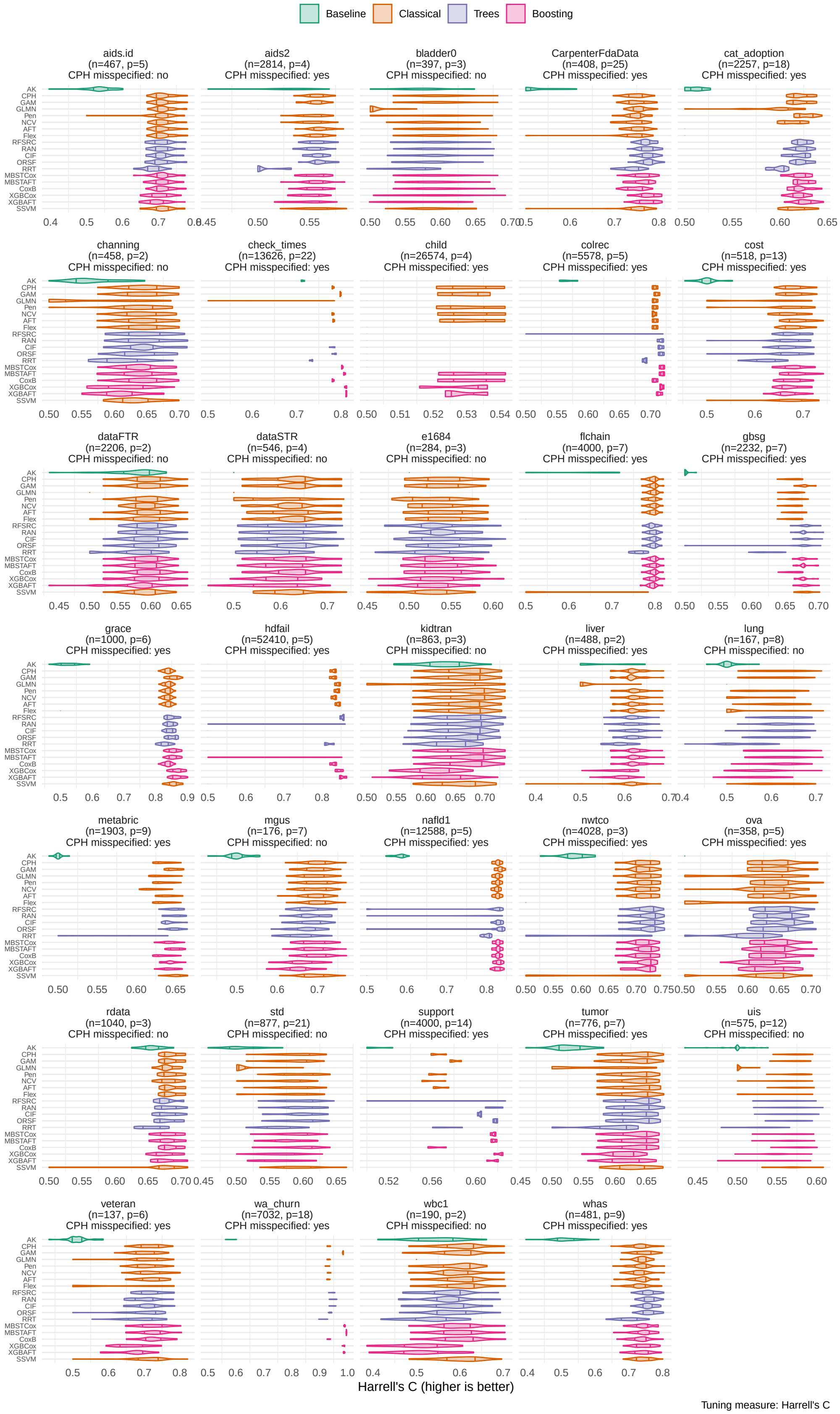

Figure 44: Per-dataset scores grouped by learner family for learners tuned and evaluated with Harrell's C (violin plot)

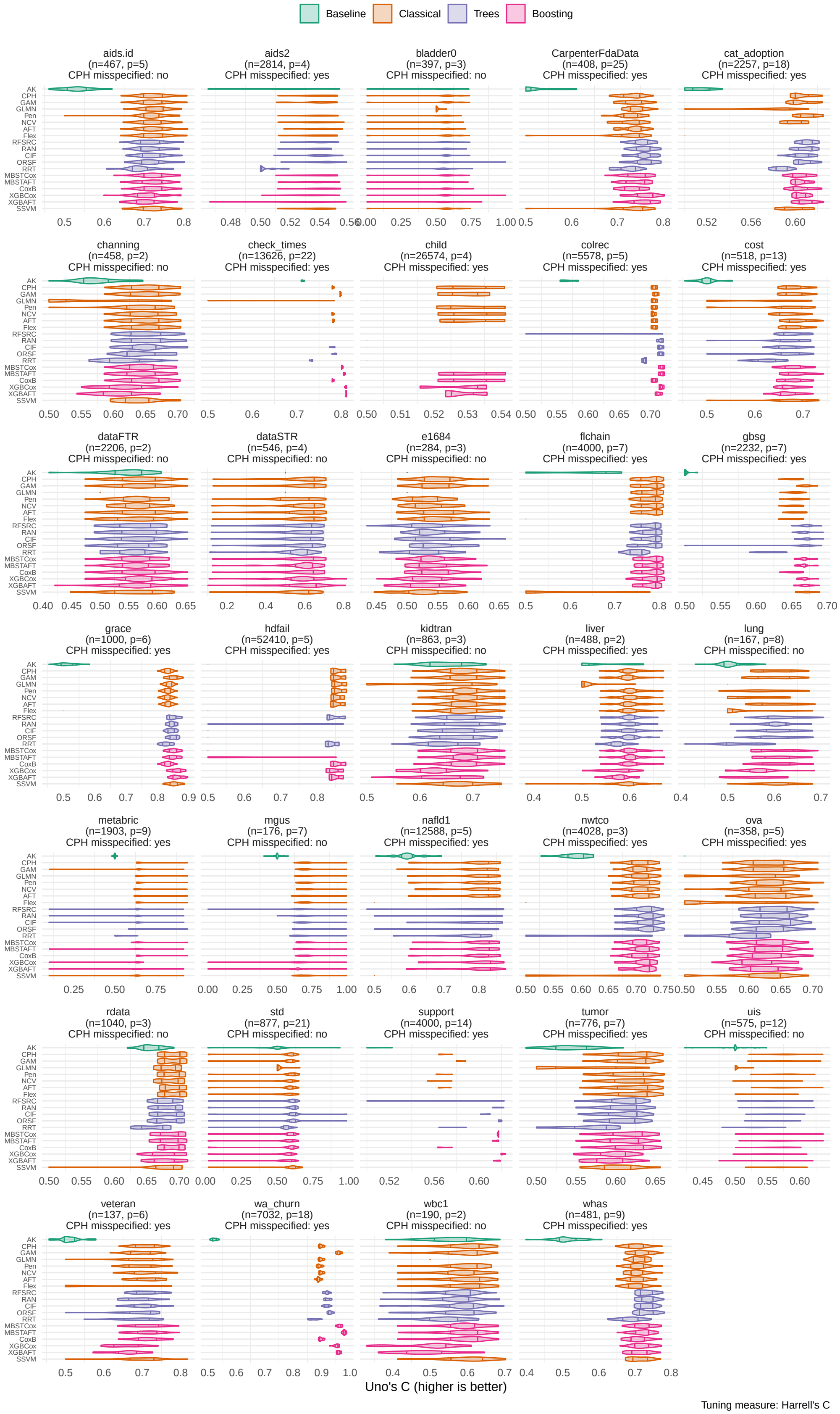

Figure 45: Per-dataset scores grouped by learner family for learners tuned on Harrell's C and evaluated with Uno's C (violin plot)

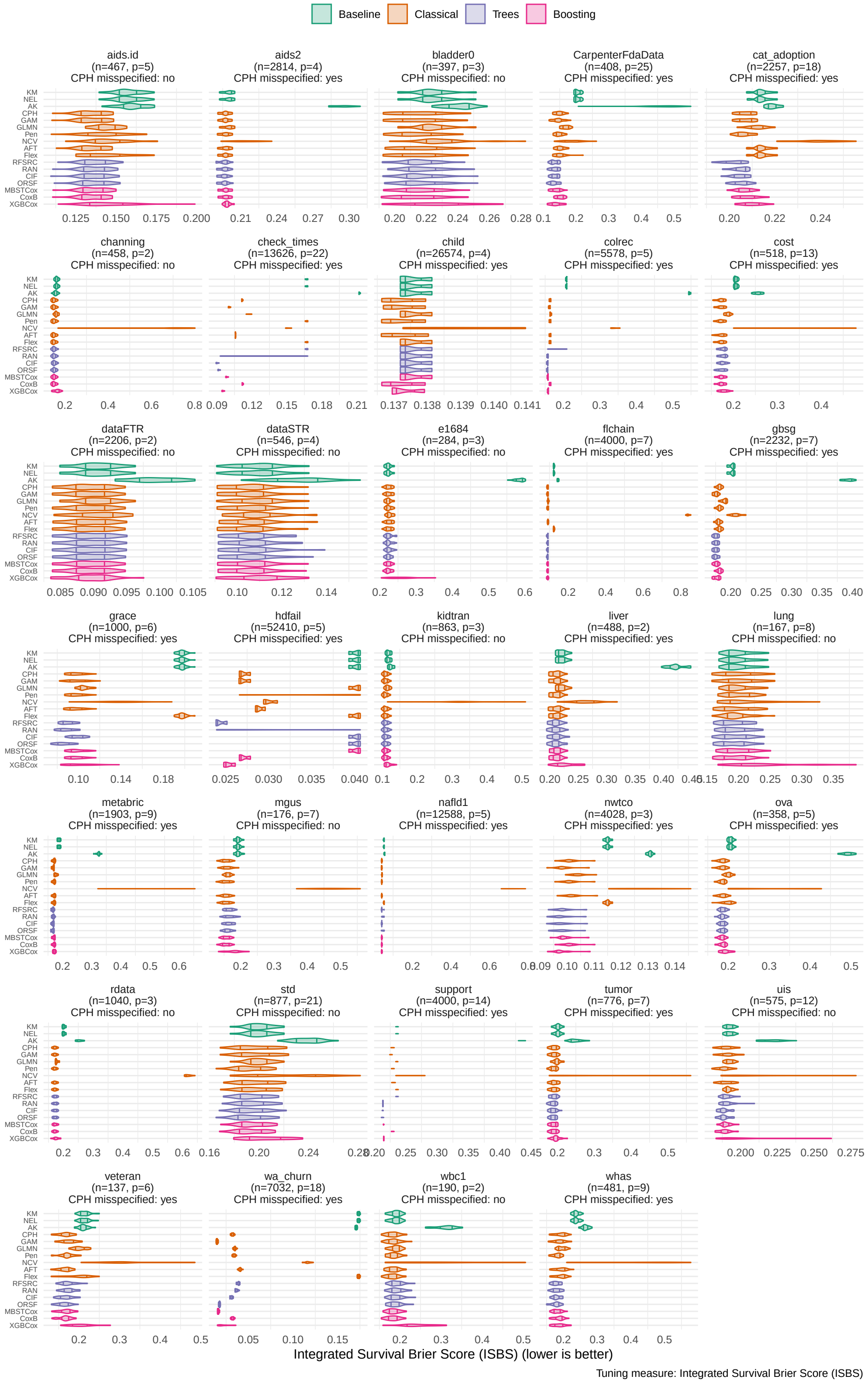

Figure 46: Per-dataset scores grouped by learner family for learners tuned and evaluated with ISBS (violin plot)

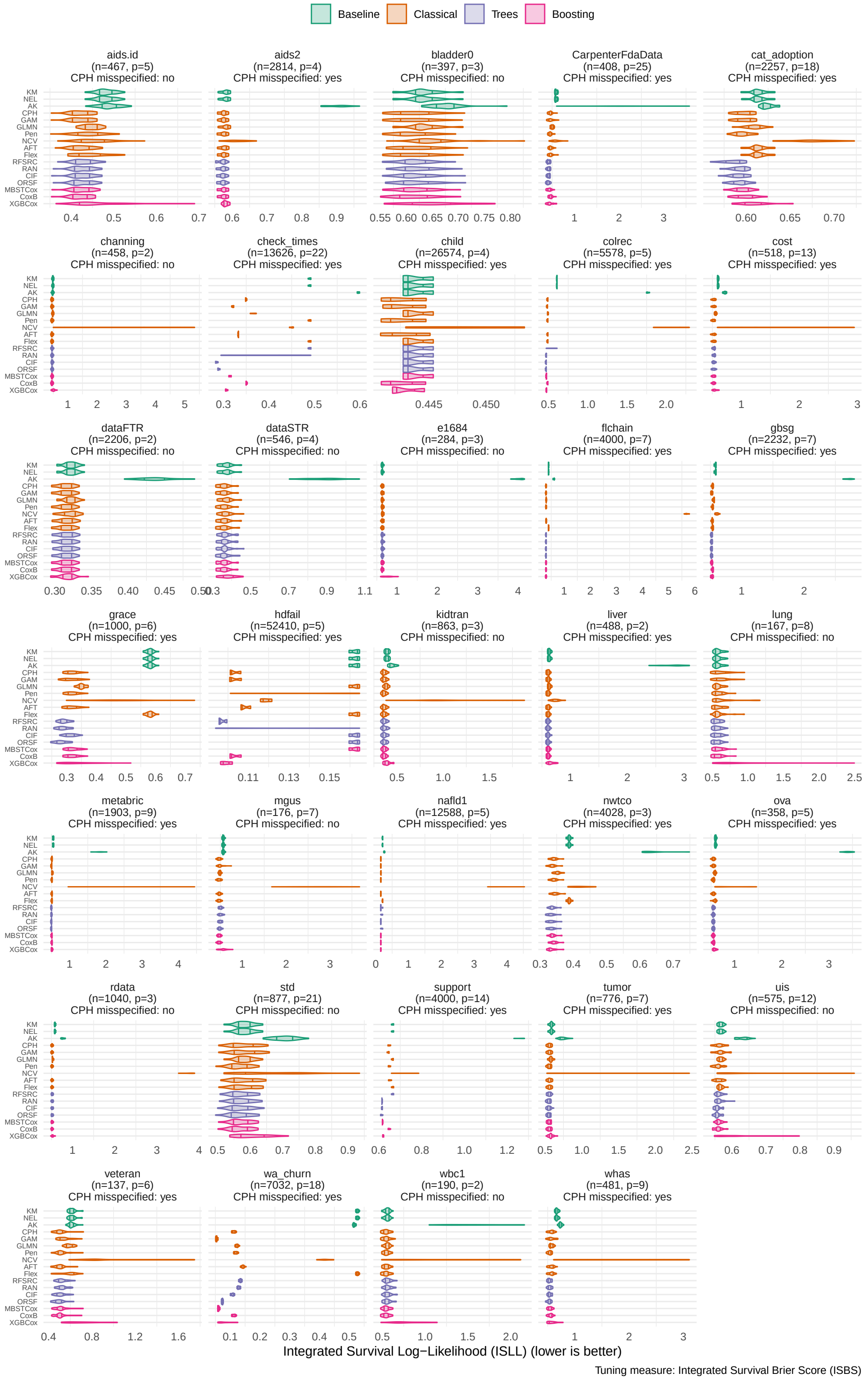

Figure 47: Per-dataset scores grouped by learner family for learners tuned on ISBS and evaluated with ISLL (violin plot)

F.2 Grouped by Learner Hypothesis Space

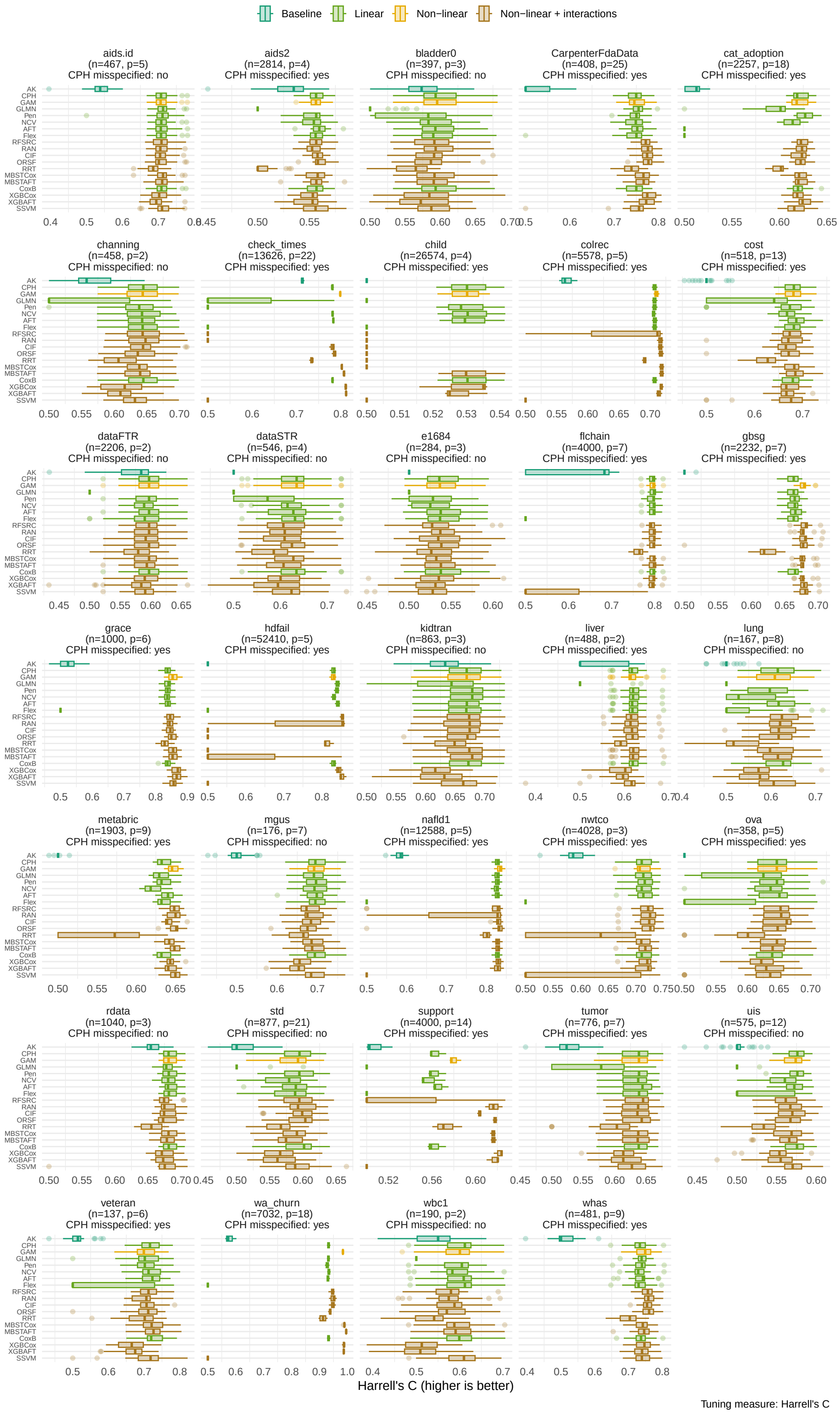

Figure 48: Per-dataset scores grouped by hypothesis space for learners tuned and evaluated with Harrell's C

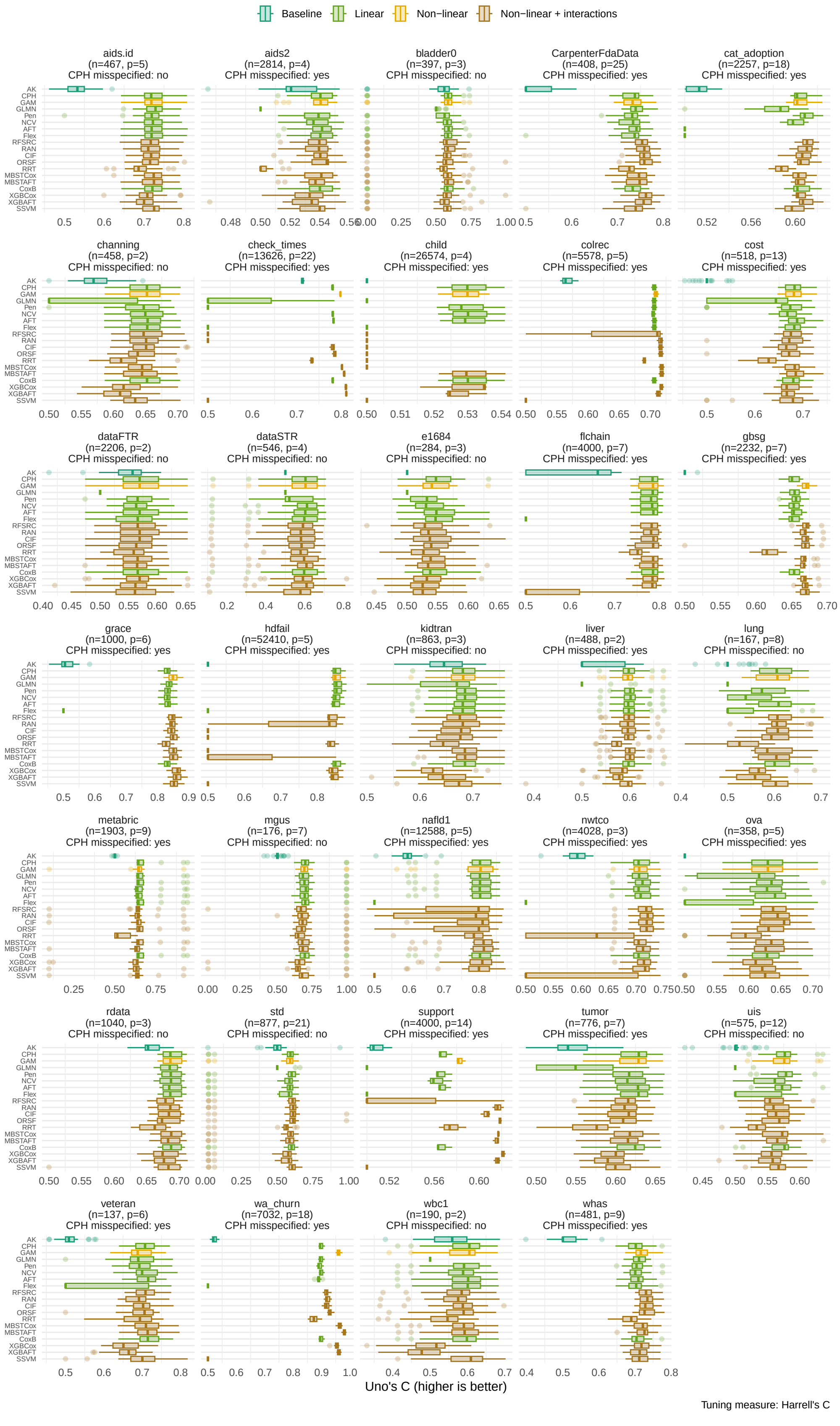

Figure 49: Per-dataset scores grouped by hypothesis space for learners tuned on Harrell's C and evaluated with Uno's C

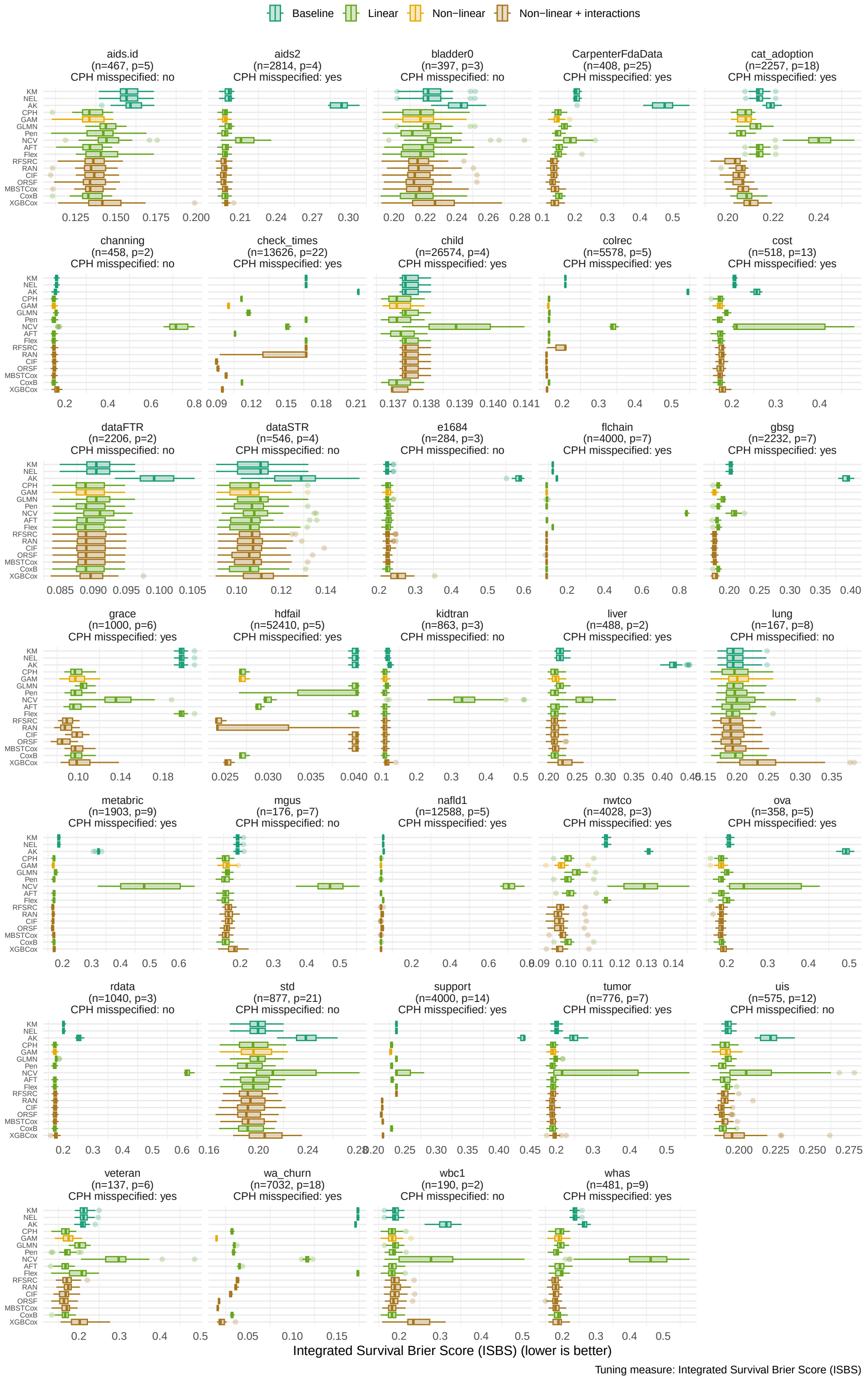

Figure 50: Per-dataset scores grouped by hypothesis space for learners tuned and evaluated with ISBS

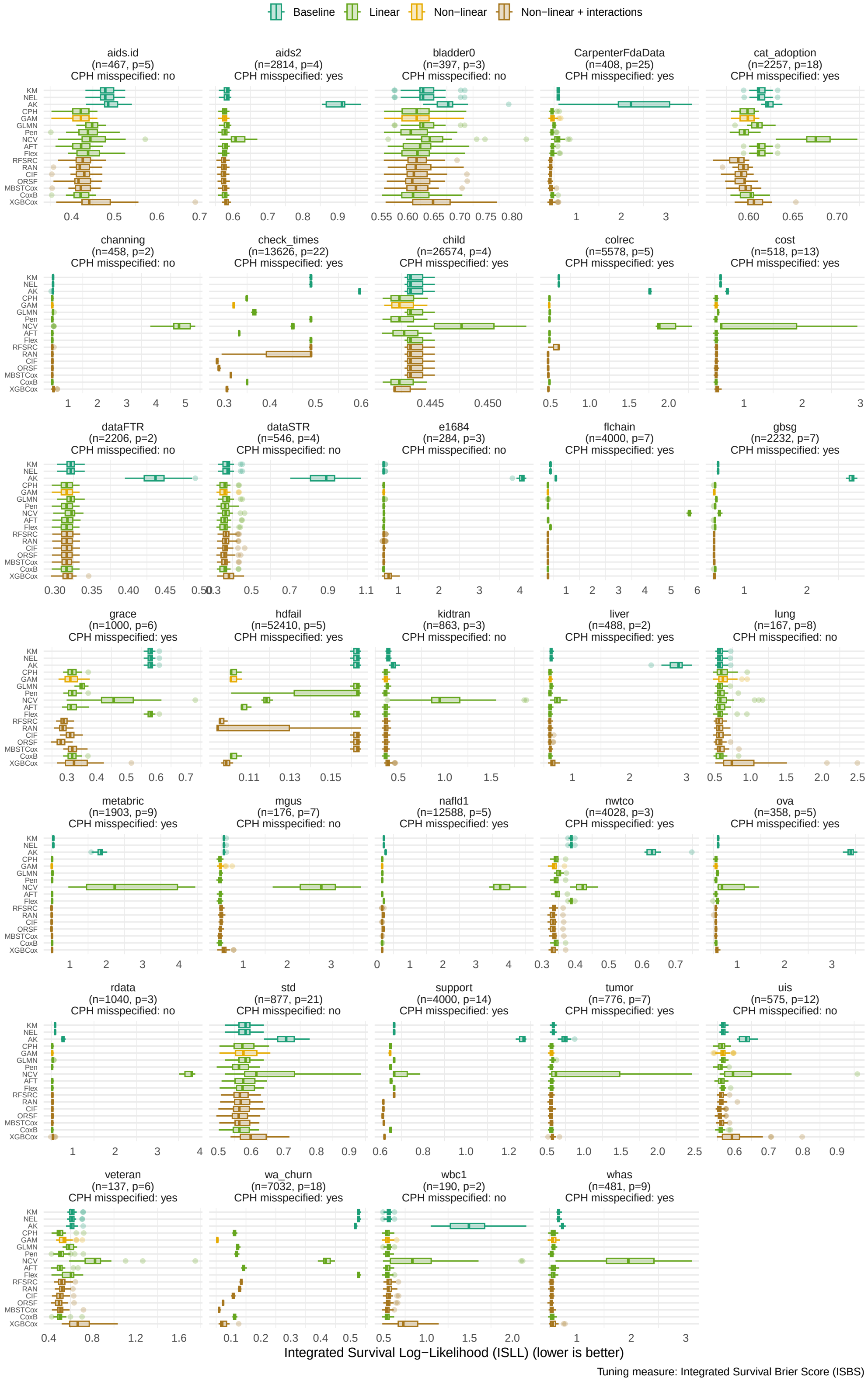

Figure 51: Per-dataset scores grouped by hypothesis space for learners tuned on ISBS and evaluated with ISLL

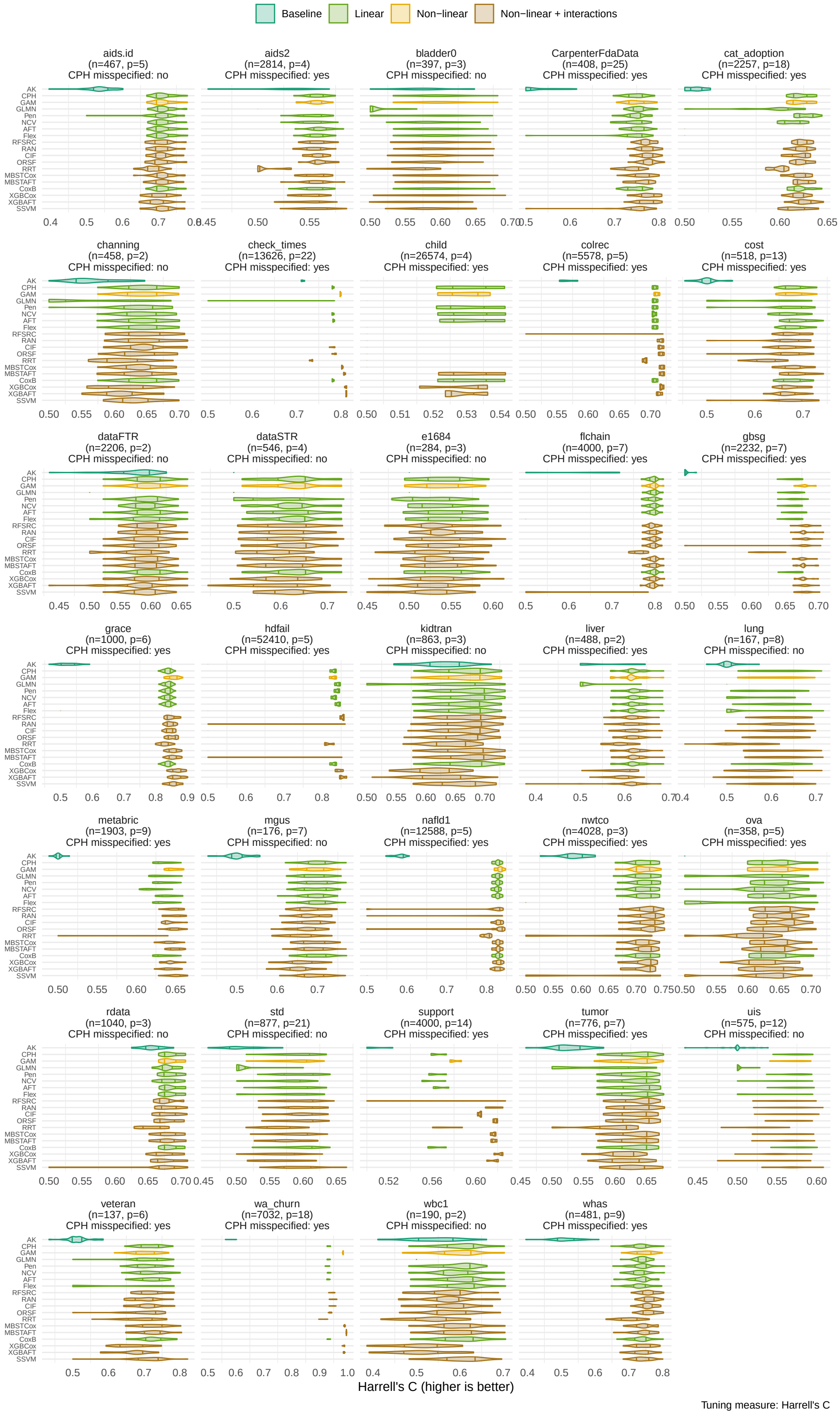

Figure 52: Per-dataset scores grouped by hypothesis space for learners tuned and evaluated with Harrell's C (violin plot)

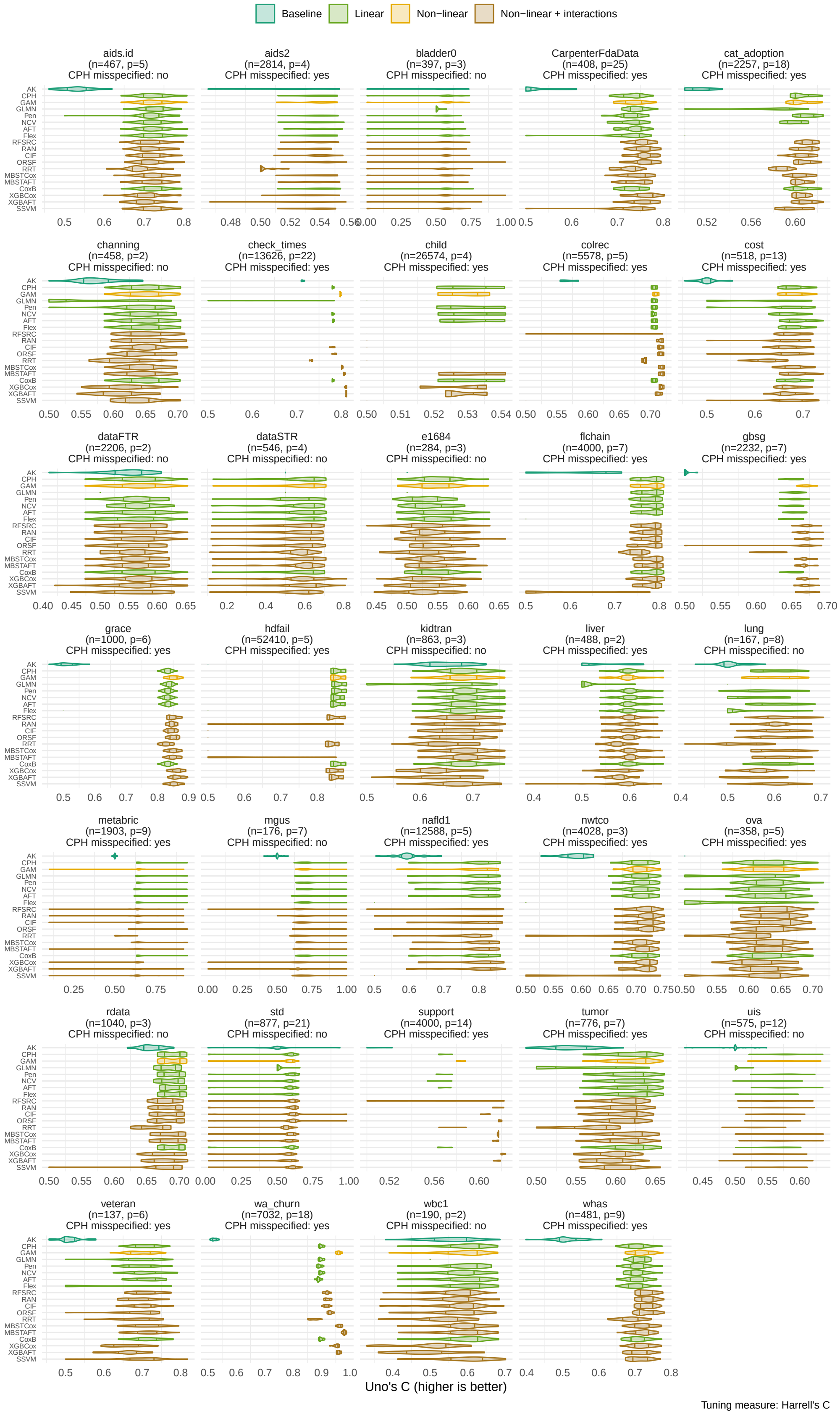

Figure 53: Per-dataset scores grouped by hypothesis space for learners tuned on Harrell's C and evaluated with Uno's C (violin plot)

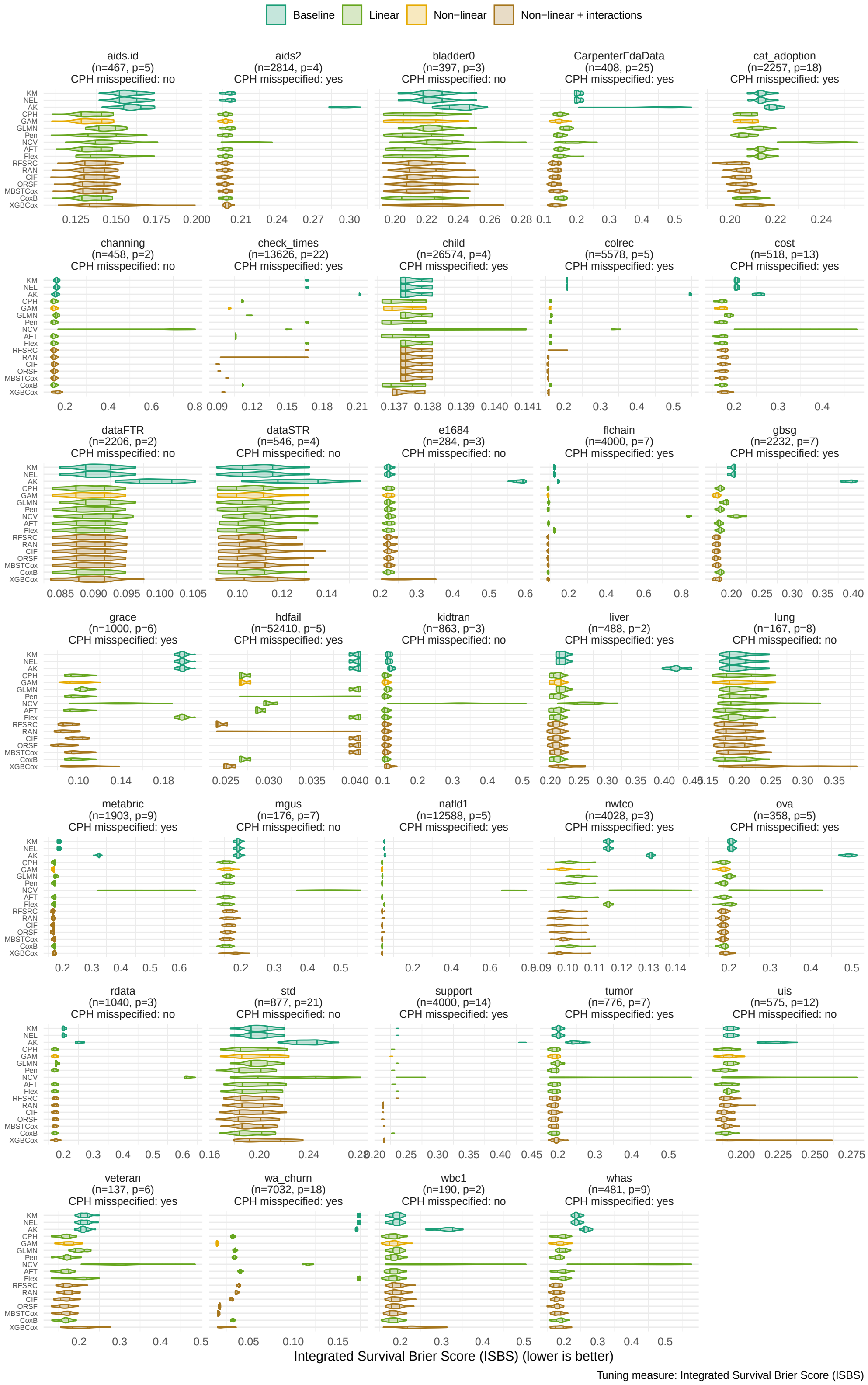

Figure 54: Per-dataset scores grouped by hypothesis space for learners tuned and evaluated with ISBS (violin plot)

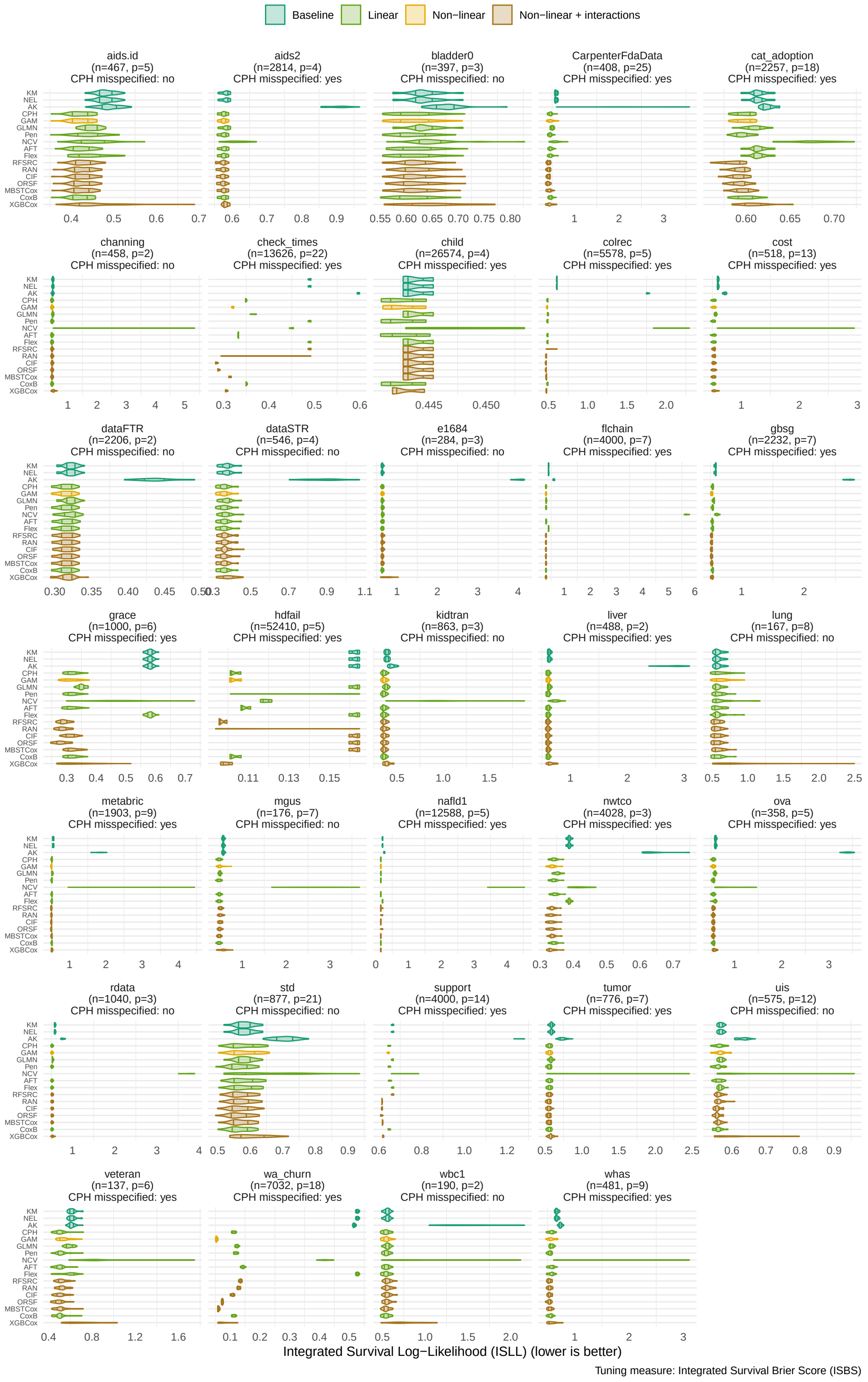

Figure 55: Per-dataset scores grouped by hypothesis space for learners tuned on ISBS and evaluated with ISLL (violin plot)

References

O. Aalen. Nonparametric Inference for a Family of Counting Processes. 6(4):701–726, 1978.

M. G. Akritas. Nearest Neighbor Estimation of a Bivariate Distribution Under Random Censoring. 22(3):1299–1327, 1994. ISSN 0090-5364. doi: 10.1214/aos/1176325630. URL `https://projecteuclid.org:443/euclid.aos/1176325630`.

A. M. Allen, T. M. Therneau, J. J. Larson, A. Coward, V. K. Somers, and P. S. Kamath. Nonalcoholic fatty liver disease incidence and impact on metabolic burden and death: A 20 year-community study. 67(5):1726–1736, 2018. ISSN 1527-3350 (Electronic). doi: 10.1002/hep.29546.

P. K. Andersen, O. Borgan, R. D. Gill, and N. Keiding. Introduction. In *Statistical Models Based on Counting Processes*, Springer Series in Statistics, pages 1–44. Springer US, 1993. doi: https://doi.org/10.1007/978-1-4612-4348-9.

A. Barnwal, H. Cho, and T. Hocking. Survival Regression with Accelerated Failure Time Model in XGBoost. 31(4):1292–1302, 2022. ISSN 1061-8600. doi: 10.1080/10618600.2022.2067548.

Beartooth Computing Environment, x86_64 cluster. Advanced research computing center, 2024. University of Wyoming, Laramie, WY.

A. Bender and F. Scheipl. pammtools: Piece-wise exponential Additive Mixed Modeling tools. 2018. URL `http://arxiv.org/abs/1806.01042`.

H. Binder and M. Schumacher. Allowing for mandatory covariates in boosting estimation of sparse high-dimensional survival models. 9(1):14, 2008. ISSN 1471-2105. doi: 10.1186/1471-2105-9-14.

M. Binder, F. Pfisterer, M. Lang, L. Schneider, L. Kotthoff, and B. Bischl. mlr3pipelines - flexible machine learning pipelines in r. 22(184):1–7, 2021. URL `http://jmlr.org/papers/v22/21-0281.html`.

P. Blanche, J.-F. Dartigues, and H. Jacqmin-Gadda. Estimating and comparing time-dependent areas under receiver operating characteristic curves for censored event times with competing risks. 32(30):5381–5397, 2013. ISSN 1097-0258. doi: 10.1002/sim.5958.

L. Breiman, editor. *Classification and Regression Trees*. Chapman & Hall/CRC, 1. crc press repr edition, 1998. ISBN 978-0-412-04841-8.

N. E. Breslow and N. Chatterjee. Design and Analysis of Two-Phase Studies with Binary Outcome Applied to Wilms Tumour Prognosis. 48(4):457–468, 1999. ISSN 0035-9254. doi: 10.1111/1467-9876.00165.

G. Broström. *eha: Event History Analysis*, 2021. URL `http://ehar.se/r/eha/`. R package version 2.9.0.

P. Bühlmann and B. Yu. Boosting With the L2 Loss: Regression and Classification. 98(462):324–339, 2003. ISSN 0162-1459. doi: 10.1198/016214503000125.

C. Cai, Y. Zou, Y. Peng, and J. Zhang. *smcure: Fit Semiparametric Mixture Cure Models*, 2012. URL `https://CRAN.R-project.org/package=smcure`. R package version 2.0.

B. P. Carlin and T. A. Louis. Supplemental Materials to Bayesian Methods for Data Analysis, 3rd Edition. 2018. doi: 10.13020/D6N10N.

D. P. Carpenter. Groups, the media, agency waiting costs, and fda drug approval. 46(3):490–505, 2002. ISSN 00925853, 15405907. URL `http://www.jstor.org/stable/3088394`.

T. Chen and C. Guestrin. XGBoost: A Scalable Tree Boosting System. In *Proceedings of the 22nd ACM SIGKDD International Conference on Knowledge Discovery and Data Mining*, KDD '16, pages 785–794. Association for Computing Machinery, 2016. ISBN 978-1-4503-4232-2. doi: 10.1145/2939672.2939785.

D. R. Cox. Regression Models and Life-Tables. 34(2):187–220, 1972.

L. Dirick, G. Claeskens, and B. Baesens. Time to default in credit scoring using survival analysis: A benchmark study. 68(6):652–665, 2017. ISSN 14769360. doi: 10.1057/s41274-016-0128-9.

A. Dispenzieri, J. A. Katzmann, R. A. Kyle, D. R. Larson, T. M. Therneau, C. L. Colby, R. J. Clark, G. P. Mead, S. Kumar, L. J. Melton 3rd, and S. V. Rajkumar. Use of nonclonal serum immunoglobulin free light chains to predict overall survival in the general population. 87(6):517–523, 2012. ISSN 1942-5546. doi: 10.1016/j.mayocp.2012.03.009. URL `https://www.ncbi.nlm.nih.gov/pubmed/22677072https://www.ncbi.nlm.nih.gov/pmc/articles/PMC3538473/`.

S. Fischer, J. Zobolas, R. Sonabend, M. Becker, M. Lang, M. Binder, L. Schneider, L. Burk, P. Schratz, B. C. Jaeger, S. A. Lauer, L. A. Kapsner, M. Mücke, Z. Wang, D. Pulatov, K. Ganz, H. Funk, L. Harutyunyan, P. Camilleri, P. Kopper, A. Bender, B. Zhou, N. German, L. Koers, A. Nazarova, and B. Bischl. Mlr3extralearners: Expanding the mlr3 Ecosystem with Community-Driven Learner Integration. 10(115):8331, 2025. ISSN 2475-9066. doi: 10.21105/joss.08331.

Y. Foucher, F. L. Borgne, C. Arthur, and C. Sabathe. *RISCA: Causal Inference and Prediction in Cohort-Based Analyses.*, 2023. URL `https://CRAN.R-project.org/package=RISCA`. R package version 1.0.4.

E. N. Georgousopoulou, C. Pitsavos, C. M. Yannakoulia, and D. B. Panagiotakos. Comparisons between Survival Models in Predicting Cardiovascular Disease Events : Application in the ATTICA Study (2002-2012). 4(2):203–210, 2015.

J. J. Goeman. L1 Penalized Estimation in the Cox Proportional Hazards Model. 52(1):70–84, 2010. ISSN 1521-4036. doi: 10.1002/bimj.200900028.

S. Goli, H. Mahjub, J. Faradmal, and A.-R. Soltanian. Performance Evaluation of Support Vector Regression Models for Survival Analysis: A Simulation Study. 7(6), 2016. ISSN 21565570, 2158107X. doi: 10.14569/IJACSA.2016.070650.

P. M. Grambsch and T. M. Therneau. Proportional hazards tests and diagnostics based on weighted residuals. *Biometrika*, 81(3):515–526, 1994. ISSN 0006-3444, 1464-3510. doi: 10.1093/biomet/81.3.515.

I. D. Ha, M. Noh, J. Kim, and Y. Lee. *frailtyHL: Frailty Models via Hierarchical Likelihood*, 2019. URL `https://CRAN.R-project.org/package=frailtyHL`. R package version 2.3.

D. Habibi, M. Rafiei, A. Chehrei, Z. Shayan, and S. Tafaqodi. Comparison of Survival Models for Analyzing Prognostic Factors in Gastric Cancer Patients. 19(3):749–753, 2018. ISSN 2476-762X. doi: 10.22034/APJCP.2018.19.3.749. URL `http://www.ncbi.nlm.nih.gov/pubmed/29582630{%}0Ahttp://www.pubmedcentral.nih.gov/articlerender.fcgi?artid=PMC5980851`.

M. Herrmann, P. Probst, R. Hornung, V. Jurinovic, and A.-L. Boulesteix. Large-scale benchmark study of survival prediction methods using multi-omics data. 22(3):bbaa167, 2021. ISSN 1477-4054. doi: 10.1093/bib/bbaa167.

D. W. Hosmer, S. Lemeshow, and S. May. *Applied survival analysis regression modeling of time-to-event data*. Wiley series in probability and statistics. Wiley-Interscience, 2nd ed. edition, 2008.

D. W. Hosmer Jr, S. Lemeshow, and S. May. *Applied survival analysis: regression modeling of time-to-event data*, volume 618. John Wiley & Sons, 2011.

T. Hothorn and A. Zeileis. partykit: A Modular Toolkit for Recursive Partytioning in R. 16: 3905–3909, 2015. URL `http://jmlr.org/papers/v16/hothorn15a.html`.

T. Hothorn, K. Hornik, and A. Zeileis. Unbiased Recursive Partitioning: A Conditional Inference Framework. 15(3):651–674, 2006. ISSN 1061-8600. doi: 10.1198/106186006X133933.

H. Ishwaran, U. B. Kogalur, E. H. Blackstone, and M. S. Lauer. Random survival forests. 2(3): 841–860, 2008. doi: 10.1214/08-AOAS169.

B. C. Jaeger, S. Welden, K. Lenoir, J. L. Speiser, M. W. Segar, A. Pandey, and N. M. Pajewski. Accelerated and Interpretable Oblique Random Survival Forests. 33(1):192–207, 2024. ISSN 1061-8600. doi: 10.1080/10618600.2023.2231048.

H. S. Jørgensen, H. Nakayama, J. Reith, H. O. Raaschou, and T. S. Olsen. Acute Stroke With Atrial Fibrillation. 27(10):1765–1769, 1996. doi: 10.1161/01.STR.27.10.1765.

J. D. Kalbfleisch and R. L. Prentice. *The Statistical Analysis of Failure Time Data*. John Wiley & Sons, 2011. ISBN 978-1-118-03123-0.

E. L. Kaplan and P. Meier. Nonparametric Estimation from Incomplete Observations. 53(282): 457–481, 1958. ISSN 01621459. doi: 10.2307/2281868.

M. W. Kattan. Comparison of Cox Regression With Other Methods for Determining Prediction Models and Nomograms. 170:S6–S10, 2003. ISSN 0022-5347. doi: 10.1097/01.ju.0000094764.56269.2d.

M. W. Kattan and T. A. Gerds. The index of prediction accuracy: An intuitive measure useful for evaluating risk prediction models. 2(1):7, 2018. ISSN 2397-7523. doi: 10.1186/s41512-018-0029-2.

J. L. Katzman, U. Shaham, A. Cloninger, J. Bates, T. Jiang, and Y. Kluger. DeepSurv: personalized treatment recommender system using a Cox proportional hazards deep neural network. 18(1): 24, 2018. ISSN 1471-2288. doi: 10.1186/s12874-018-0482-1. URL `https://doi.org/10.1186/s12874-018-0482-1`.

J. M. Kirkwood, M. H. Strawderman, M. S. Ernstoff, T. J. Smith, E. C. Borden, and R. H. Blum. Interferon alfa-2b adjuvant therapy of high-risk resected cutaneous melanoma: the eastern cooperative oncology group trial est 1684. 14(1):7–17, 1996.

J. P. Klein and M. L. Moeschberger. *Survival analysis: techniques for censored and truncated data*. Springer Science & Business Media, 2 edition, 2003. ISBN 0387216456.

R. Koenker. *quantreg: Quantile Regression*, 2021. URL `https://www.r-project.org`. R package version 5.86.

M. Kuhn. modeldata: Data sets useful for modeling examples, 2024. URL https://CRAN.R-project.org/package=modeldata.

H. Kvamme. pycox, 2018. URL https://pypi.org/project/pycox/.

R. A. Kyle. "Benign" monoclonal gammopathy–after 20 to 35 years of follow-up. 68(1):26–36, 1993. ISSN 0025-6196 (Print). doi: 10.1016/s0025-6196(12)60015-9.

M. Lang, B. Bischl, and D. Surmann. Batchtools: Tools for R to work on batch systems. 2(10): 135, 2017. ISSN 2475-9066. doi: 10.21105/joss.00135.

M. Lang, M. Binder, J. Richter, P. Schratz, F. Pfisterer, S. Coors, Q. Au, G. Casalicchio, L. Kotthoff, and B. Bischl. mlr3: A modern object-oriented machine learning framework in R. 4(44): 1903, 2019. doi: 10.21105/joss.01903. URL https://joss.theoj.org/papers/10.21105/joss.01903.

M. Lang, J. Richter, B. Bischl, and D. Schalk. mlr3tuning: Tuning for 'mlr3', 2023. URL https://cran.r-project.org/package=mlr3tuning.

P. L'Ecuyer. Good Parameters and Implementations for Combined Multiple Recursive Random Number Generators. 47(1):159–164, 1999. ISSN 0030-364X. doi: 10.1287/opre.47.1.159. URL https://pubsonline.informs.org/doi/abs/10.1287/opre.47.1.159.

S. Lee and H. Lim. Review of statistical methods for survival analysis using genomic data. 17(4): e41–e41, 2019. ISSN 1598-866X. doi: 10.5808/GI.2019.17.4.e41. URL https://pubmed.ncbi.nlm.nih.gov/31896241https://www.ncbi.nlm.nih.gov/pmc/articles/PMC6944043/.

C. L. Loprinzi, J. A. Laurie, H. S. Wieand, J. E. Krook, P. J. Novotny, J. W. Kugler, J. Bartel, M. Law, M. Bateman, and N. E. Klatt. Prospective evaluation of prognostic variables from patient-completed questionnaires. North Central Cancer Treatment Group. 12(3):601–607, 1994. ISSN 0732-183X (Print). doi: 10.1200/JCO.1994.12.3.601.

J. T. Luxhoj and H.-J. Shyur. Comparison of proportional hazards models and neural networks for reliability estimation. 8(3):227–234, 1997. ISSN 1572-8145. doi: 10.1023/A:1018525308809.

M. Pohar and J. Stare. Relative survival analysis in R. 81:272–278, 2006. doi: 10.1016/j.cmpb.2006.01.004.

U. B. Mogensen, H. Ishwaran, and T. A. Gerds. Evaluating Random Forests for Survival Analysis using Prediction Error Curves, 2014.

B. Moghimi-Dehkordi, A. Safaee, M. A. Pourhoseingholi, R. Fatemi, Z. Tabeie, and M. R. Zali. Statistical comparison of survival models for analysis of cancer data. 9(3):417–420, 2008. ISSN 2476-762X.

J. V. Monaco, M. Gorfine, and L. Hsu. General semiparametric shared frailty model: Estimation and simulation with frailtySurv. 86(4):1–42, 2018. doi: 10.18637/jss.v086.i04.

L. Ohno-Machado. A comparison of Cox proportional hazards and artificial neural network models for medical prognosis. 27(1):55–65, 1997. ISSN 0010-4825. doi: 10.1016/S0010-4825(96)00036-4.

L. Ohno-Machado. Modeling medical prognosis: survival analysis techniques. 34(6):428–439, 2001. ISSN 1532-0464 (Print). doi: 10.1006/jbin.2002.1038.

K. Patel, R. Kay, and L. Rowell. Comparing proportional hazards and accelerated failure time models: An application in influenza. 5(3):213–224, 2006. ISSN 1539-1612. doi: 10.1002/pst.213.

D. Rizopoulos. JM: An R Package for the Joint Modelling of Longitudinal and Time-to-Event Data. 35(9):1–33, 2010. URL http://www.jstatsoft.org/v35/i09/.

P. Royston and M. K. Parmar. Flexible parametric proportional-hazards and proportional-odds models for censored survival data, with application to prognostic modelling and estimation of treatment effects. 21(15):2175–2197, 2002. ISSN 02776715. doi: 10.1002/sim.1203.

N. Simon, J. H. Friedman, T. Hastie, and R. Tibshirani. Regularization Paths for Cox's Proportional Hazards Model via Coordinate Descent. 39:1–13, 2011. ISSN 1548-7660. doi: 10.18637/jss.v039.i05.

R. Sonabend. survivalmodels: Models for Survival Analysis, 2020. URL https://cran.r-project.org/package=survivalmodels.

R. Sonabend and F. Pfisterer. mlr3benchmark: Benchmarking analysis for 'mlr3', 2020. URL https://cran.r-project.org/package=mlr3benchmark.

R. Sonabend and P. Schratz. mlr3extralearners: Extra Learners For mlr3, 2024. URL https://github.com/mlr-org/mlr3extralearners.

R. Sonabend, F. J. Király, A. Bender, B. Bischl, and M. Lang. mlr3proba: An R Package for Machine Learning in Survival Analysis. 2021. ISSN 1367-4803. doi: 10.1093/bioinformatics/btab039. URL https://cran.r-project.org/package=mlr3proba.

A. Spooner, E. Chen, A. Sowmya, P. Sachdev, N. A. Kochan, J. Trollor, and H. Brodaty. A comparison of machine learning methods for survival analysis of high-dimensional clinical data for dementia prediction. 10(1):20410, 2020. ISSN 2045-2322. doi: 10.1038/s41598-020-77220-w. URL https://doi.org/10.1038/s41598-020-77220-w.

C. Strobl, F. Wickelmaier, and A. Zeileis. Accounting for Individual Differences in Bradley-Terry Models by Means of Recursive Partitioning. 36(2):135–153, 2011. ISSN 1076-9986, 1935-1054. doi: 10.3102/1076998609359791.

R. J. Sylvester, A. P. van der Meijden, W. Oosterlinck, J. A. Witjes, C. Bouffioux, L. Denis, D. W. Newling, and K. Kurth. Predicting recurrence and progression in individual patients with stage ta T1 bladder cancer using EORTC risk tables: A combined analysis of 2596 patients from seven EORTC trials. 49(3):466–477, 2006. ISSN 0302-2838. doi: 10.1016/j.eururo.2005.12.031.

The Benelux C M L Study Group. Randomized Study on Hydroxyurea Alone Versus Hydroxyurea Combined With Low-Dose Interferon-α2b for Chronic Myeloid Leukemia. 91(8):2713–2721, 1998. ISSN 1528-0020. doi: 10.1182/blood.V91.8.2713.2713_2713_2721. URL https://doi.org/10.1182/blood.V91.8.2713.2713{_}2713{_}2721https://ashpublications.org/blood/article/91/8/2713/107615/Randomized-Study-on-Hydroxyurea-Alone-Versus.

K. Trébern-Launay, M. Giral, J. Dantal, and Y. Foucher. Comparison of the risk factors effects between two populations: Two alternative approaches illustrated by the analysis of first and second kidney transplant recipients. 13:102, 2013. ISSN 1471-2288. doi: 10.1186/1471-2288-13-102.

H. Turner, I. Kosmidis, and D. Firth. PlackettLuce: Plackett-Luce Models for Rankings. Comprehensive R Archive Network, 2017.

H. L. Turner, J. Van Etten, D. Firth, and I. Kosmidis. Modelling rankings in R: The PlackettLuce package. 35(3):1027–1057, 2020. ISSN 0943-4062, 1613-9658. doi: 10.1007/s00180-020-00959-3.

K. Ushey and H. Wickham. *renv: Project Environments*, 2024. URL `https://CRAN.R-project.org/package=renv`. R package version 1.0.7.

V. Van Belle, K. Pelckmans, S. Van Huffel, and J. A. K. Suykens. Support vector methods for survival analysis: A comparison between ranking and regression approaches. 53(2):107–118, 2011. ISSN 0933-3657. doi: 10.1016/j.artmed.2011.06.006.

J. C. Van Houwelingen, W. W. ten Bokkel Huinink, M. E. Van der Burg, A. T. Van Oosterom, and J. P. Neijt. Predictability of the survival of patients with advanced ovarian cancer. 7(6):769–773, 1989. ISSN 0732-183X.

W. N. Venables and B. D. Ripley. *Modern Applied Statistics with S.* Statistics and Computing. Springer, 4th ed edition, 2002. ISBN 978-0-387-95457-8.

P. Wang, Y. Li, and C. K. Reddy. Machine Learning for Survival Analysis: A Survey. 51(6): 110:1–110:36, 2019. ISSN 0360-0300. doi: 10.1145/3214306.

S. Wiegrebe, P. Kopper, R. Sonabend, B. Bischl, and A. Bender. Deep learning for survival analysis: A review. 57(3):65, 2024. ISSN 1573-7462. doi: 10.1007/s10462-023-10681-3.

P. Williamson, R. Kolamunnage-Dona, P. Philipson, and A. G. Marson. Joint modelling of longitudinal and competing risks data. 27:6426–6438, 2008.

D. Wissel, D. Rowson, and V. Boeva. Systematic comparison of multi-omics survival models reveals a widespread lack of noise resistance. 3(4):100461, 2023. ISSN 26672375. doi: 10.1016/j.crmeth.2023.100461.

S. N. Wood, N. Pya, and B. Säfken. Smoothing parameter and model selection for general smooth models (with discussion). 111:1548–1575, 2016. doi: 10.1080/01621459.2016.1180986.

M. N. Wright and A. Ziegler. ranger: A Fast Implementation of Random Forests for High Dimensional Data in C++ and R. 77(1):1–17, 2017.

A. Zare, M. Hosseini, M. Mahmoodi, K. Mohammad, H. Zeraati, and K. Holakouie Naieni. A Comparison between Accelerated Failure-time and Cox Proportional Hazard Models in Analyzing the Survival of Gastric Cancer Patients. 44(8):1095–102, 2015. ISSN 03044556. doi: 10.1007/s00606-006-0435-8. URL `http://www.ncbi.nlm.nih.gov/pubmed/26587473{%}0Ahttp://www.pubmedcentral.nih.gov/articlerender.fcgi?artid=PMC4645729`.

Y. Zhang, G. Wong, G. Mann, S. Muller, and J. Y. H. Yang. SurvBenchmark: Comprehensive benchmarking study of survival analysis methods using both omics data and clinical data. page 2021.07.11.451967, 2021. doi: 10.1101/2021.07.11.451967.
